# Localized polarization-guided alignment control in carbon nanotube films via femtosecond-pulse optical post-processing

**DOI:** 10.1038/s41467-026-76305-w

**Published:** 2026-08-01

**Authors:** Daichi Suzuki, Yung-Chang Lin, Yuma Takida, Hideaki Nakajima, Kaori Fujii, Takaaki Abe, Naoko Kurihira, Teppei Araki, Masanori Koshino, Toshiya Okazaki, Hiroaki Minamide, Nao Terasaki, Taishi Nishihara

**Affiliations:** 1 Sensing Technology Research Institute, National Institute of Advanced Industrial Science and Technology (AIST), Saga, Japan; 2 Research Institute of Core Technology for Materials Innovation, National Institute of Advanced Industrial Science and Technology (AIST), Ibaraki, Japan; 3 RIKEN Center for Advanced Photonics, RIKEN, Miyagi, Japan; 4 Nanocarbon Material Research Institute, National Institute of Advanced Industrial Science and Technology (AIST), Ibaraki, Japan; 5 SANKEN (The Institute of Scientific and Industrial Research), The University of Osaka, Osaka, Japan; 6 Department of Physics, Tokyo University of Science, Tokyo, Japan; 7 Research Institute for Science and Technology, Tokyo University of Science, Tokyo, Japan

**Keywords:** Carbon nanotubes and fullerenes, Carbon nanotubes and fullerenes

## Abstract

The physical properties of single-walled carbon nanotubes (CNTs) are highly sensitive to their alignment; therefore, making precise alignment control a central requirement for high-performance device architectures. However, conventional alignment techniques offer limited spatial programmability and remain inadequate for integrating heterogeneous CNT structures within a single film. Here, we report an optical post-processing technique to locally control the alignment of CNTs. We demonstrate that femtosecond-pulsed laser processing enables polarisation-guided alignment control of CNTs after film formation (post-processing). This process produces sub-micrometre scale locally aligned CNTs with thickness exceeding 200 nm and a nematic order parameter of 0.93 (strong directional order), demonstrating a high degree of orientational order and addressing a key bottleneck in spatially heterogeneous CNT integration. Additionally, these laser-aligned CNTs exhibit a direction-dependent optical and electrical response, functioning as a terahertz polariser with a polarisation ratio of 24.7; therefore, this technique enables the applications of CNT devices integrating heterogeneous structures like division-of-focal-plane polarisation cameras and on-chip polarisation routing.

## Abstract (web summary)

Precise alignment is critical for engineering high-performance carbon nanotubes-based devices. Here, the authors develop a femtosecond-pulse optical postprocessing technique to locally control the alignment of carbon nanotubes.

## Introduction

Single-walled carbon nanotubes (CNTs) possess quantum properties owing to their one-dimensional nanostructures, which make them promising nanomaterials for practical applications that surpass conventional bulk semiconductors. Since their discovery by Iijima in 1991^1^, CNTs have been the subject of extensive research. Beginning with fundamental studies on growth methods and optical properties, research has expanded to include chemical surface modification and doping techniques, and applications such as reinforcement and thermoelectric generation devices^2–8^. We have also recently reported broadband sensors and terahertz (THz) sensing applications that utilise the one-dimensional plasmon resonance of CNTs, which provides absorption from the THz to the infrared bands^9–16^.

Despite more than 30 years of research, processing difficulties have hindered the practical application of CNTs in electronics. Their physical properties vary significantly depending on their alignment^17–19^. Therefore, proper control of these nanostructures is essential for device applications. Established alignment techniques are summarised in reviews^20,21^. Techniques for alignment control—such as shearing, vacuum filtration, electrophoresis, spin coating, liquid crystalline effects, self-alignment, polymer wrapping, CVD alignment, lithographically defined microscopic water features, soft-lock method, and DNA nanotrenches—have been reported^22–32^.

Although various nanostructure control techniques based on mechanical, chemical, and electrical mechanisms have been developed, they are all implemented during the pre-processing stage of device fabrication, such as synthesis or filtration. Consequently, spatial control is limited, and these conventional techniques cannot locally combine different orientations. Therefore, the development of new nanostructure control techniques that enable position control is key to the practical application of CNT devices.

Against this background, this paper presents an optical post-processing technique for the local control of CNT alignment using femtosecond-pulsed lasers via non-thermal processing. We focus on the following four objectives: (i) demonstrating local alignment by polarisation-controlled femtosecond-pulse irradiation, (ii) elucidating CNTs fracture behaviour using transmission electron microscopy (TEM) observations, (iii) quantifying the lateral feature size and alignment degree (nematic order parameter), and (iv) determining the processed depth and its dependence on pulse parameters and film thickness using optical and electrical anisotropy as depth probes. This technology is fundamentally different from conventional techniques in that it is an ‘optical’ nanostructure control method, allowing localised alignment modification only in the laser-irradiated area. Therefore, this method is expected to resolve the aforementioned positional control limitations and become a technology for practical CNT devices, such as division-of-focal-plane THz polarisation imaging pixels and a THz radial polariser.

## Results and discussion

### Local alignment control via laser irradiation

As a starting point for this technology, we introduce the material removal mechanisms following laser irradiation—laser ablation^33^. When a laser is irradiated onto a CNT, it absorbs photon energy, resulting in electron excitation and a localised elevation in electron temperature (associated with the thermal kinetic energy of electrons). Subsequently, energy transfer occurs through electron-phonon collisions, which raises the lattice temperature (thermal kinetic energy of phonons). The interval during which this energy exchange transpires is referred to as the electron-phonon relaxation time, typically ranging from 1 to 100 ps for most materials^34,35^. Once either the electron or lattice temperature surpasses the material’s melting point, evaporation ensues, followed by plasma formation and material removal—a process central to laser ablation. A critical parameter here is the relationship between laser pulse duration (pulse width) and the electron-phonon relaxation time. If the pulse width is shorter than the relaxation time (e.g. femtoseconds to a few picoseconds), electron-phonon collision relaxation does not occur. Consequently, only the electron temperature increases, leading to material vaporisation, plasma generation, and subsequent removal at the electron temperature. In this scenario, phonon-mediated heat conduction is absent; thus, it is generally classified as ‘non-thermal processing’. Conversely, under longer pulse durations—such as nanosecond pulses or continuous waves—the destructive phenomenon follows the electron-phonon collision, and material removal occurs at the lattice temperature via thermal evaporation, encompassing conventional conductive heat transfer (‘thermal processing’).

**Fig. 1:**
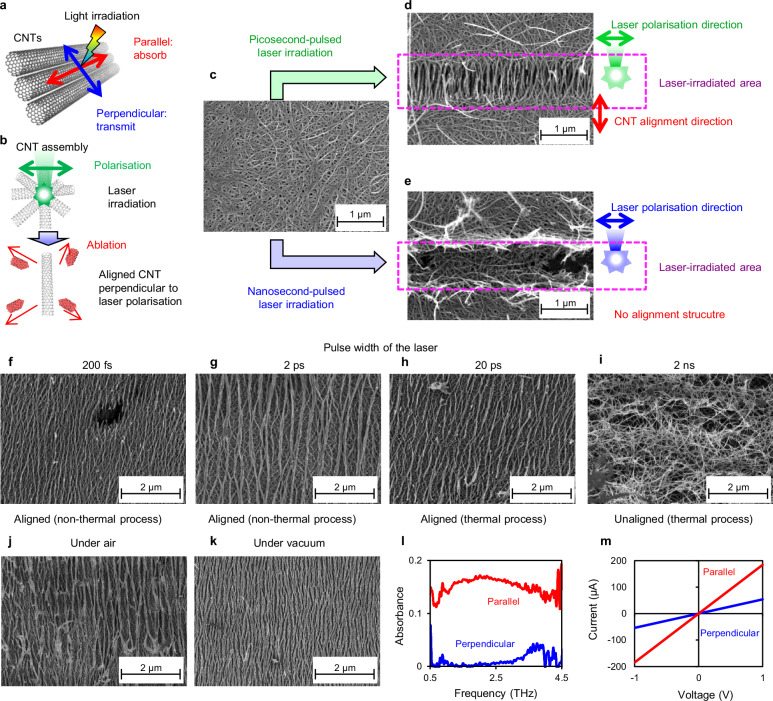
Local carbon nanotube (CNT) alignment control via laser irradiation. **a** Polarisation properties of CNTs. **b** Schematic illustration of alignment control by laser irradiation. **c**–**e** Scanning electron microscopy (SEM) images of the CNT film (**c**) before and after laser irradiation with (**d**) picosecond-pulse and (**e**) nanosecond pulse. **f**–**i** SEM images of the laser-irradiated CNTs with the different laser pulse widths of (**f**) 200 fs, (**g**) 2 ps, (**h**) 20 ps, and (**i**) 2 ns. **j, k** SEM images of the laser-aligned CNTs performed under (**j**) air and (**k**) vacuum. **l, m** (**l**) Optical and (**m**) electrical properties of the laser-aligned CNT film. Source data are provided as a Source data file.

By utilising the laser ablation phenomenon, we conducted localised CNT alignment using femtosecond- and picosecond-pulsed laser irradiation. Figure 1a, b illustrates the laser-induced alignment formation mechanism. CNTs, which are one-dimensional structures, are known to exhibit polarisation properties^36^, absorbing parallel light polarisation better than perpendicular light polarisation to their long axis (Fig. 1a). We focused on the anisotropy of optical absorption and proposed an alignment process for CNT films that utilises these polarisation properties. As shown in Fig. 1b, irradiating a non-aligned CNT assembly with a polarised pulsed laser selectively laser-ablated only the parallel CNTs that absorbed the laser. Consequently, only the perpendicular CNTs with lower absorption remained, forming an aligned structure. Figure 1c, d shows the scanning electron microscopy (SEM) images before and after irradiating the CNT assembly with a picosecond-pulsed laser. The laser parameters were as follows: wavelength of 532 nm, pulse width of <10 ps, spot diameter of 927 nm, fluence of 30 mJ cm^−^², line scanning with scan speed of 3 mm s^−1^, repetition rate of 1 MHz, and horizontal polarisation. The CNTs form an aligned structure only in the areas irradiated with the picosecond-pulsed laser (indicated by the purple squares in Fig. 1d). The CNT alignment direction was perpendicular to the laser polarisation direction, confirming the processing principle illustrated in Fig. 1b. The alignment structure of the CNTs was also confirmed by TEM and atomic force microscopy (AFM) as shown in Supplementary Figs. 1, and 2. Since individual CNTs cannot be observed in SEM images, the alignment structures observed in SEM are CNT bundles with a diameter of 20–50 nm (Supplementary Fig 2). Importantly, the alignment structure was formed locally only within the laser-irradiated area, and a spatial resolution of 600 nm was achieved using an objective lens with a numerical aperture (NA) of 0.7 (estimated spot diameter: 927 nm). This result represents the creation of a process technology that enables local alignment control from CNT assembly to post-processing.

We investigated how the laser parameters influenced the alignment processing phenomena. As mentioned in the previous section, the most important parameter in laser ablation is the pulse width, which determines the destructive phenomenon. Figure 1e shows the processed image when the pulse width was changed from picoseconds to nanoseconds. The laser polarisation was horizontal, as in the case of picosecond pulses. However, unlike in Fig. 1d, the processed area shows no alignment structure and simply cuts the laser-irradiated area. According to the general understanding of laser ablation^33^, this result depends on whether the processing phenomenon is thermal or non-thermal. To identify the threshold for non-thermal processing, we systematically investigated the pulse width dependence. Figure 1f–i presents the alignment quality when the pulse width is varied from 200 fs to 2 ns. For 200 fs and 2 ps pulses, alignment is achieved through non-thermal ablation. When a 20-ps pulse is used, alignment occurs; however, carbonized debris due to thermal melting appears at high magnification. In contrast, with a 2 ns pulse, all CNTs are removed irrespective of laser polarisation due to thermal processing. Based on these experimental results, we discuss the underlying mechanism of laser-induced alignment. When CNTs absorb light, electrons are first excited through photon–electron coupling. The excited electrons then transfer energy to optical phonons through electron–phonon scattering. This process has been reported to occur on an ultrafast sub-100-fs timescale^37,38^. It should be noted that optical phonons play an important role in interactions with electromagnetic radiation (light), but they contribute only minimally to thermal diffusion. The energy stored in optical phonons is subsequently transferred to acoustic phonons through phonon–phonon scattering. This process has been reported to occur on a timescale of several picoseconds^37,38^. Unlike optical phonons, acoustic phonons contribute directly to thermal diffusion in the material. Therefore, material removal on a timescale faster than acoustic phonon relaxation, i.e. within several picoseconds, is generally required for non-thermal processing^33^. Based on these physical phenomena, aligned structures are expected to form when the laser pulse duration is 200 fs or 2 ps via non-thermal processing. In contrast, 20-ps or 2-ns pulses should not form aligned structures because the process is governed by thermal mechanisms. However, our pulse-duration-dependent experimental results (Fig. 1f–i) show that aligned CNT structures were still formed even with a pulse duration of 20 ps, which is longer than the acoustic phonon relaxation time in CNTs. This apparently anomalous observation indicates that the critical timescale governing successful alignment is not simply the electron–phonon relaxation time, but rather the thermal diffusion time between adjacent CNTs. The acoustic phonon scattering process described above determines the timescale of the lattice temperature rise in laser-absorbing CNTs. In contrast, thermal diffusion between CNTs proceeds through dispersants, voids, and bundle contact points, and therefore occurs on a much longer timescale than acoustic phonon scattering. Thus, successful laser-induced alignment is governed by whether the laser-absorbing CNTs can be selectively removed before heat diffuses to surrounding CNTs. The reason why aligned processing can be achieved with a pulse duration of 20 ps (Fig. 1h), which is longer than the acoustic phonon scattering time, is that although the lattice temperature of the laser-absorbing CNTs increases (thermal process), CNT removal occurs within a time scale shorter than the thermal diffusion into surrounding CNTs. However, because processing at 20 ps is governed by thermal mechanisms, the resulting processing quality is inferior to that obtained under non-thermal processing using pulse durations of 200 fs and 2 ps. Although the CNT–CNT thermal diffusion time is difficult to determine precisely because it strongly depends on the film morphology, including density, bundle diameter, and dispersant content, our experimental results in Fig. 1f–i indicates that when the relevant timescale is 10 ps or shorter, only the laser-absorbing CNTs can be removed before substantial heat diffusion to the surrounding environment occurs, thereby enabling the formation of aligned CNT structures. Taken together, this study reveals that (i) successful laser-induced alignment is determined by the removal of laser-absorbing CNTs prior to thermal diffusion into surrounding CNTs, (ii) the characteristic time required for heat diffusion from laser-absorbing CNTs to their surroundings is ≈10 ps, and (iii) when the pulse duration is shorter than this threshold (≤10 ps), effective alignment processing can be achieved through a predominantly non-thermal process.

We investigated dependence on the external environment and generalisability of the laser alignment technology that we developed. All the experiments were performed under consistent laser irradiation parameters (pulse width: 200 fs; wavelength: 1030 nm; fluence: 125 mJ cm^−^²). First, we report on the dependence on the processing atmosphere. Figure 1j, k presents SEM images of laser-aligned CNTs processed under air and vacuum. While CNTs processed under atmospheric conditions exhibited burrs as processing marks, CNTs processed under vacuum formed a clean and fine alignment structure. While similar experiments were conducted under an inert gas (argon), the quality of CNTs processed under argon was comparable to that processed under atmospheric conditions (Supplementary Fig. 3). These findings indicate that (i) because it is a non-thermal process, the gas species (e.g. oxygen) does not affect the alignment quality, and (ii) the presence of gas molecules tends to compromise quality. Additionally, we examined the thermal conduction effects between CNT-CNT and CNT-substrate interfaces by systematically varying the synthesis methods, dispersant, and substrate (Supplementary Figs. 4–6). Thus, we confirmed that alignment processing could be performed similarly under all conditions, demonstrating the generalisability of the developed laser alignment technology.

As mentioned above, the minimum resolution of alignment processing was 600 nm (using an objective lens with an NA of 0.7 and the wavelength of 532 nm), while the maximum width can be expanded indefinitely via snake scanning (Supplementary Fig. 7). This scanning method enables the formation of large-area aligned CNT films with anisotropic properties. The resulting laser-aligned film exhibits direction-dependent optical (Fig. 1g) and electrical (Fig. 1h) responses, achieving an absorptance anisotropy ratio of 24.7 at 2 THz and an electrical conductivity anisotropy ratio of 3.43 (see ‘Methods’ for the definition of anisotropy ratio).

**Fig. 2:**
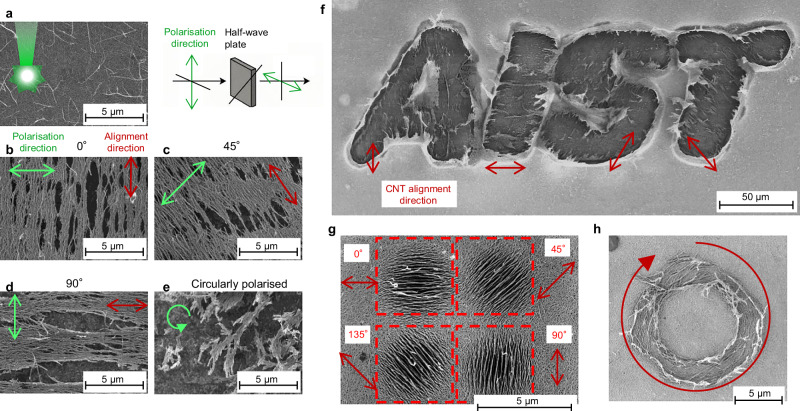
Relationship between carbon nanotube (CNT) alignment direction and laser polarisation direction. **a**–**e** Surface morphology of CNT films (**a**) without and with (**b**) 0°, (**c**) 45°, (**d**) 90°, and (**e**) circularly polarised pulsed laser irradiation. **f**–**h** Examples of CNT films with integrated heterogeneous alignment structures such as (**f**) micro-sized words, (**g**) integrated CNTs with four distinct orientations (0°, 45°, 90°, and 135°), and (**h**) circularly aligned structure.

Furthermore, because this technology utilises polarisation characteristics, the alignment direction of the CNTs can be controlled by manipulating the laser polarisation using a half-wave plate (Fig. 2a). Figure 2b–d shows the dependence of the processed shape on laser polarisation (0°, 45°, or 90°)—indicated with green arrows. The CNT alignment direction could also be changed to the perpendicular direction (90°, 135°, or 0°). Furthermore, when using a quarter-wave plate to circularly polarise the laser (Fig. 2e), all CNTs absorbed the laser, resulting in no alignment structure and simple cutting. These results strongly support the principle of alignment processing (Fig. 1b).

As mentioned previously, we demonstrated local alignment control using femtosecond-pulsed laser irradiation. Compared with conventional alignment techniques, the greatest advantage of this method is its local controllability, which enables the fabrication of CNT films with integrated heterogeneous alignment structures. By controlling the laser irradiation position and polarisation direction, we can fabricate nanostructures with a high degree of freedom that is unattainable using conventional techniques, such as micro-sized words (Fig. 2f), integrated CNTs with four distinct orientations (0°, 45°, 90°, and 135°) (Fig. 2g), and circularly aligned structures (Fig. 2h). This technique is capable of fabricating such complex nanostructures and is expected to be applied in electronics and photonic nanomaterials based on aligned CNTs.

### Quality of laser-aligned CNT films

**Fig. 3:**
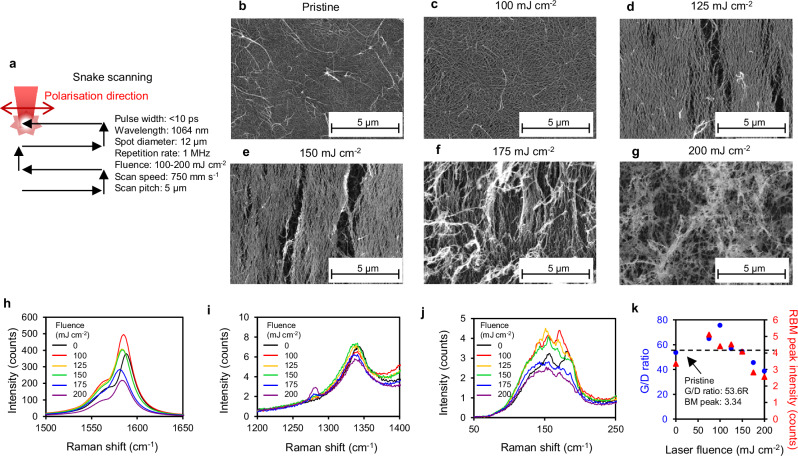
Raman spectroscopy of laser-aligned carbon nanotubes (CNTs). **a** Schematics of the laser irradiation condition. **b**–**g** Scanning electron microscopy images of CNTs irradiated with laser fluence of (**b**) 0, (**c**) 100 mJ cm^−2^, (**d**) 125 mJ cm^−2^, (**e**) 150 mJ cm^−2^, (**f**) 175 mJ cm^−2^, and (**g**) 200 mJ cm^−2^. **h**–**j** Enlarged Raman spectra of the (**h**) G band, (**i**) D band, and (**j**) radial breathing mode (RBM) peak. **k** Change in G/D ratio and RBM peak after laser irradiation for each fluence. Source data are provided as a Source data file.

In the previous section, we demonstrated the formation of geometrically aligned structures using femtosecond-pulsed lasers. In this section, we discuss the quality of laser-aligned CNT films using SEM and TEM observations and Raman spectroscopy. First, we studied the impact of the laser fluence on CNT morphology and crystalline quality (defects). Figure 3a illustrates the laser irradiation conditions, and Fig. 3b–g presents the SEM images at varying laser fluences. Compared to the pre-irradiation state (Fig. 3b), a slightly clearer image was observed; however, no aligned structures were observed at fluences of 100 mJ cm^−2^, which is lower than the laser ablation threshold (Fig. 3c). Subsequently, above the laser ablation threshold, CNTs parallel to the laser polarisation were laser-ablated, forming aligned structures (Fig. 3d, e). Further increases in fluence caused carbonisation, resulting in a brittle structure (Fig. 3f) and, ultimately, amorphous carbon waste (Fig. 3g). Regarding the corresponding Raman spectra, the G-mode (Fig. 3h), D-mode (Fig. 3i), and radial breathing mode (RBM) features (Fig. 3j) are also changed at varying laser fluence. It is noteworthy that the intensity ratio of the G-mode feature to the D-mode feature (G/D ratio), which is an indicator of crystalline quality, increased following laser irradiation at a fluence of 125 mJ cm^−2^ (Fig. 3k), where an aligned structure was formed (Fig. 3d), indicating that CNT crystalline quality was improved by laser irradiation. The G/D ratio decreased at a fluence above 125 mJ cm^−2^; this is due to carbonisation (Fig. 3f, g), which is supported by the appearance of the side peak at 1280 cm^−1^ (Fig. 3i). Thus, laser irradiation improves the crystalline quality of CNTs in addition to the control alignment. A comparative experiment was conducted using a circularly polarised laser to confirm whether this increase in G/D ratio originated from the alignment structure (Supplementary Fig. 8). We observed that an increase in the G/D ratio due to laser irradiation was confirmed even when a circularly polarised laser was irradiated, which does not create an alignment structure. These findings indicate that the G/D ratio enhancement following laser irradiation is not attributable to the alignment of the CNT structure.

**Fig. 4:**
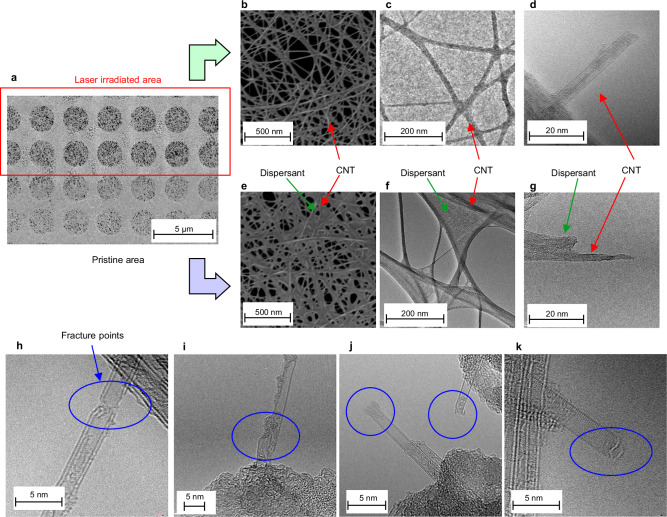
Scanning electron microscopy and transmission electron microscopy images of laser-irradiated carbon nanotubes (CNTs). **a** Overview of the entire region, and **b**–**g** magnified views of CNTs subjected to low-fluence laser irradiation. **h–k** CNTs subjected to high-fluence laser irradiation. The blue circles indicate the fracture points caused by the laser.

We investigated the mechanism behind the improved G/D ratio using TEM to examine the effects of laser irradiation on CNTs. The same TEM grid was used throughout the experiment (Fig. 4a), with results from low-fluence laser irradiation presented in Fig. 4b–g. The fluence was below the ablation threshold; therefore, the CNTs remained intact after exposure. Notably, the dispersant associated with the CNTs, which are indicated by green arrows in Fig. 4e–g, was removed after laser irradiation. Earlier, we reported that the CNT structure became slightly clearer in the SEM image with low-fluence irradiation (Fig. 3c), which is possibly due to the removal of the dispersant by the laser. The fracture behaviour at increased fluence is illustrated in Fig. 4h–k, with blue circles indicating CNT fracture points. The CNTs exhibited clean, localised fractures characteristic of their lattice structure, without substantial defects outside the fracture regions. With a 1 μm laser spot irradiating the entire CNT, fracturing occurred precisely rather than as an entire disintegration, which is particularly noteworthy.

We propose the following explanation: Laser irradiation excites electrons within the CNTs. In the presence of pre-existing defects, these electrons tend to localise around such defects, resulting in locally elevated electron temperatures and subsequent increases in thermal kinetic energy. This process initiates ablation primarily at defect sites. Analogous electron localisation has been reported in materials like graphene^39,40^, supporting the notion that fracture progresses preferentially from defective CNTs. Based on these SEM and TEM observations, we infer that: i) laser irradiation removes the CNT dispersant, yielding isolated CNTs, and ii) fracture is initiated at defect sites, thereby enriching the proportion of highly crystalline CNTs. We hypothesise that the enhanced G/D ratio arises from dispersant removal and selective elimination of defective CNTs. We are currently investigating the detailed mechanism of dispersant removal using photoinduced force microscopy^41^. Although the detailed mechanism remains under investigation, we conclude from the G/D ratio in Fig. 3d that there is no laser-induced damage to the laser-aligned CNTs at appropriate fluences.

### Alignment degree of laser-aligned CNT films

**Fig. 5:**
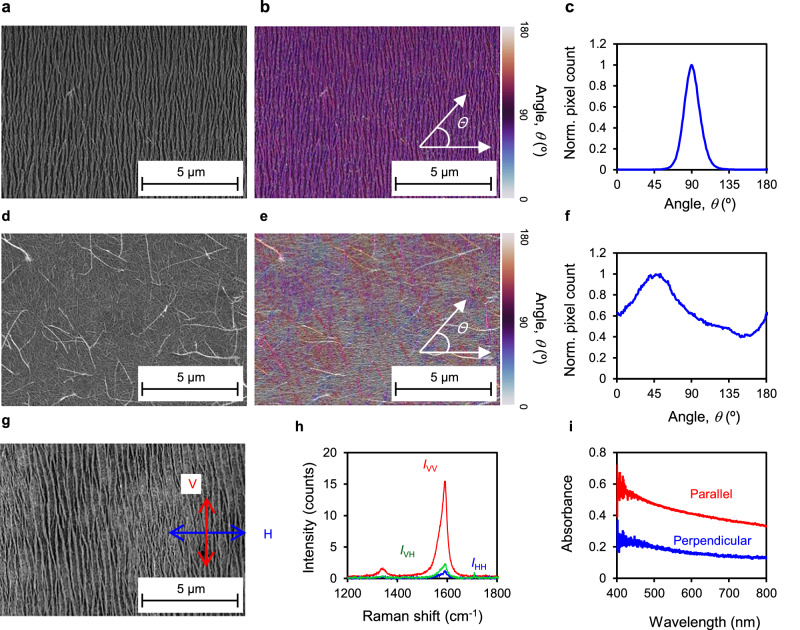
Evaluation of alignment degree. **a–f** Evaluation of alignment degree through image analysis. **a** SEM image, (**b**) colormap for orientation, and (**c**) distribution for orientation of the laser-aligned carbon nanotubes (CNTs). **d**–**f** **d** Scanning electron microscopy (SEM) image, (**e**) colormap for orientation, and (**f**) distribution for orientation of the pristine CNTs. **g–i** Evaluation of alignment degree through polarised Raman spectroscopy. **g** SEM image, (**h**) Raman spectra measured in VV, VH, and HH polarisations (polariser and analyser both parallel or perpendicular to the alignment axis), and (**i**) polarised absorbance characteristics in the visible frequency region. Source data are provided as a Source data file.

Next, we evaluated the alignment degree of laser-aligned CNTs by calculating the nematic order parameter *S* based on image analysis and polarised Raman spectroscopy. Figure 5a–f presents the degree of alignment evaluated from the image analysis, where we used the 500-nm-thick CNTs. Connected pixel components were extracted based on angular orientation using SEM images of laser-aligned CNTs. The alignment colormaps and distributions of the laser-aligned CNTs demonstrate a pronounced vertical alignment (Fig. 5b, c). Conversely, the pristine CNTs without laser treatment exhibited disordered results (Fig. 5d–f). The two-dimensional (2D) order parameter *S* was determined using the angular data and calculated as *S* = 0.93 using Eq. (1)^42^:

1 $$S=\left\langle 2{\cos }^{2}\theta -1\right\rangle$$

These results demonstrate a very high level of alignment compared to existing alignment techniques^21^. The results of the image analysis indicate the degree of alignment in the CNT surface. Subsequently, the alignment degree from the surface to a depth of ≈10 nm (corresponding to the light penetration depth) was assessed using polarised Raman spectroscopy and polarised visible spectroscopy. The experimental results are presented in Fig. 5g–i. For Raman spectroscopy, the excitation and detection light polarisations were controlled via a linear polariser, enabling measurements of peak intensities for the G band *I*_VV_, *I*_VH_, and *I*_HH_ (Fig. 5h). The excitation wavelength used for Raman analysis was 532 nm. Observed peak intensities for the G band were *I*_VV_ = 15.4, *I*_VH_ = 2.3, and *I*_HH_ = 1.6, indicating pronounced polarisation characteristics. Additionally, the absorbance spectrum within the visible range (400–800 nm) was measured (Fig. 5i), revealing that, similarly to the THz region, laser-aligned CNTs function as polarisers in the visible spectrum. Based on these observations, the dichroic ratio Abs/Pol (absorbance ratio relative to polarisation) at a 532 nm wavelength was determined as 2.34. Finally, the nematic order parameter *S* was calculated. According to the polarised Raman intensities (*I*_VV_, *I*_VH_, and *I*_HH_) and the dichroic ratio Abs/Pol*,* 2D order parameter *S* (Eq. (1)) can be calculated as follows^42^:

2 $$S=\frac{({{{\rm{Abs}}}}/{{{\rm{Pol}}}}){I}_{{{{\rm{VV}}}}}-{I}_{{{{\rm{HH}}}}}}{({{{\rm{Abs}}}}/{{{\rm{Pol}}}}){I}_{{{{\rm{VV}}}}}+{I}_{{{{\rm{HH}}}}}+(1+({{{\rm{Abs}}}}/{{{\rm{Pol}}}})){I}_{{{{\rm{VH}}}}}}.$$

Applying the experimental data to Eq. (2) yielded a 2D order parameter *S* of 0.76. This value is marginally lower than the surface alignment value (*S* = 0.93) determined via image analysis (Fig. 5a–f), suggesting that alignment degree of CNTs decreases with increasing film depth, likely due to the strong surface alignment induced by laser alignment technology.

**Fig. 6:**
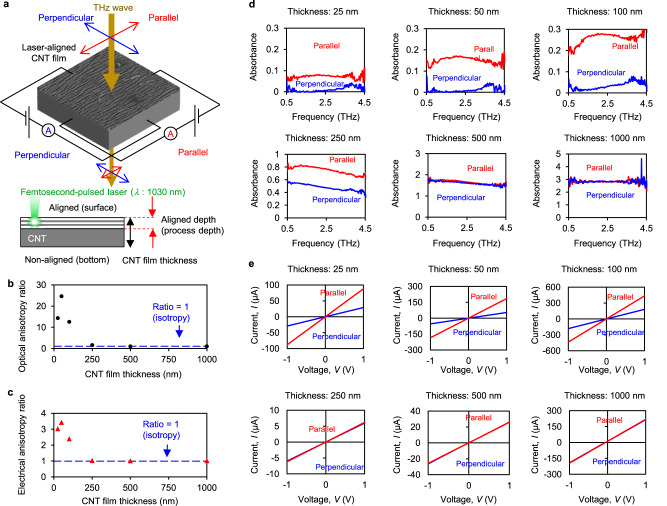
Optical and electrical properties of the laser-aligned carbon nanotubes (CNTs). **a** Schematic diagrams of the measurement systems. **b** Optical anisotropy of the laser-aligned CNT film. **c** Electrical anisotropy of the laser-aligned CNT film. **d**, **e** Detailed data of (**d**) THz absorbance spectra and (**e**) *I*–*V* curves of the laser-aligned CNTs. Source data are provided as a Source data file.

The nematic order parameter *S* calculated using image analysis and polarised Raman spectroscopy suggests that the alignment structure induced by femtosecond-pulses is applied only to the surface of the CNT film. Therefore, we evaluated the depth at which the alignment structure is formed, i.e. the depth of processing by laser alignment, based on the optical and electrical properties of the laser-aligned CNTs. CNT assemblies with film thicknesses ranging from 25 to 1000 nm were subjected to laser irradiation. The alignment depth was evaluated by measuring the anisotropy of the laser-aligned CNT film’s THz absorbance and electrical conductivity, which served as depth probes (Fig. 6a). Figure 6b–e shows the optical and electrical anisotropies of a laser-aligned CNT film irradiated with a laser wavelength of 1030 nm, a laser fluence of 150 mJ cm^-2^, using an objective lens (NA: 0.26, spot diameter: 4.8 μm), under vacuum conditions. Figure 6d,e shows the optical and electrical properties of laser-aligned CNTs when the thickness was 25–1000 nm. The blue (red) lines indicate the optical and electrical properties in the direction perpendicular (parallel) to the CNT alignment direction. As shown in Fig. 6b, c, in CNT films with thicknesses of 500 and 1000 nm, the optical and electrical properties were isotropic (anisotropy ratio = 1) despite the surface alignment structure. When the film thickness was decreased to 250 nm, direction-dependent optical and electrical responses appeared. The anisotropy ratio increased as the film thickness reduced, reaching 24.7 for the optical anisotropy ratio and 3.4 for the electrical anisotropy ratio at a film thickness of 50 nm. This result suggests that the alignment depth (the depth to which the laser penetrated) under these processing conditions was ≈250 nm. Therefore, optical and electrical anisotropies can be induced by laser alignment processing in films thinner than 250 nm. Similar experiments were conducted using lenses with different depths of field; however, the alignment depth (the film thickness at which anisotropy occurred) remained unchanged at 250 nm (Supplementary Fig. 9). These findings suggest that the processing depth for laser alignment is independent of the lens characteristics or focal positioning. The 250-nm processing depth reported here is an estimate based on optical and electrical anisotropies; we note that this value should be further validated using cross-sectional imaging or depth-profiling spectroscopy^43^. These results demonstrate that the laser-aligned CNT film can function as a local polariser and polarisation imaging pixel in the THz range, and that the direction of electric current and heat flow can be controlled locally.

In this study, we developed an optical post-processing technology for controlling CNT nanostructures. By utilising the polarisation-dependent optical properties of CNTs, we achieved control over CNT alignment. We demonstrated the generalisability of laser alignment technology by changing various conditions, such as the synthesis method, substrate, and processing atmosphere of the CNTs, and revealed that the surface nematic order parameter *S* was 0.93. This indicates a high degree of alignment. Furthermore, we demonstrated the application of optical and electrical anisotropy to laser-aligned structures. The greatest advantage of this technique is its ability to control the nanostructure post-processing after film deposition. This enables localised control of the structure within the laser-irradiated area. This feature overcomes the integration limitations of previous techniques and holds promise for applications in division-of-focal-plane polarisation cameras in the THz-to-infrared range, as well as in electronic devices with locally controlled current and heat flow.

The optical post-processing reported here represents an emerging technology, and many aspects remain to be addressed. The main challenges associated with laser-aligned CNTs include achieving consistent morphological uniformity and packing density. In this investigation, aligned CNTs were produced by selectively removing extraneous CNTs from an initially random assembly, without any pretreatment. This removal process resulted in a packing density of ≈30 bundles per μm, which is substantially lower than the 200–1000 tubes per μm reported in prior research^22,44^. With respect to optical and electrical anisotropy, the polarisation absorbance ratio at a wavelength of 620 nm (2 eV) was measured at 2.5, compared to roughly 3 in earlier studies^22^. Similarly, electrical anisotropy was considerably less pronounced, exhibiting a ratio of 3.4 as opposed to 59 reported in ref. ^22^. However, it is important to clarify that the morphological uniformity and packing density achieved through laser alignment are contingent upon the initial CNT structure. Therefore, we propose that this limitation can be addressed by utilising pre-aligned films based on established techniques. For example, locally aligned CNTs with increased packing density should be obtainable by stacking wafer-scale alignment films with varied orientations and implementing our laser alignment technology. Thus, the packing density and uniformity achieved with our laser alignment technique are governed primarily by the material properties rather than the process itself. Integrating this technology with conventional alignment methods should considerably improve performance, offering mutual benefits to our approach and alternative technologies.

Another possible approach is to use this technology as a purification method to improve the performance of CNT transistors^45^. Laser irradiation may remove dispersants and structural defects of CNTs (Fig. 4). For instance, this technique could potentially improve subthreshold swing and hysteresis in CNT transistors via non-thermal dispersant removal. Furthermore, the fact of performing alignment engineering that reflects the polarisation characteristics of CNTs through non-thermal processing suggests the possibility of chirality-selective ablation, indicating the absorption wavelength of each chirality. This can lead to a new chirality engineering technology based on optical effects that fundamentally differ from conventional technologies^46–51^. Additionally, as this technique reflects the optical properties of the material, it could potentially be applied to other nanomaterials, including not only CNTs but also hetero-nanotubes and transition metal dichalcogenides. In other words, we believe that this optical post-processing method has great potential and will therefore contribute to advances across many areas of nanomaterial research.

## Methods

### Materials

We used a metallic–semiconducting mixed CNT dispersion (eDIPS EC-2.0 P) (Meijo Nano Carbon Co., Ltd) for the laser alignment experiment. The CNTs were synthesised using an enhanced direct-injection pyrolytic synthesis method^2^. The CNTs featured diameters of 2–3 nm and lengths extending several micrometres. The dispersant was an aqueous surfactant. Supplementary Fig. 10 illustrates the details of the sample preparation method. The CNT dispersion was vacuum filtered through a polyvinylidene fluoride (PVDF) membrane filter. The CNT film was then transferred onto a polyimide substrate. The CNT film thickness was adjusted to 25–1000 nm by varying the amount of dispersion. The actual CNT film thickness was evaluated using a confocal laser microscope (VK-X1000/1050, Keyence Corporation). In the alignment using different CNT synthesis (Supplementary Fig. 4), ZEONANO SG101 (Zeon Corporation) and Lamfil WPB-030 (Kusumoto Chemicals, Ltd.) were used in addition to eDIPS EC-2.0 P. In the alignment using different CNT dispersants (Supplementary Fig. 5), Lamfil WPB-030 (carboxymethyl cellulose), Lamfil WBC-040 (poly(acrylic acid)), and Lamfil WPO-253 (surfactant) purchased from Kusumoto Chemicals, Ltd. were used.

### Laser irradiation

The femtosecond-pulsed laser source was a CARBIDE CB5-6W (Light Conversion, UAB). The pulse width was 0.2–20 ps (tunable), wavelength was 1030 nm, and repetition rate was 1 MHz. The picosecond-pulsed laser source was an Olive-1064 (Huaray Precision Laser Co., Ltd). The pulse width was <10 ps, and the repetition rate was 1 MHz. The fundamental wavelengths were 1064 nm and its second harmonic (532 nm), generated using a non-linear optical crystal (LiB_3_O_5_). The nanosecond-pulsed laser source was a Cobolt Tor XS (Hübner Photonics) with a pulse width of 2–3 ns and a repetition rate of 1 kHz. The wavelength was 532 nm.

The laser energy (fluence) was adjusted using a half-wave plate and a polarising beam splitter. The collimated light was introduced into lenses with different magnifications (objective lens and f-Θ lens) and irradiated onto the CNTs. The objective lens was used in a focusing optical system combined with a two-axis automatic stage, while the f-Θ lens was used in a scanning optical system combined with a galvanometer mirror. The laser energy was measured by inserting a power metre between the lens and the CNTs. The fluence was calculated by dividing the measured energy by the spot diameter, which was estimated from the wavelength λ (532, 1030, and 1064 nm) and lens NA as 1.22× *λ*/NA, which is the approximate formula of the Airy disk. The fluence was calculated assuming a uniform energy distribution within the spot plane. This assumption explains why the fluence threshold changes in the experimental results when using different lenses. The polarisation of the laser was manipulated using half-wave and quarter-wave plates. Laser processing was performed under ambient air, argon, and vacuum conditions.

### Characterisation

An ultra-high-resolution Schottky SEM, SU8700 (Hitachi High-Tech Corporation), was used for SEM imaging, and a confocal Raman spectrometer MicroRAM-300 (Lambda Vision Inc.) was used for Raman measurements. The THz spectra were measured using THz time-domain spectroscopy, TAS7500 (Advantest Corporation). The optical system was configured for transmission measurements, and the entire setup, including the sample and optical path, was purged with dry nitrogen to prevent undesired THz absorption by water vapour. The optical anisotropy ratio was calculated by dividing the absorptance parallel to the CNT alignment direction by the absorptance perpendicular to it. This study used the absorptance at 2 THz as a representative value. The electrical properties of the aligned CNTs were measured using the four-point method, with electrodes attached to the CNT film.

AFM measurement was conducted in non-contact mode using a commercially available system (Vista one, Molecular Vista). The second-resonance frequency of the cantilever (1.6 kHz) was used for detection. The tip oscillation amplitude was fixed to 1 nm (set point: 85 %) and 2.5 nm (set point: 68%) for pristine CNTs and laser-aligned CNTs, respectively. The nominal force constant of the tip is 42 N m^−1^ and the scanning rate of the tip was 2 μm s^−1^.

TEM imaging was performed at 60 kV on TripleC#1 JEOL 2100 F microscope, fitted with a cold field-emission gun and dodecapole-based aberration correctors. The beam current density used for imaging was ≈5 × 10^6^ e nm^−2^ s^−1^.

Colormaps for orientation and distributions of CNTs (Fig. 5c, d) were obtained from SEM images using OrientationJ. An ImageJ plugin (ImageJ v1.54)^52,53^. Local orientations were estimated at each pixel from the gradient structure tensor. Image gradients were calculated using cubic spline gradient estimation, and the gradient product terms were locally averaged with a Gaussian analysis window with a standard deviation of 1 pixel. The resulting pixelwise orientation values were used to calculate orientation-angle distributions.

Polarised Raman and transmission spectroscopy (Fig. 5h, i) were performed to estimate nematic order parameter *S*. A 100-nm-thick sample on a sapphire substrate was laser-aligned in a vacuum using a 10× objective lens at a fluence of 150 mJ cm^−2^. Polarised Raman spectroscopy was performed in the backscattering configuration using a home-made optical setup^54^. A 20× objective lens with NA = 0.42 was used for excitation and detection. The light source was a linear-polarised 532-nm CW laser with a power of 200 μW, and the exposure time was 100 s. The analyser to resolve the polarisation of the scattered photons was set behind an edge filter with a cut-off wavelength of 532 nm, and the scattered photons were detected using a spectrometer (Princeton Instruments, Acton SP2500) equipped with a thermoelectrically cooled charge-coupled device camera (Teledyne, ProEM-1600 × 200). The polarisation-dependent detection sensitivity was corrected using unpolarised luminescent light. Transmission spectroscopy was performed using a home-made optical setup^55,56^. Broadband light from a halogen lamp passing through an optical fibre was collimated using a parabolic mirror and converted from unpolarised to linearly polarised light. The light was irradiated with normal incidence on the sample. The light transmitted through an optical fibre was detected with a detector (YIXIST, YSM-8101-02-18-16S03L02F06G16). For both experiments, the light polarisation angle against the sample orientation was set by rotating the sample. The nematic order parameter *S* was estimated according to ref. ^42^.

### Statistics and reproducibility

SEM and TEM images of Figs. 1c–k; 2a–h; 3b–g; 4a–k; 5a, b, d, e, g were measured from at least three independent experiments.

### Reporting summary

Further information on research design is available in the Nature Portfolio Reporting Summary linked to this article.

## Supplementary information

Supplementary Information

Description of Additional Supplementary Files

Supplementary Data 1

Reporting Summary

Transparent Peer Review file

## Source data

Source Data

## Data Availability

The data that support the findings of this study are available from the corresponding authors upon request. Unprocessed raw data are provided as Supplementary Data 1. Source data are provided with this paper.
