# Supplementary material for: Localized polarization-guided alignment control in carbon nanotube films via femtosecond-pulse optical post-processing
Source: Nat Commun. 2026 Aug 1;17:7923. doi: 10.1038/s41467-026-76305-w (PMC13448317; doi:10.1038/s41467-026-76305-w)
Supplement: Supplementary file 1 [file 41467_2026_76305_MOESM1_ESM.pdf]

Supplementary Information

Localized polarization-guided alignment control in carbon nanotube films via femtosecond-pulse optical post-processing

Daichi Suzuki[1,*], Yung-Chang Lin[2], Yuma Takida[3], Hideaki Nakajima[4], Kaori Fujii[4], Takaaki Abe[5], Naoko Kurihira[5], Teppei Araki[5], Masanori Koshino[2], Toshiya Okazaki[4], Hiroaki Minamide[3], Nao Terasaki[1], and Taishi Nishihara[6,7,*]

[1]*Sensing Technology Research Institute, National Institute of Advanced Industrial Science and Technology (AIST), Saga, Japan.*

[2]*Research Institute of Core Technology for Materials Innovation, National Institute of Advanced Industrial Science and Technology (AIST), Tsukuba, Japan.*

[3]*RIKEN Center for Advanced Photonics, RIKEN, Miyagi, Japan.*

[4]*Nanocarbon Material Research Institute, National Institute of Advanced Industrial Science and Technology (AIST), Ibaraki, Japan.*

[5]*SANKEN (The Institute of Scientific and Industrial Research), The University of Osaka, Osaka, Japan*

[6]*Department of Physics, Tokyo University of Science, Tokyo, Japan.*

[7]*Research Institute for Science and Technology, Tokyo University of Science, Tokyo, Japan*

* Corresponding author. Tel: +81-942-81-3620. E-mail: daichi.suzuki@aist.go.jp

* Corresponding author. Tel: +81-3-3260-4271. E-mail: nishitai@rs.tus.ac.jp

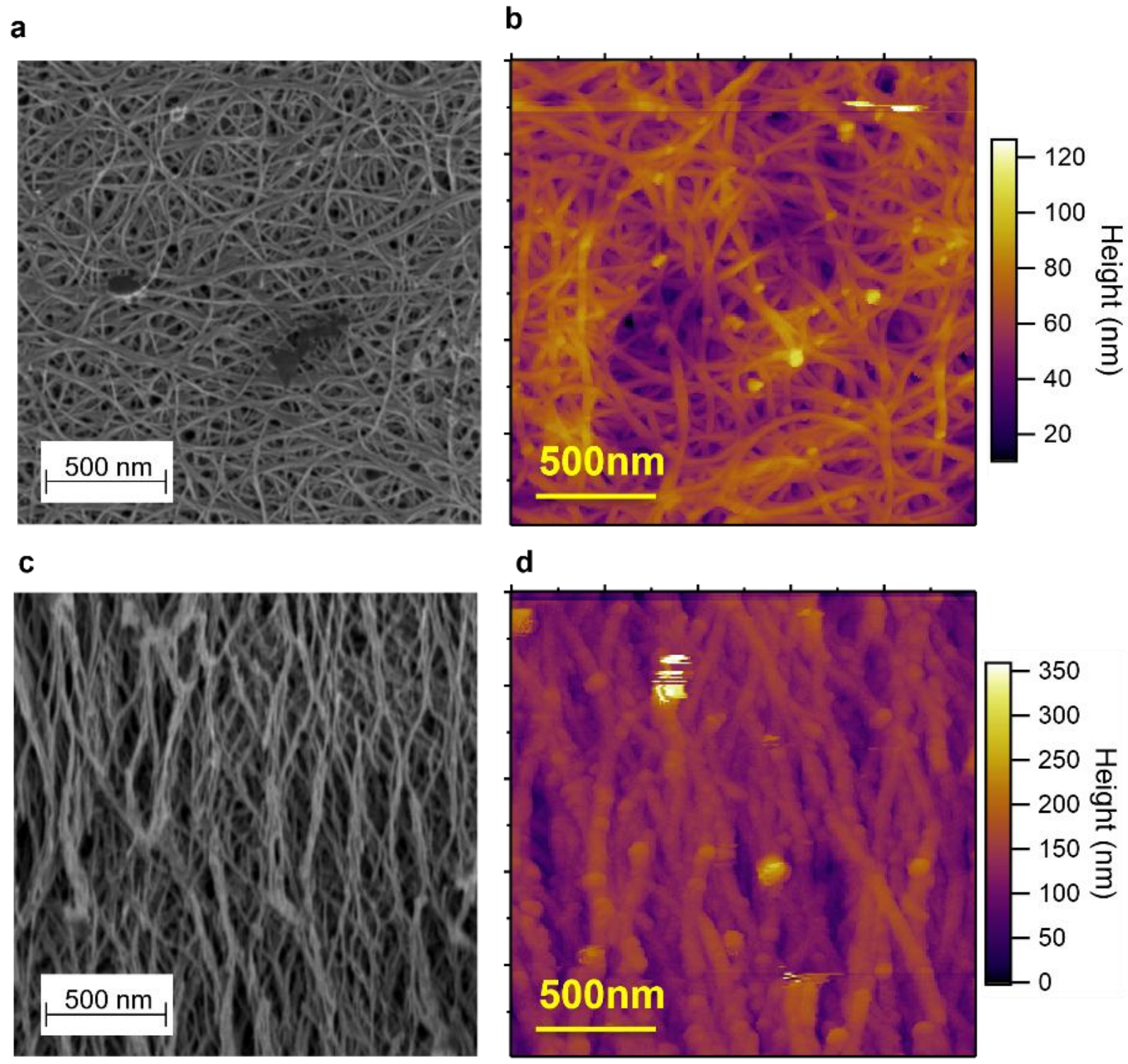

Supplementary Fig. 1| Atomic force microscopy (AFM) images of the laser-aligned carbon nanotubes (CNTs). a, b, (a) Scanning electron microscopy (SEM) image and (b) AFM image of pristine CNTs. **c, d,** (c) SEM image and (d) AFM image of the laser-aligned CNTs.

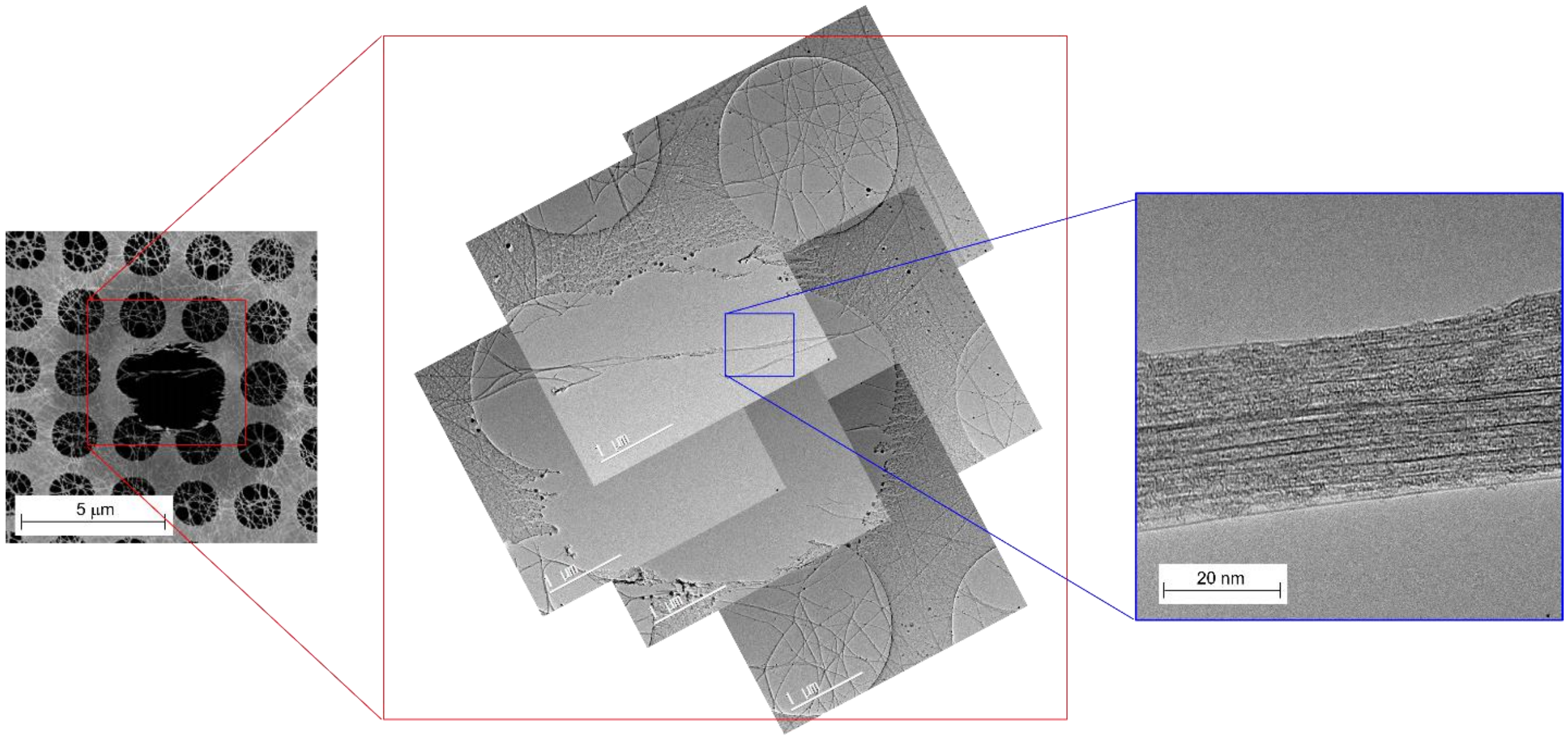

Supplementary Fig. 2| Scanning electron microscopy and transmission electron microscopy images of the laser-aligned carbon nanotubes.

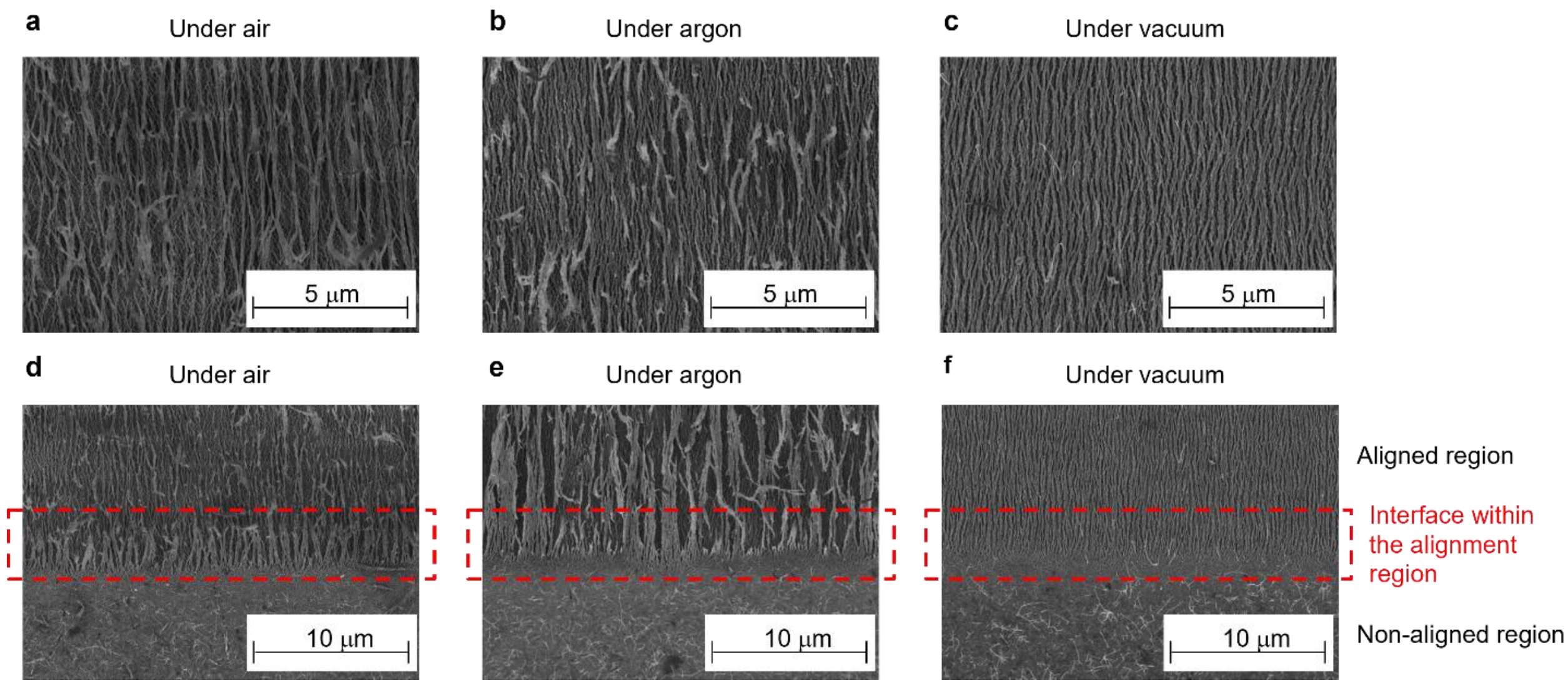

Supplementary Fig. 3| Comparative results obtained under different atmospheres. a–f, (a–c) Magnified images and (d–f) interface images of the laser-aligned carbon nanotubes performed under (a, d) air, (b, e) argon, and (c, f) vacuum.

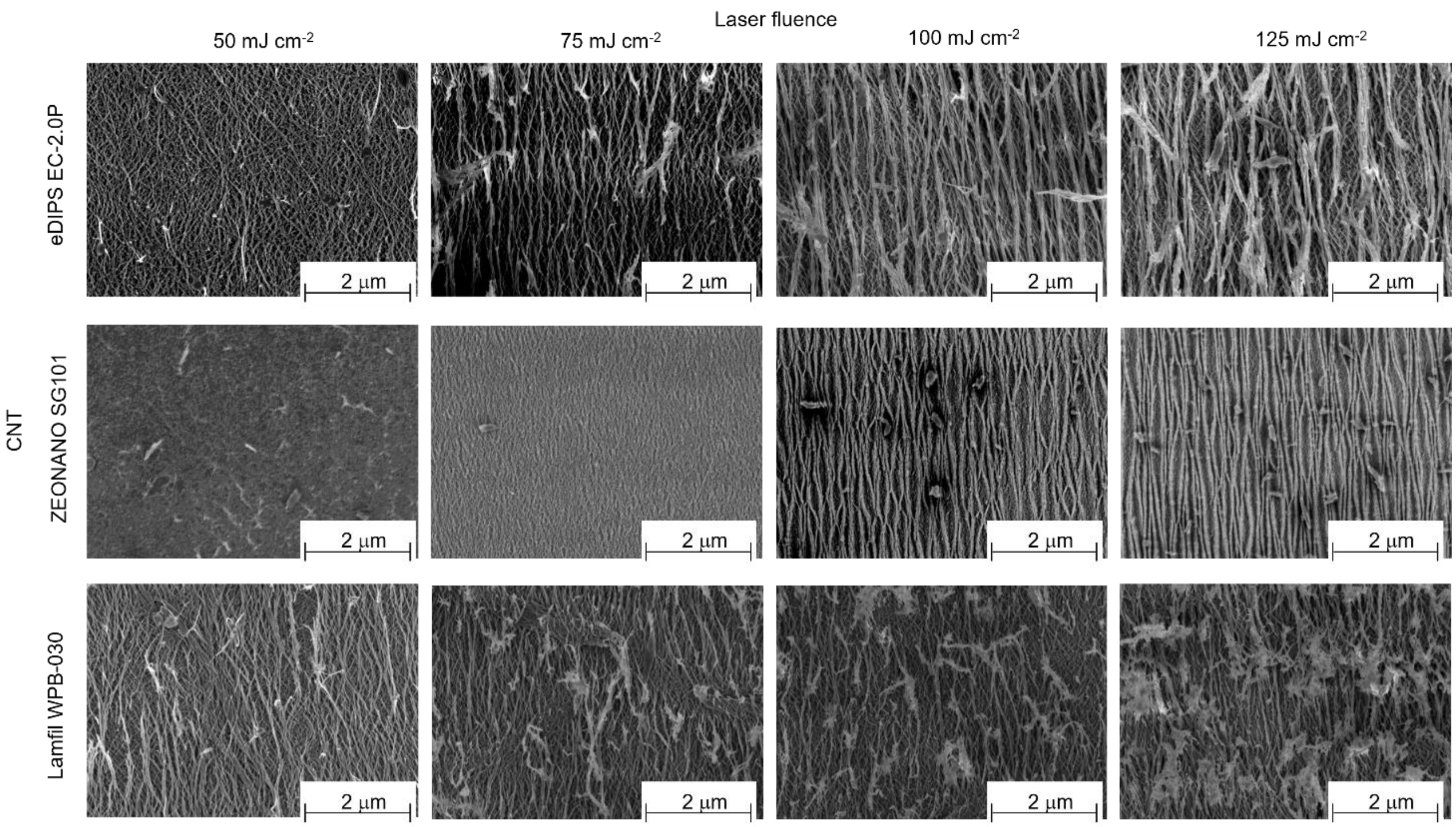

Supplementary Fig. 4| Comparative results obtained using different carbon nanotube syntheses.

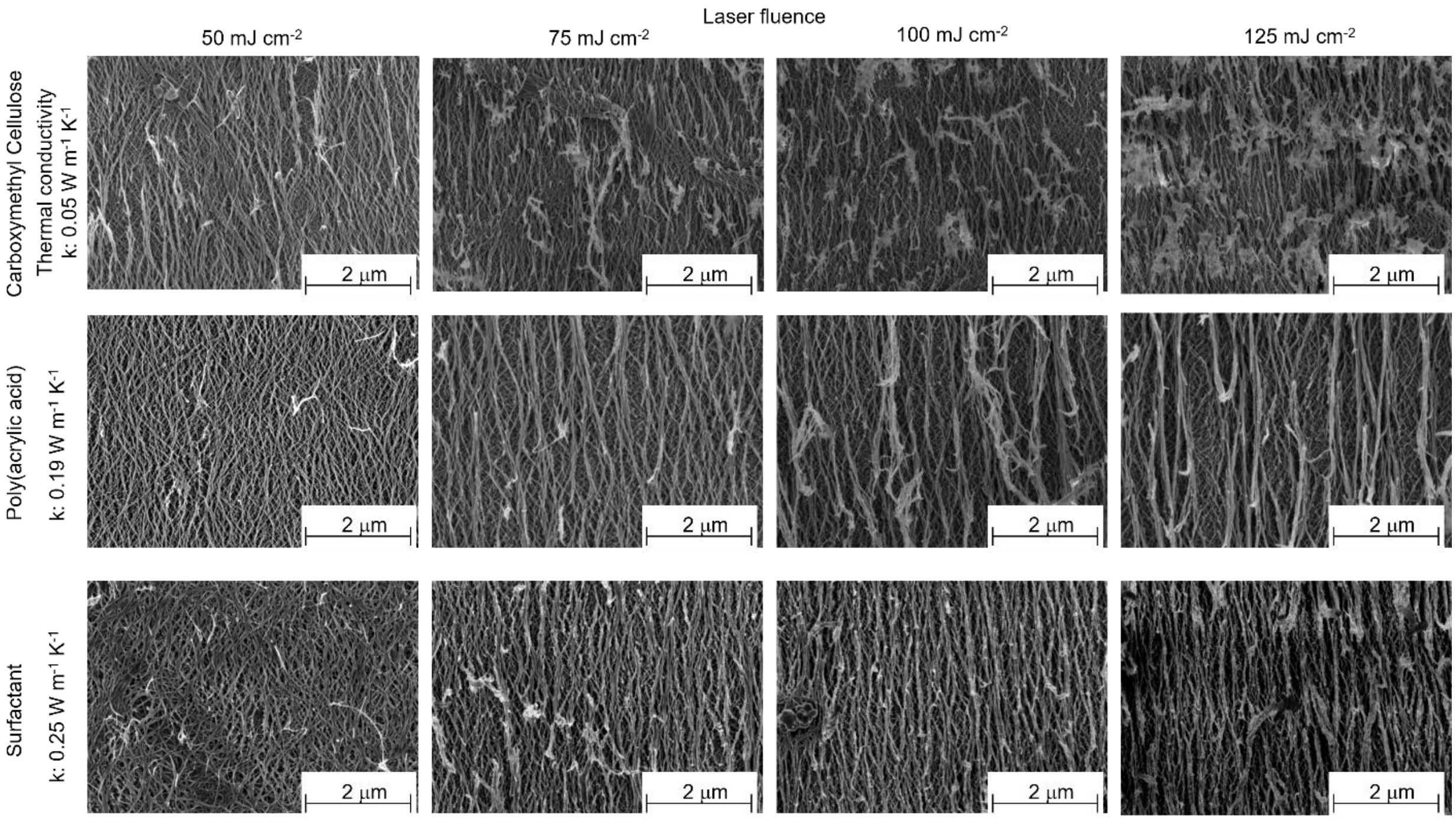

Supplementary Fig. 5| Comparative results obtained using different carbon nanotube dispersants.

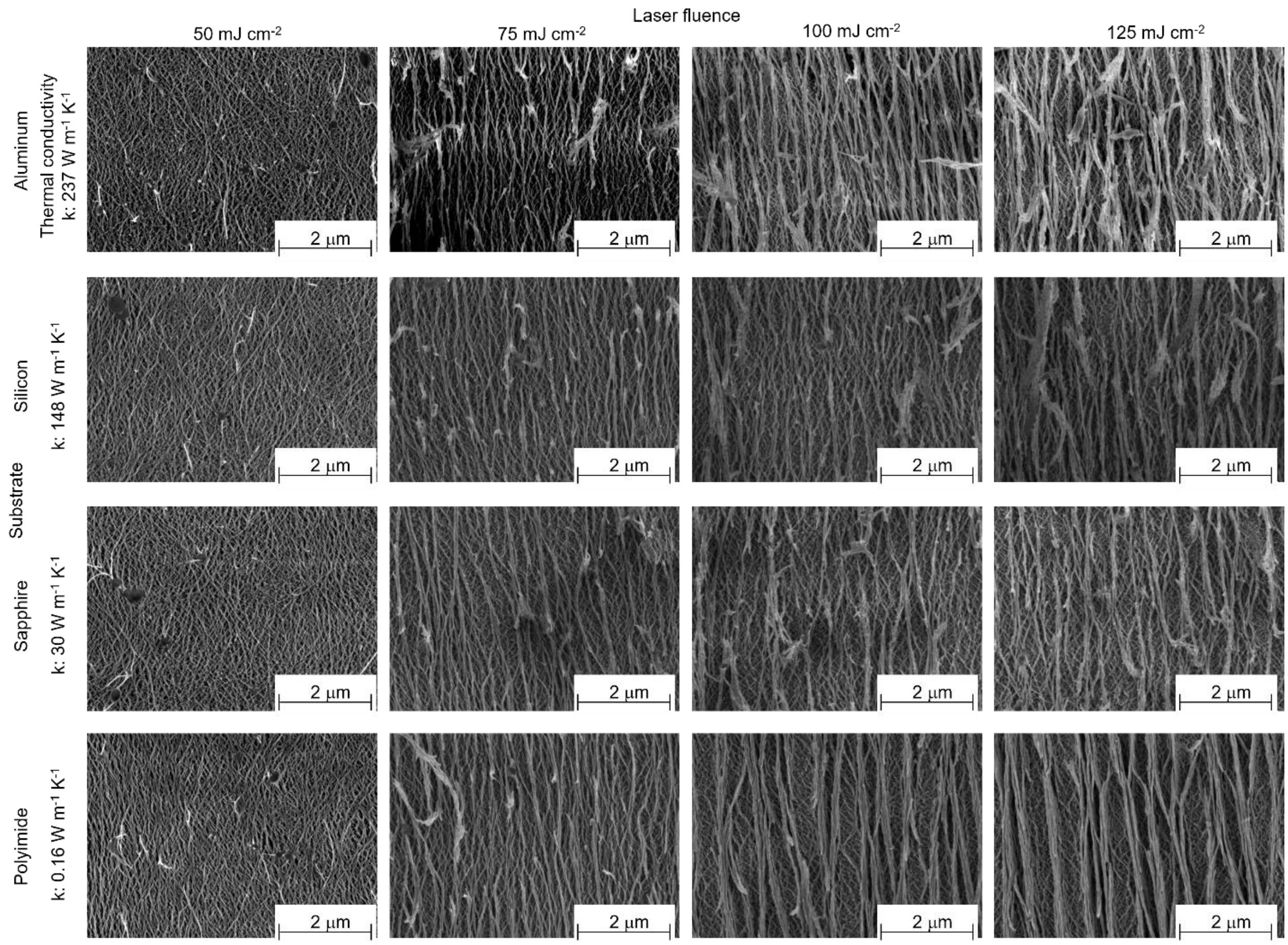

Supplementary Fig. 6| Comparative results under different substrates.

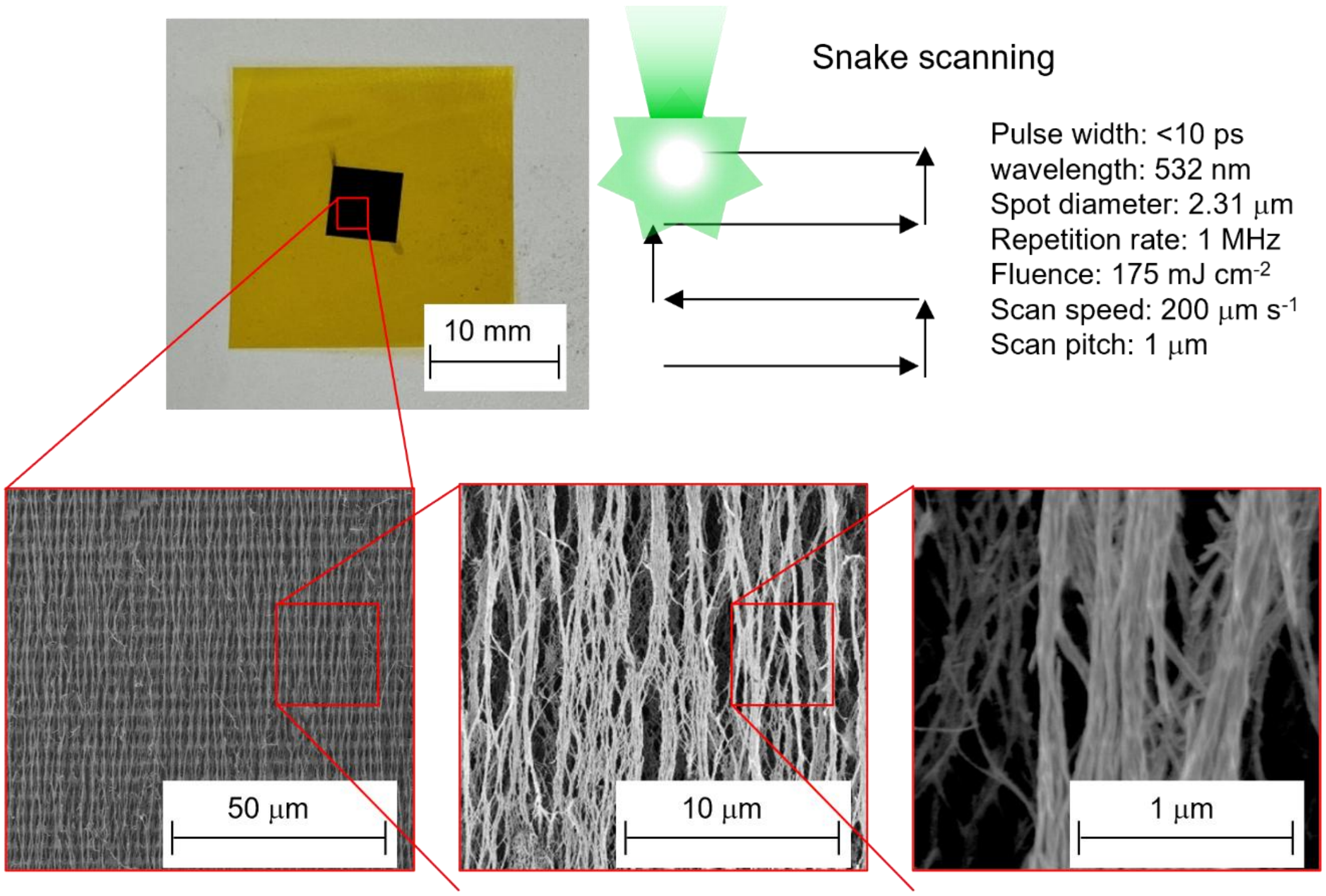

Supplementary Fig. 7| Centimetre-scale aligned carbon nanotube film achieved by snake scanning.

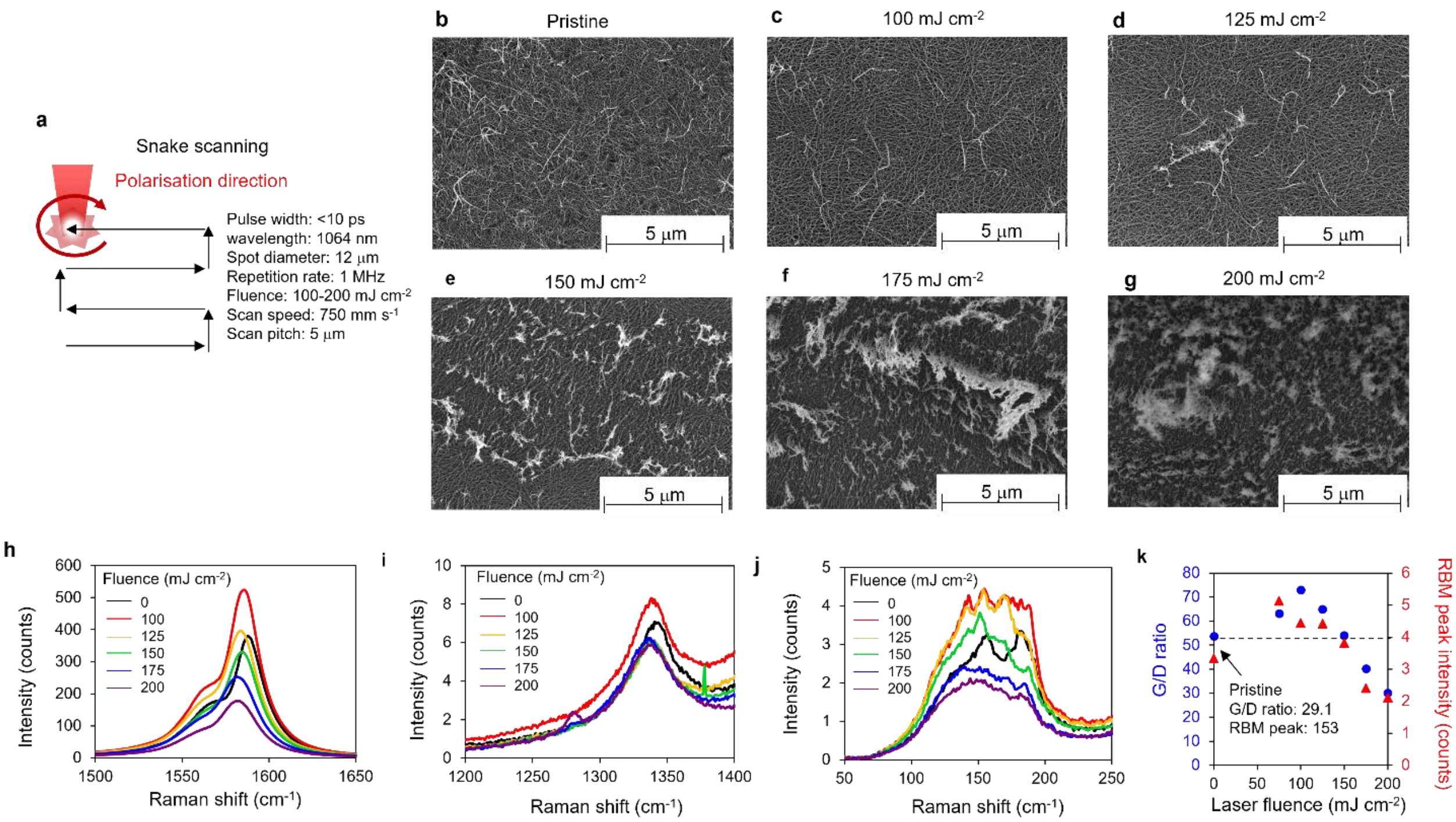

Supplementary Fig. 8| Raman spectroscopy of laser-unaligned carbon nanotubes (CNTs). a, Schematics of the laser irradiation condition. **b–g,** Scanning electron microscopy images of CNTs irradiated with laser fluence of (b) 0, (c) 100, (d) 125, (e) 150, (f) 175, and (g) 200 mJ cm^{-2}. **h–j,** Enlarged Raman spectra of the (h) G band, (i) D band, and (j) radial breathing mode (RBM) peak. **k,** Change in G/D ratio and RBM peak after laser irradiation for each fluence. Source data are provided as a Source Data file.

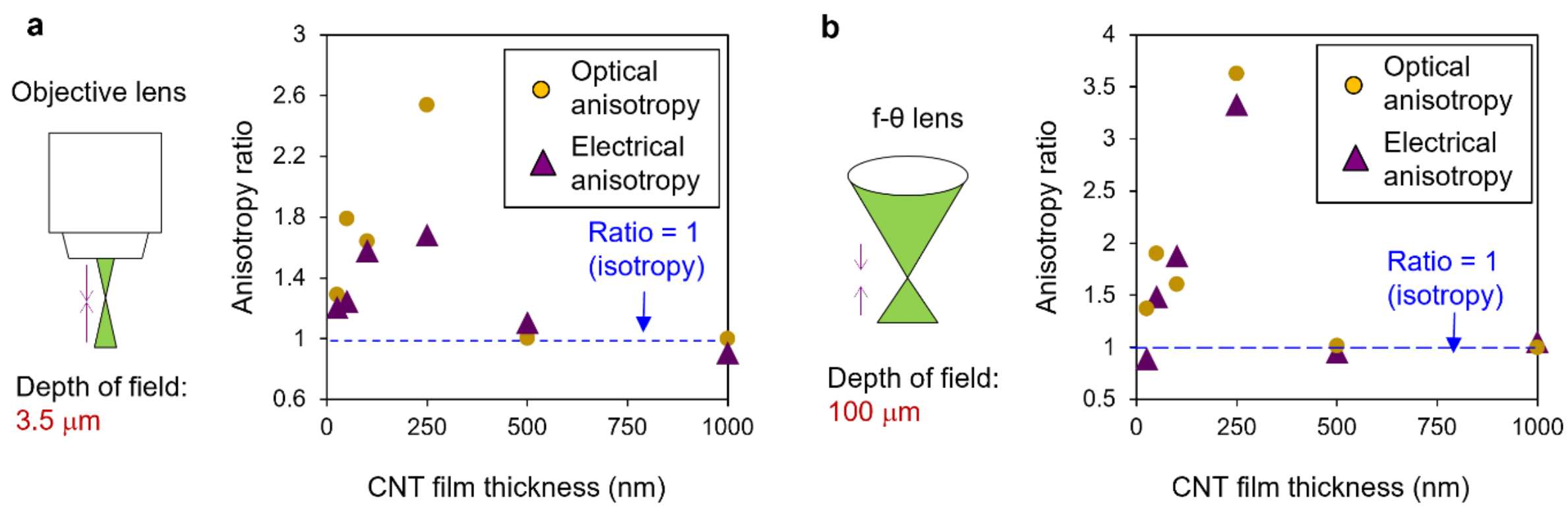

Supplementary Fig. 9| Optical and electrical anisotropy of the laser-aligned carbon nanotube (CNT) film irradiated with different lenses with varying depths of field. a, Optical and electrical anisotropy of laser-aligned CNTs via objective lens. **b,** Optical and electrical anisotropy of laser-aligned CNTs via f-Θ lens. Source data are provided as a Source Data file.

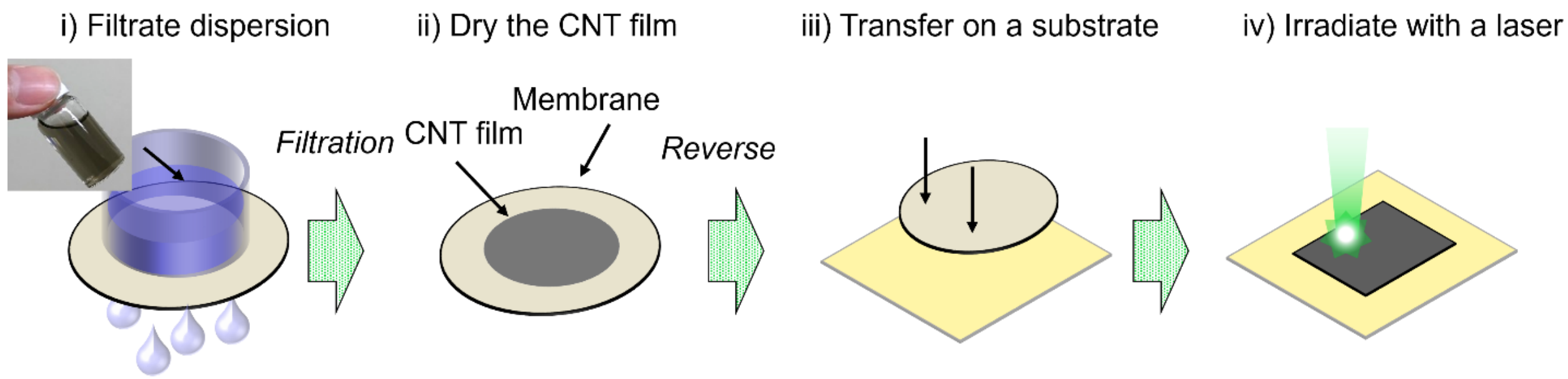

Supplementary Fig. 10| Schematic of sample preparation.

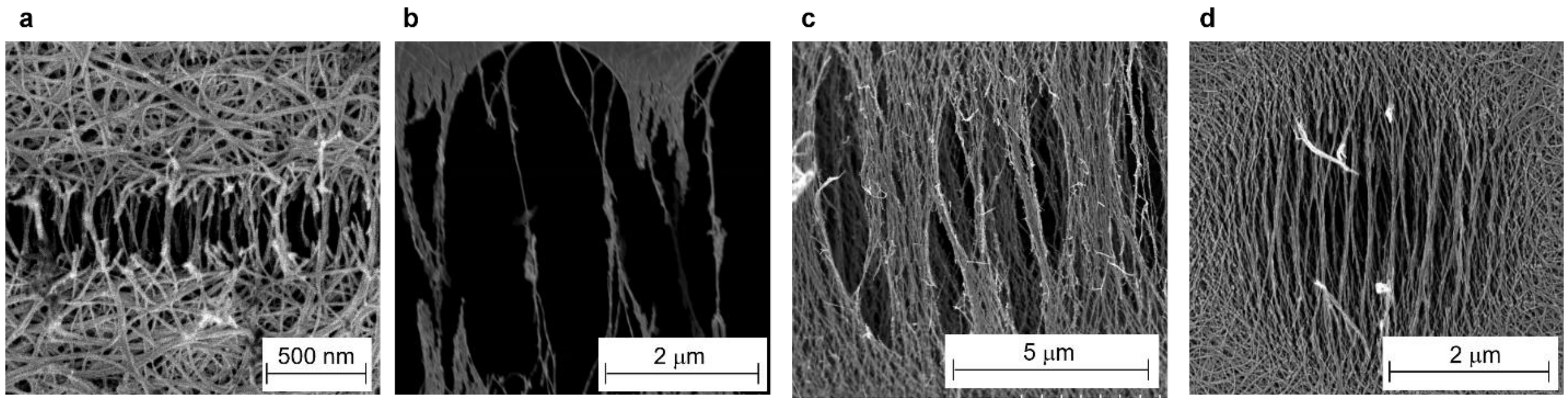

Supplementary Fig. 11| Alignment structure when the density and spot diameter of carbon nanotubes (CNTs) were changed. a, Condition where the spot diameter is smaller than the CNT length. **b,** Conditions with low CNT density. **c,** Condition where the spot diameter is larger than the CNT length. **d,** Conditions with high CNT density.

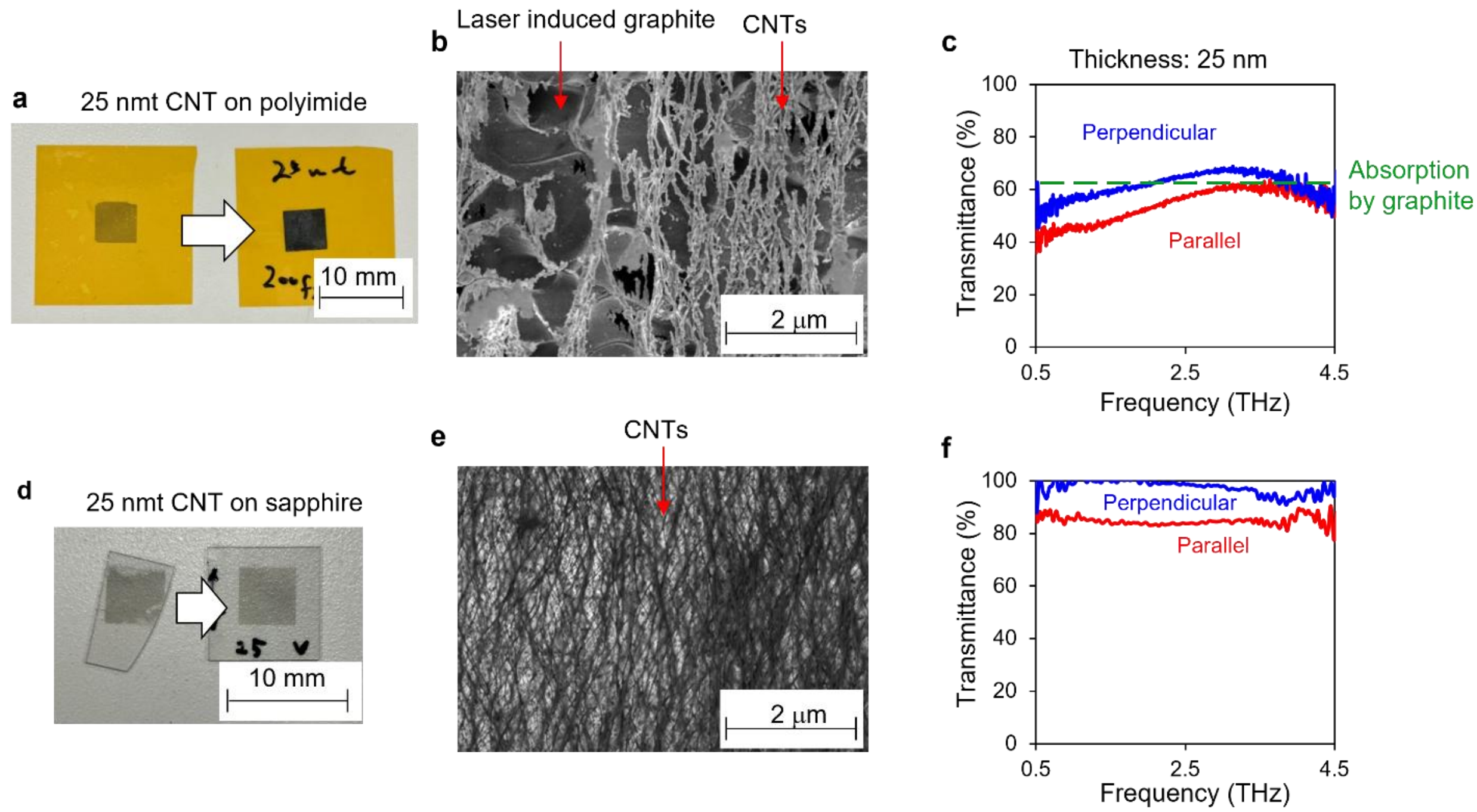

Supplementary Fig. 12| Effects of substrate carbonization. a–c, Laser-aligned carbon nanotubes (CNTs) on the polyimide substrate. (a) photographic image before and after laser irradiation. (b) Scanning electron microscopy (SEM) image of the CNTs and laser-induced graphite. (c) THz transmission spectra. **d–f**, Laser-aligned CNTs on the sapphire substrate. (d) photographic image before and after laser irradiation. (e) SEM image of the CNTs. (f) THz transmission spectra. Source data are provided as a Source Data file.

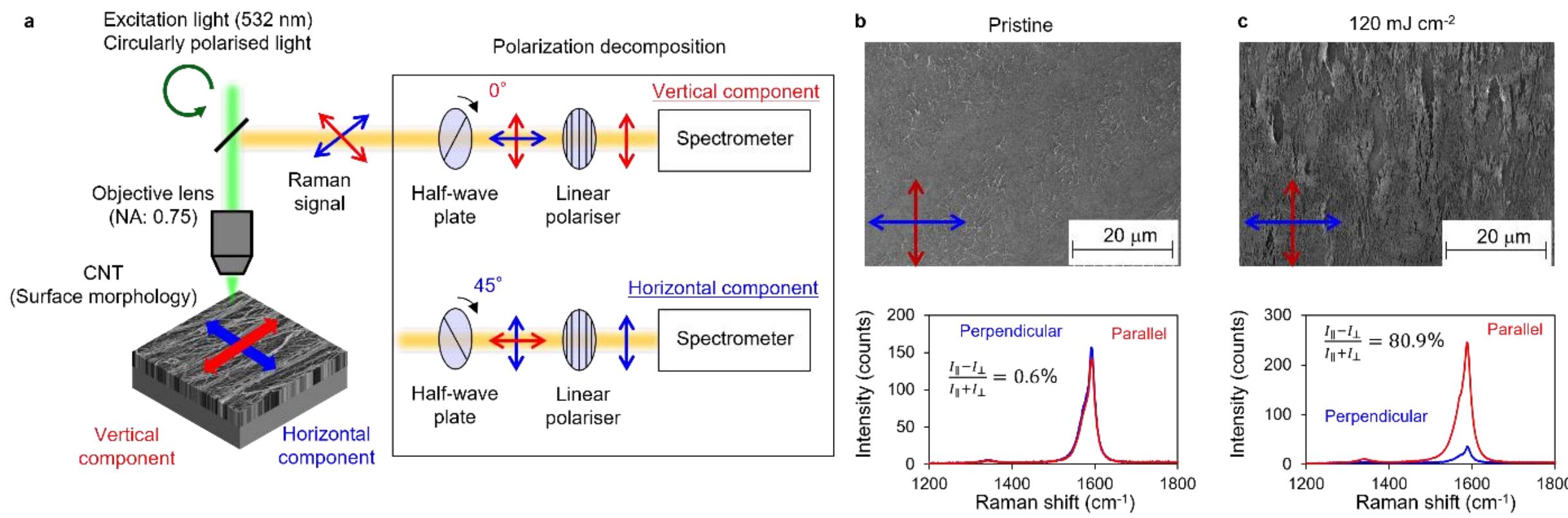

Supplementary Fig. 13| Raman spectroscopy of pristine and laser-aligned carbon nanotubes (CNTs). **a,** Schematic diagrams of the polarisation-resolved Raman spectroscopy. The resulting Raman signal from the sample was passed through a half-wave plate and a linear polariser to select its vertically and horizontally polarised components. **b, c,** Scanning electron microscopy images and polarisation-resolved Raman spectra of CNTs (b) without and (c) with laser irradiation. Source data are provided as a Source Data file.

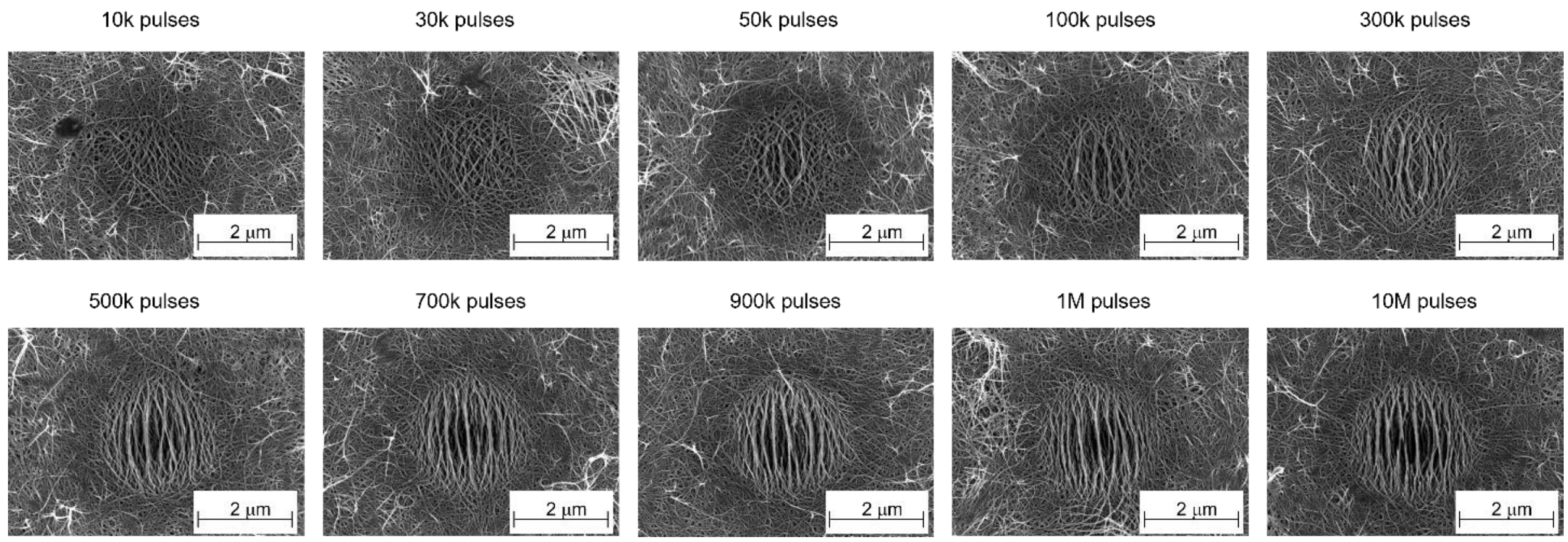

Supplementary Fig. 14| Dependence on the number of irradiation pulses. The laser conditions were as follows: wavelength, 1030 nm; pulse width, 200 fs; fluence, 40 mJ cm^{-2}; and polarisation direction, horizontal. As the number of irradiation pulses increased, carbon nanotubes (CNTs) parallel to the laser polarisation direction were progressively removed, leading to the gradual formation of an aligned structure. At approximately 500k pulses, horizontally oriented CNTs at the surface were removed, resulting in the formation of vertically aligned CNTs. Even when the number of pulses was further increased to 10M, the surface alignment structure was maintained, while deeper material removal proceeded. These results suggest that CNTs oriented perpendicular to the laser polarisation direction undergo negligible damage under the present irradiation conditions.
