# Supplementary material for: Localized polarization-guided alignment control in carbon nanotube films via femtosecond-pulse optical post-processing
Source: Nat Commun. 2026 Aug 1;17:7923. doi: 10.1038/s41467-026-76305-w (PMC13448317; doi:10.1038/s41467-026-76305-w)
Supplement: Supplementary file 2 [file 41467_2026_76305_MOESM2_ESM.pdf]

Description of Additional Supplementary Files

File Name: Supplementary Data 1

Description: Unprocessed raw data
