# Supplementary material for: Localized polarization-guided alignment control in carbon nanotube films via femtosecond-pulse optical post-processing
Source: Nat Commun. 2026 Aug 1;17:7923. doi: 10.1038/s41467-026-76305-w (PMC13448317; doi:10.1038/s41467-026-76305-w)
Supplement: Supplementary file 4 [file 41467_2026_76305_MOESM5_ESM.pdf]

nature portfolio

Peer Review File

Localized polarization-guided alignment control in carbon nanotube films via femtosecond-pulse optical post-processing

Corresponding Author: Dr Daichi Suzuki

This file contains all editorial decision letters in order by version, followed by all author rebuttals in order by version.

Attachments originally included by the reviewers as part of their assessment can be found at the end of this file.

Version 0:

Reviewer comments:

Reviewer #1

(Remarks to the Author)
In this work, the authors reported the short-pulse laser irradiation induced local alignment and chirality control of carbon nanotube films. The influence of laser pulse-width, pulse energy and film thickness was systematically studied and the non-thermal photon-induced destruction of proposed for the local alignment. The polarized Raman scattering spectroscopy and THz time-domain spectroscopy were used to characterize the anisotropic ration of the local alignment of carbon nanotubes. Although this work was comprehensive and the local alignment was evident, the reviewer do not recommend publication of this work in Nature Communications at this stage due to the following reasons.
1. The authors claimed chirality control of the aligned carbon nanotube films, however they only showed a very preliminary result of carbon nanotube film with two chiral indices. This is barely a perspective rather than practical chirality control. If chirality-specified CNTs can be prepared, why not simply prepare aligned CNTs with single chirality?
2. The authors proposed non-thermal photon-induced destruction of carbon nanotube film for local alignment for ps laser irradiation, and photon induced thermal destruction for nanosecond and cw lasers. The key issue for successful alignment would be the thermal diffusion after laser absorption. For ps- or fs-pulsed laser irradiation, the absorbed photons would excite electrons and the excited electrons would still relax and generate heat via non-irradiative pathways. If it is non-thermal process, what would be the driving force to remove carbon nanotubes?
3. If thermal-related effect is the key issue, any local environment with different thermal conductivity, including the substrate and inclusion of BNNT, would affect the results. Have the authors considered and tested such effects?

Minor revision suggestions
1. Page 4, “absorbing parallel light and transmitting light perpendicular to their length”, it is “light polarization”, not “light”, and “long axis” not “length”.
2. Please give the information of laser wavelength, pulse-width and energy at the first place when introducing fig1 in page 4.
3. How the spot diameter of laser spot was estimated from lens NA?
4. Page 6, “we discuss the physical properties of laser-aligned CNT films using SEM observation and Raman spectroscopy”. It is the “quality” of CNT films can be given by SEM and Raman spectroscopy.
5. In Fig 5, the authors showed BNNT was used, and they discussed “to improve compatibility with the CNT solutions” in the Method section. More detailed discussion should be given with appropriate references for why BNNT was used, and would BNNT affect the laser induced destruction or not.

Reviewer #2

(Remarks to the Author)
The manuscript by Suzuki et al. presents interesting and surprising results. It is very surprising that polarized laser pulses are able to so selectively remove one orientation of CNTs but not the other, from randomly aligned films of CNTs. The data strongly support this observation, at least in very specific types of CNT films. One large drawback of this work is that it is not

clear how generalizable this approach might be. For example, the approach does not seem to work very well for the conjugated polymer wrapped CNTs in Fig. 5 that have very well defined (n,m) CNTs. In this Fig. 5, CNTs in resonance with the laser are only marginally more removed than ones not in resonance, suggesting a lack of selectively. The approach does work for the mystery CNTs described in Figs. 1-4; however, those CNTs and the composition of those CNT films are not well described by the paper. It is therefore impossible to judge the generalizability and impact that this manuscript might have. The following major revisions would be needed before the paper could be re-considered.

WHY DO THEY SAY NON-THERMAL? “ultrashort-pulsed lasers (via non-thermal processing)

“For ultrashort pulses such as picosecond or femtosecond pulses, the pulse width is shorter than the intraband thermalization time of carriers (a few picoseconds)34,35; therefore, processing occurs before significant thermal diffusion, resulting in a non-thermal photon-induced destruction phenomenon.” 100 ps is pretty fast must faster than the intraband relaxation time.

They mention ablation. How do they know?

The “wavelength-selective, chirality-dependent removal in a bichiral CNT film” is not very good. Barely any selectivity.

Major:

1) What are the diameter, length, surfactant or polymer wrapper of the CNTs used in Fig. 1-4. What are their absorption spectra?

2) The mechanism is attributed to be “ablation” and “non-thermal” at the same time. Isn’t ablation due to thermal heating of the carbon from the laser? Why is this called non-thermal?

3) What is the removal mechanism? Heat induced oxidation? Amorphitization/vaporization? These should be differentiated by repeating experiments in vacuum or an inert environment.

4) The ability to selectively heat up one chirality of CNT or one orientation of CNT and thereby remove it, hinges on poor thermal conductance of CNT-CNT and CNT-substrate interfaces. The selectivity seems to be much better in Figs. 1-4 than Fig. 5. Why? What is the nature of CNT-CNT interfaces of the films in Figs. 1-4, is there surfactant or polymer in between the CNTs to help decouple CNTs oriented in one direction from CNTs oriented in the orthogonal direction?

5) 10 ps is plenty of time for excited electron-hole pairs in CNTs to relax and give off phonons. In unsorted nanotube films, this can occur in 100’s of fs, much faster than the 10 ps pulse width.

Minor:

6) What the authors term “alignment degree” is more often called the “nematic order parameter” or just “order parameter”?

7) Why are the BNNTs used in the 6,5/7,5 films?

Reviewer #3

(Remarks to the Author)

Please see the attached PDF.

Version 1:

Reviewer comments:

Reviewer #1

(Remarks to the Author)
The revised manuscript is much improved accoording to the reviewers' comments. The authors answered my questions and added corresponding discussions with supplemented data. The revised manuscript can be recommended for publication in Nature Communications.

Reviewer #2

(Remarks to the Author)
The authors have conducted a lot of additional experiments that are quite helpful especially the experiments showing there is no substrate effect, that alignment still occurs independent of atmosphere, and especially the experiments that show the pulse duration effect. The removal of the chirality selectivity (which were the least compelling data) and the addition of the above data all significantly improve the paper. The paper also now more clearly communicates the idea that the authors have regarding the mechanism. They believe the mechanism is that the hot electrons (or excitons) cause the ablation of the

nanotubes before the energy is converted into lattice vibrations. They argue the pulse duration experiments show this. There is one big problem still remaining however which is that it is known that exciton-phonon interactions in carbon nanotubes are very strong and can be very fast (as indicated in my previous review). The authors cite that in other materials these interactions are many ps in timescale. However in nanotubes they are faster. The authors cite 2 papers showing the scattering rate is multiple ps in nanotubes. However, I do not think those papers are being cited correctly and other papers that cite 100's of fs or faster scattering times are not cited. For example, the authors cite Ref. 37 "Dyatlova, O. A. et al. Ultrafast relaxation dynamics via acoustic phonons in carbon nanotubes. Nano Lett. 12, 2249–2253 (2012)." But if you read this paper Ref. 37 you will find the following sentence

"The optically excited carriers injected into the upper subband reach the energetically lower subband via Coulomb- and optical-phonon-induced inter- and intrasubband scattering processes, which all occur on a femtosecond time scale15,17 and are beyond the resolution of our experiment." Since the authors' laser is at energies much higher than the lowest subband of the nanotubes being used, there should be a very fast emission of optical phonons in the fs timescale. The paper by Dyatlova then goes on to discuss that after this significant and fast optical photon emission there is also a slower acoustic phonon scattering process that is slower (longer than ps). It does not seem like Dyatlova is being cited correctly. Furthermore, this paper "Intersubband Exciton Relaxation Dynamics in Single-Walled Carbon Nanotubes June 2005 Physical Review Letters 94(20):207401 DOI:10.1103/PhysRevLett.94.207401" and other papers in literature also indicate sub-100 fs relaxation to band edge via emission of phonons.

1) The authors need to more carefully examine and discuss in the paper the literature characterizing the ultrafast emission of phonons in carbon nanotubes after optical excitation.
2) Reconsider the mechanism (since the 2 ps pulses still show alignment but are much longer in duration than the sub-100 fs emission of phonons expected from previous experiments in literature.

3) The nanotubes parallel to the polarization are effectively removed in many of the cases in the paper leaving nanotubes perpendicular to the polarization. However, are nanotubes perpendicular also removed to some extend? Please quantify. For example, if a prealigned nanotube film were used as the starting point and the authors technique was used to remove a few misaligned nanotubes then would the aligned nanotubes decrease in packing density as a result (which would be undesirable).

Reviewer #3

(Remarks to the Author)
I thank the authors for their thorough and constructive response to my comments. The revised manuscript has been substantially improved and strengthened.

In particular, I appreciate that the authors carefully addressed my concerns regarding the framing of the work relative to existing alignment techniques, the terminology associated with chirality control, benchmarking against the state of the art, the interpretation of SEM images as CNT bundle structures rather than individual nanotubes, and the characterization of alignment using additional quantitative methods.

I also appreciate the additional characterization that was performed in response to my comments, including polarized visible spectroscopy, image-analysis-based determination of the nematic order parameter, TEM characterization, polarized Raman measurements using the VV, VH, and HH configurations, AFM characterization, and the expanded discussion of packing density, alignment quality, and comparison with existing CNT alignment approaches.

Several of the newly added results were particularly interesting. The observation that processing under vacuum produces noticeably cleaner alignment structures than processing in air or argon provides an intriguing insight into the role of the environment during laser alignment. I also found the additional D/G ratio analysis and TEM observations compelling, particularly the evidence suggesting removal of residual dispersant and preferential elimination of defective nanotubes. These experiments substantially strengthened the discussion of the processing mechanism and provided valuable insight beyond the original submission.

The removal of the preliminary chirality-selective ablation claims and the corresponding revision of the title significantly improved the focus and clarity of the manuscript.

I also found the demonstrations of more complex alignment geometries, including radial and multi-orientation structures, particularly interesting from the perspective of future THz photonic devices and polarization-sensitive architectures. Such structures may also provide an attractive platform for future studies of anisotropic optical and electronic phenomena.

Overall, I believe the authors have satisfactorily addressed my concerns and substantially improved the manuscript. I support publication of the manuscript in its present form and recommend acceptance.

Version 2:

Reviewer comments:

Reviewer #2

(Remarks to the Author)
The new, more nuanced hypothesis about mechanism is now much better. I recommend for publication.

Open Access This Peer Review File is licensed under a Creative Commons Attribution 4.0 International License, which permits use, sharing, adaptation, distribution and reproduction in any medium or format, as long as you give appropriate credit to the original author(s) and the source, provide a link to the Creative Commons license, and indicate if changes were made.
In cases where reviewers are anonymous, credit should be given to 'Anonymous Referee' and the source.
The images or other third party material in this Peer Review File are included in the article's Creative Commons license, unless indicated otherwise in a credit line to the material. If material is not included in the article's Creative Commons license and your intended use is not permitted by statutory regulation or exceeds the permitted use, you will need to obtain permission directly from the copyright holder.
To view a copy of this license, visit https://creativecommons.org/licenses/by/4.0/

Responses to Reviewer's Comments

Thank you for giving me the opportunity to submit a revised draft of my manuscript titled **Ultrashort-pulse optical post-processing for local alignment control in carbon nanotube films** to *Nature Communications*. We appreciate the time and effort that you and the reviewers have dedicated to providing your valuable feedback on this manuscript. We are grateful to the reviewers for their insightful comments on our paper. We have been able to incorporate changes to reflect most of the suggestions provided by the reviewers. We have highlighted the changes within the manuscript.

Here is a point-by-point response to the reviewers' comments and concerns.

The following figure shows an overview of the revision list.

The subsequent page contains detailed responses to the reviewer's comments.

Summary of Revisions

1. Conducted follow-up experiments to elucidate the carbon nanotube (CNT) fracture induced by laser irradiation, which included Transmission Electron Microscopy (TEM) observation, pulse width dependence (ranging from 200 fs to 2 ns), and enhancement of the G/D ratio through circularly polarised laser irradiation.
2. Further investigated the effect of surrounding thermal properties on processing quality, evaluating the influence of various processing atmospheres (air, argon, and vacuum) and substrate thermal conductivities.
3. Investigated the physical properties of laser-aligned CNTs, calculated nematic order parameter *S*, diversified evaluation methods (image analysis and visible polarisation spectroscopy), and conducted AFM observations.
4. Assessed the generalisation and practicality of laser alignment technology through a comparative analysis of CNTs synthesised by different methods using various dispersants.
5. Examined the degradation of electrical and optical anisotropy resulting from substrate carbonization.
6. Enhanced the utility of laser alignment technology by benchmarking it against competing technologies and demonstrating distinctive applications developed in this study (e.g., polarisation camera, radial polariser).
7. The experimental parameters were documented, and the Method section was further elaborated.
8. Removed content related to chirality-selective ablation and revised the author list.

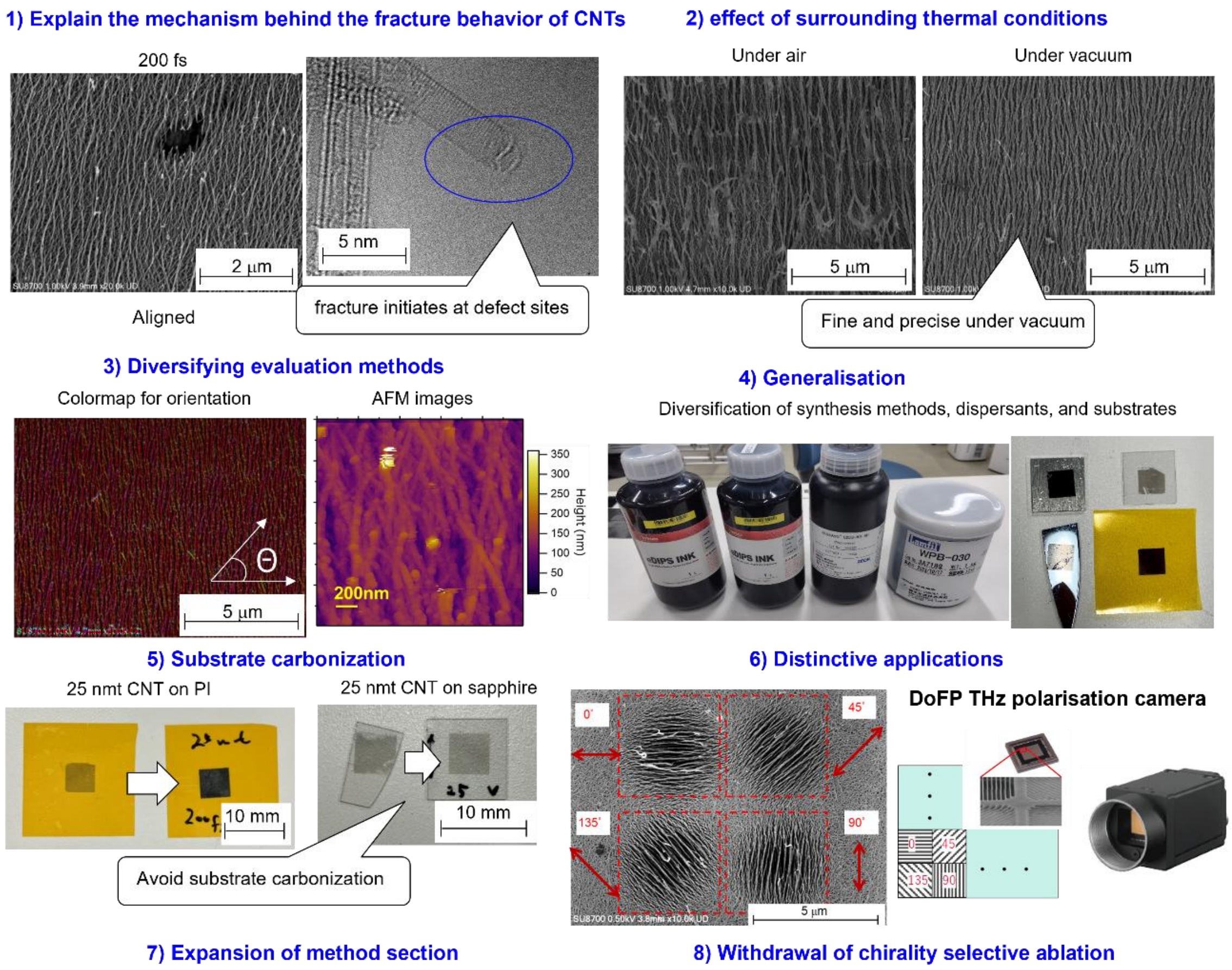

To Reviewer 1

1) The authors claimed chirality control of the aligned carbon nanotube films, however they only showed a very preliminary result of carbon nanotube film with two chiral indices. This is barely a perspective rather than practical chirality control. If chirality-specified CNTs can be prepared, why not simply prepare aligned CNTs with single chirality?

Thank you for your valuable comment. We concur that the experimental findings regarding chirality-selective ablation using ultrashort-pulses are currently preliminary. This limitation primarily arises from a mismatch between the laser wavelength and the chirality resonance peak. Fig. R1-1 displays the absorption spectra of (6,5) & (7,5) bi-chirality CNT dispersions, alongside the laser wavelength used in the experiment (1064 nm). It is apparent that the laser wavelength lies at the periphery of the (7,5) absorption spectrum, resulting in a relatively weak absorption. Consequently, our processing has only achieved a modest chirality selectivity ratio thus far. We anticipate that this issue could be addressed by precisely aligning the laser wavelength with the chirality resonance peak. However, tuning the wavelength of the ultrashort-pulsed laser requires costly equipment, such as an optical parametric amplifier. In addition, current limitations related to research funding and experimental time preclude the immediate implementation of practical chirality-selective ablation. Therefore, we have withdrawn our results and inferences regarding chirality control from the manuscript. However, we consider "chirality-selective ablation using ultrashort-pulses" to be a highly promising area, and the findings described herein may provide a valuable basis for future progress. We intend to make further practical advances in chirality-selective ablation over the next few years. We also plan to submit a subsequent manuscript to the following journal when warranted.

Based on this comment, the manuscript has been amended as follows:
i) The section addressing chirality-selective ablation has been removed.

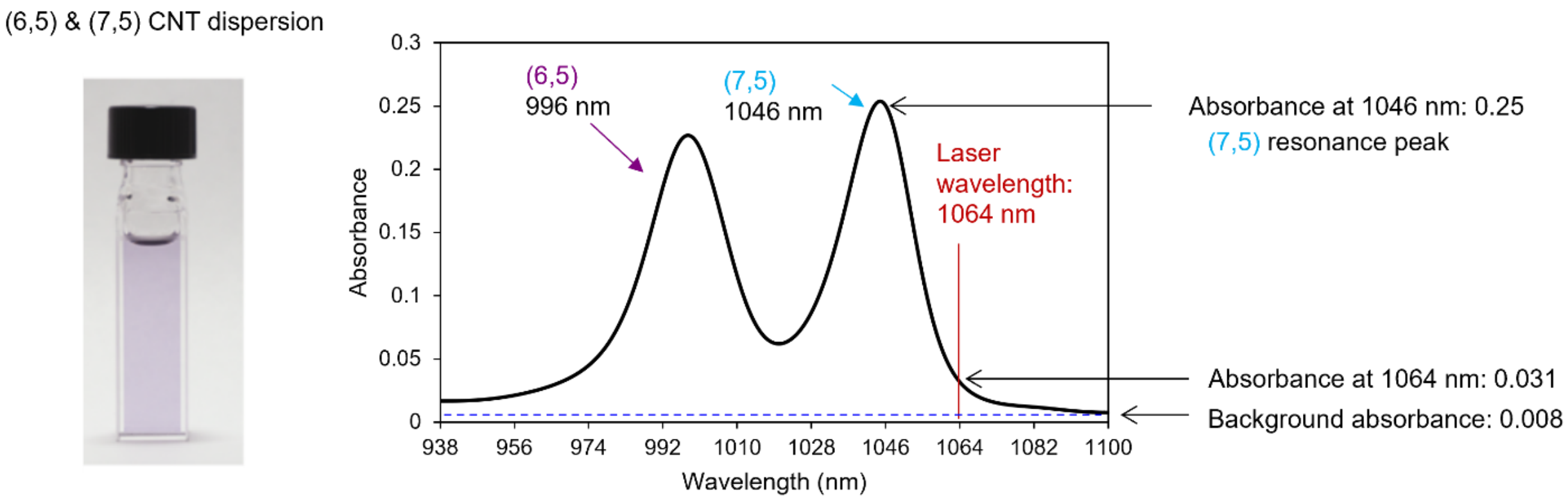

Fig. R1-1| Absorbance spectra of the bi-chirality CNT dispersions. The ultrashort pulsed laser used in the experiment operates at 1064 nm wavelength, deviating considerably from the absorption peak of (7,6) CNTs at 1046 nm.

2) The authors proposed non-thermal photon-induced destruction of carbon nanotube film for local alignment for ps laser irradiation, and photon induced thermal destruction for nanosecond and cw lasers. The key issue for successful alignment would be the thermal diffusion after laser absorption. For ps- or fs-pulsed laser irradiation, the absorbed photons would excite electrons and the excited electrons would still relax and generate heat via non-irradiative pathways. If it is non-thermal process, what would be the driving force to remove carbon nanotubes?

Thank you for your comments and question. The driving force to remove CNTs via "non-thermal" process is the vaporisation and plasma generation at the electron temperature. This is in contrast to "thermal processes", where not only electrons but also phonons reach high temperatures, leading to the destruction of CNTs. A critical parameter that governs non-thermal or thermal process is the relationship between laser pulse duration (pulse width) and the electron-phonon relaxation time. Suppose that the pulse width is shorter than the relaxation time (e.g., ranging from femtoseconds to a few picoseconds); in that case, during the pulse irradiation, the electron temperature rises, while the phonon temperature remains low due to the absence of electron-phonon collision. Once the electron temperature reaches a critical threshold, it triggers material vaporisation, plasma generation, and subsequent removal at the electron temperature.

Within the alignment processing methodology developed herein, it is essential to selectively eliminate only the laser-absorbing CNTs prior to initiating thermal diffusion into the surrounding CNTs. Figure R1-2 presents supporting experimentation, illustrating the alignment behaviour with pulse widths varying from 200 fs to 2 ns. With 200-fs and 2-ps pulses, alignment is achieved through non-thermal ablation at the electron temperature. Conversely, with a 2-ns pulse, all CNTs are removed irrespective of laser polarisation due to thermal processing at the lattice temperature. When a 20-ps pulse is used, alignment occurs; however, carbonized debris from thermal melting appears at high magnification. These observations indicate that 1) the time for thermal diffusion from a laser-absorbed CNTs into surroundings is ~10 ps, and 2) when the pulse width is below this threshold (≤10 ps), non-thermal processes enable effective alignment treatment.

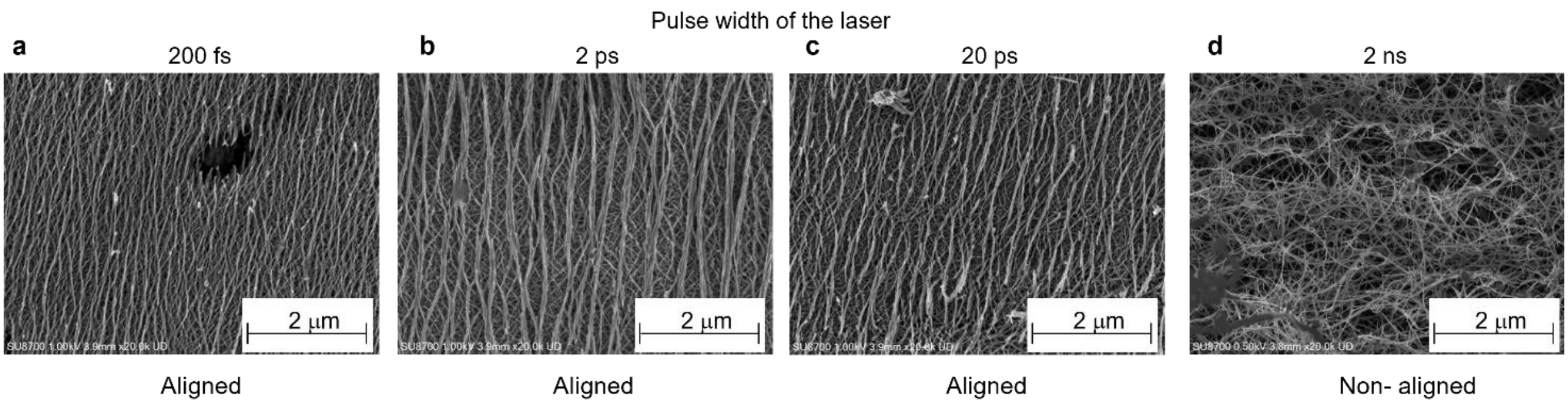

Fig. R1-2| Pulse width dependence of alignment processing. a–d, SEM images of the laser-irradiated CNTs with the laser pulse width of (a) 200 fs, (b) 2 ps, (c) 20 ps, and (d) 2 ns.

In response to this comment, the manuscript has been amended to include:
i) A comprehensive description of the laser ablation mechanism and relevant review papers.
ii) An analysis regarding the influence of pulse width on CNT alignment processing in Figure 1(f–i).

3) If thermal-related effect is the key issue, any local environment with different thermal conductivity, including the substrate and inclusion of BNNT, would affect the results. Have the authors considered and tested such effects?

We appreciate your valuable feedback. We systematically varied the substrate, attached substances, and processing atmosphere to assess the impact of the local environment with different thermal conductivity surrounding CNTs on alignment quality. All the experiments were performed under consistent laser irradiation parameters (pulse width: 200 fs; wavelength: 1030 nm; fluence: 125 mJ/cm²). Figure R1-3 presents the impact of substrate material. CNTs were transferred to four substrates with varying thermal conductivity (aluminum, silicon, sapphire, and polyimide), and laser alignment process was performed. As shown in Figure R1-3, alignment quality and threshold were

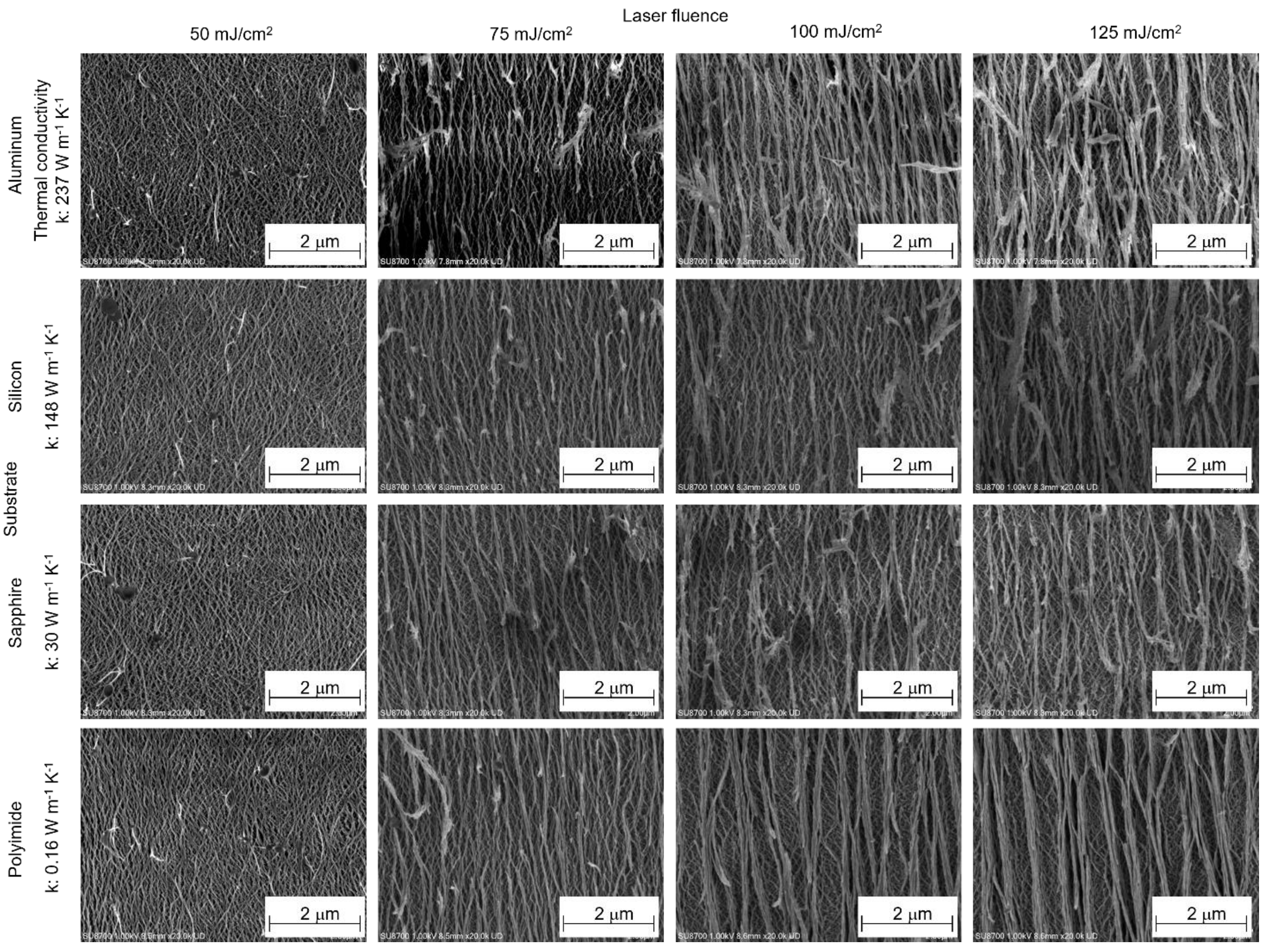

Fig. R1-3| Comparative results under various substrates.

uniform across all substrates. This suggests that the underlying mechanism of CNT alignment is non-thermal, with no heat conduction from the CNTs to the substrate (i.e., thermal conductivity of the substrate does not influence the process). This outcome was consistent even as CNT thickness decreased.

Subsequent experiments were conducted to assess the influence of attached substances with different thermal conductivity. We prepared three kinds of CNT films depositing different dispersant: carboxymethyl cellulose (thermal conductivity *k* of 0.05 W/mK), poly(acrylic acid) (*k* of 0.19 W/mK), and a surfactant (*k* of 0.25 W/mK). Figure R1-4 presents the corresponding results. The data demonstrate that alignment quality and threshold remained consistent across all samples. These observations indicate that the thermal conductivity of the attached substances does not affect the alignment processing quality, confirming the compatibility of the developed alignment technology with diverse CNT types.

Additionally, we tested the effect of different thermal diffusivity of gasses, which is the closest substance to CNTs. Figure R1-5 presents the comparative results under air, argon, and vacuum. Figures R1-5(a–c) are magnified images of laser-aligned CNTs, while Figures R1-5(d–f) depict the interface within the alignment region. The processing quality was comparable between air and argon; however, samples processed under vacuum exhibited exceptionally clean and refined alignment structures. Notably, only CNTs aligned under vacuum demonstrated seamless interfaces devoid of burrs (Figures R1-5(d–f)). These findings indicate that (i) because it is a non-thermal process, the thermal diffusivity of gas species does not affect the alignment quality, and (ii) the presence of gas molecules tends to compromise quality.

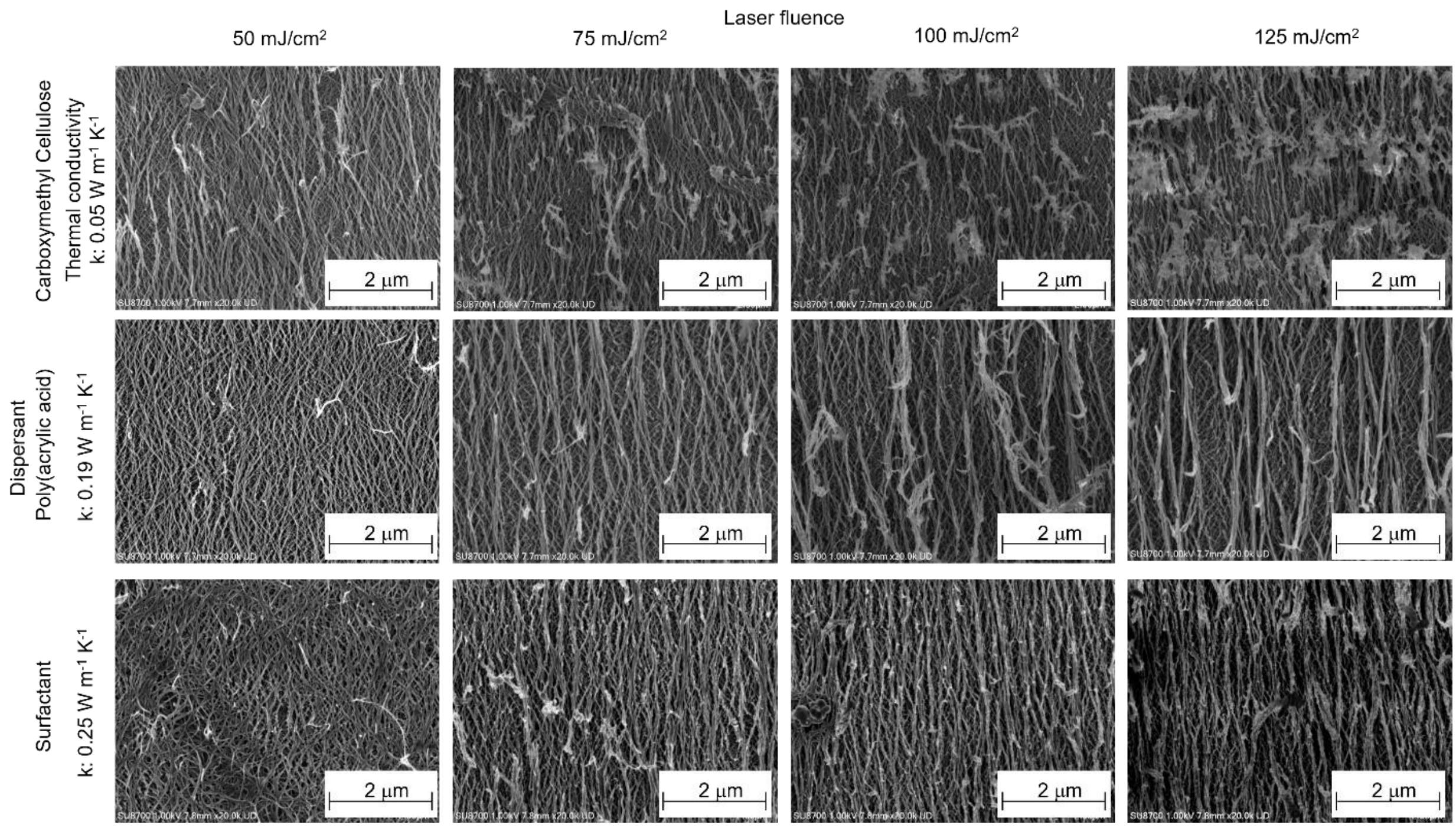

Fig. R1-4| Comparative results under various CNT dispersants.

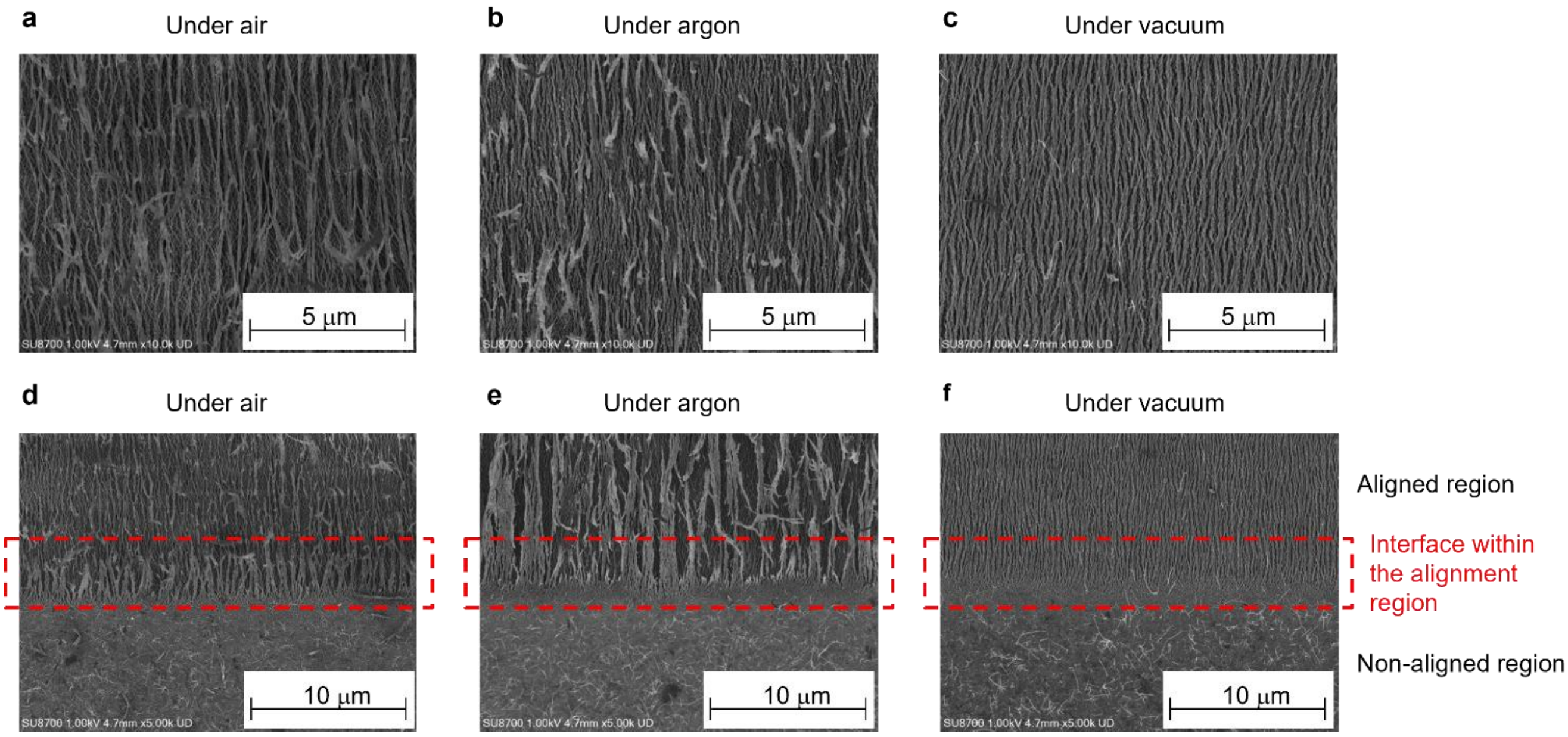

Fig. R1-5| Comparative results under various atmospheres. a–f, (a–c) Magnified images and (d–f) interface images of the laser-aligned CNTs performed under (a, d) air, (b, e) argon, and (c, f) vacuum.

Based on this comment, the manuscript has been amended to

i) Incorporate details of the influence of the processing atmosphere on sample characteristics in Figure 1(j, k) and Supplementary Figure 3.

ii) Include information regarding the impact of the dispersant on the sample data in Supplementary Figure 5.

iii) Provide insights into the effect of the substrate on the sample properties in Supplementary Figure 6.

iv) Revise the main text to include explanations for these modifications.

4) Page 4, "absorbing parallel light and transmitting light perpendicular to their length", it is "light polarisation", not "light", and "long axis" not "length".

Thank you for your suggestion. We have revised the main text accordingly.

5) Please give the information of laser wavelength, pulse-width and energy at the first place when introducing fig1 in page 4.

Thank you for pointing this out. We have included the description of the laser wavelength, pulse width, and fluence as the processing conditions for Figures 1(d, e).

6) How the spot diameter of laser spot was estimated from lens NA?

Thank you for this question. The spot diameter D was estimated using the approximate formula for the Airy disk diameter, $D = 1.22\times\lambda/\mathrm{NA}$, where λ is wavelength and NA is numerical aperture, respectively. We have added this description in the Method section.

7) Page 6, "we discuss the physical properties of laser-aligned CNT films using SEM observation and Raman spectroscopy". It is the "quality" of CNT films can be given by SEM and Raman spectroscopy.

Thank you for pointing this out. We have corrected the description "physical properties" to "quality".

8) In Fig 5, the authors showed BNNT was used, and they discussed "to improve compatibility with the CNT solutions" in the Method section. More detailed discussion should be given with appropriate references for why BNNT was used, and would BNNT affect the laser induced destruction or not.

Thank you for your valuable comment. The impact of BNNT on laser processing has yet to be comprehensively studied; therefore, it is not appropriate to present findings related to BNNT at this stage. The section addressing chirality-selective ablation has been omitted from the main manuscript. Consequently, all experimental data involving BNNT have also been excluded from the text.

To Reviewer 2

1) WHY DO THEY SAY NON-THERMAL? "ultrashort-pulsed lasers (via non-thermal processing)"For ultrashort pulses such as picosecond or femtosecond pulses, the pulse width is shorter than the intraband thermalization time of carriers (a few picoseconds)34,35; therefore, processing occurs before significant thermal diffusion, resulting in a non-thermal photon-induced destruction phenomenon." 100 ps is pretty fast must faster than the intraband relaxation time.

Thank you for your valuable comments. We express our sincere apologies for any confusion arising from the previously insufficient explanation of the principle behind laser ablation. We have incorporated a comprehensive description of the material removal mechanisms following laser irradiation to address this, referencing relevant review papers [Russo, R. E. *Appl. Phys. A* **129**, 168 (2023)].

When a laser is directed onto a CNT, it absorbs photon energy, resulting in electron excitation and a localised elevation in electron temperature (associated with the thermal kinetic energy of electrons). Subsequently, energy transfer occurs through electron-phonon collisions, raising the lattice temperature (thermal kinetic energy of phonons). The interval where this energy exchange transpires is the electron-phonon relaxation time, typically 1–100 ps for most materials [Elsayed-Ali, H.-E. et al. *Phys. Rev. Lett.* **58**, 1212 (1987); Sun, C.-K. et al. *Phys. Rev. B* **50**, 15337 (1994)]. Once the electron or lattice temperature exceeds the material's melting point, evaporation ensues, followed by plasma formation and material removal—a process that is central to laser ablation. A critical parameter here is the relationship between laser pulse duration (pulse width) and the electron-phonon relaxation time. Suppose that the pulse width is shorter than the relaxation time (e.g., ranging from femtoseconds to a few picoseconds); in that case, during the pulse irradiation, the electron temperature rises, while the phonon temperature remains low due to the absence of electron-phonon collision. Once the electron temperature reaches a critical threshold, it triggers material vaporisation, plasma generation, and subsequent removal at the electron temperature. In this scenario, phonon-mediated heat conduction is absent; thus, it is generally classified as "non-thermal processing." Conversely, under longer pulse durations—such as nanosecond pulses or continuous waves—the destructive phenomenon follows the electron-phonon collision, and material removal occurs at the lattice temperature via thermal evaporation, encompassing conventional conductive heat transfer ("thermal processing").

Within the alignment processing methodology developed herein, it is essential to selectively eliminate only the laser-absorbing CNTs prior to initiating thermal diffusion into the surrounding CNTs. Figure R2-1 presents supporting experimentation, illustrating the alignment behaviour with pulse widths varying from 200 fs to 2 ns. With 200-fs and 2-ps pulses, alignment is achieved through non-thermal ablation at the electron temperature. Conversely, with a 2-ns pulse, all CNTs are removed irrespective of laser polarisation due to thermal processing at the lattice temperature. When a 20-ps

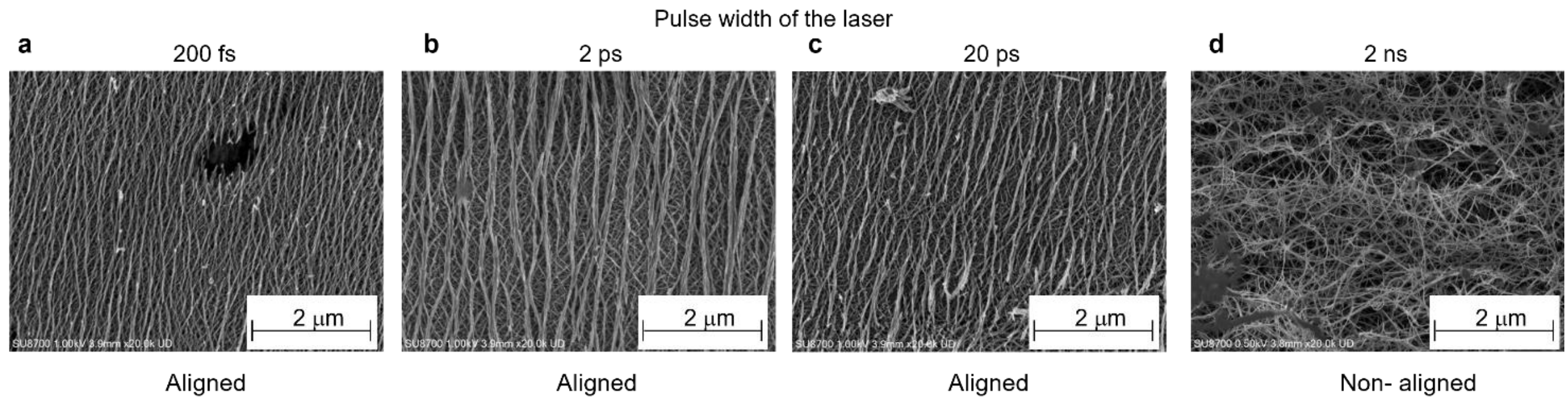

Fig. R2-1| Pulse width dependence of alignment processing. a–d, SEM images of the laser-irradiated CNTs with the laser pulse width of (a) 200 fs, (b) 2 ps, (c) 20 ps, and (d) 2 ns.

pulse is used, alignment occurs; however, carbonized debris from thermal melting appears at high magnification. These observations indicate that 1) the time for thermal diffusion from a laser-absorbed CNTs into surroundings is ~10 ps, and 2) when the pulse width is below this threshold (≤10 ps), non-thermal processes enable effective alignment treatment.

Based on this comment, the manuscript has been amended to:

i) Comprehensively describe the laser ablation mechanism and relevant review papers.

ii) Include an analysis regarding the influence of pulse width on CNT alignment processing in Figure 1(f–i).

2) They mention ablation. How do they know?

Thank you for this question. Direct observation of the destruction phenomenon of CNTs at the femtosecond scale has not yet been achieved. Nonetheless, analysis of experimental results indicates that laser ablation serves as the primary mechanism for CNT removal. As outlined in Comment 1, the nature of laser ablation-induced destruction is generally influenced by the laser pulse width. A shorter pulse width enables refined processing without significant heat diffusion to adjacent areas, whereas a longer pulse width induces thermal effects during processing. Figure R2-1 illustrates that, within our CNT alignment technology, short pulse widths selectively remove only those CNTs absorbing the laser, while longer pulse widths result in the removal of both targeted and surrounding CNTs. This dependence on pulse width aligns with the characteristics of laser ablation, supporting our conclusion that laser ablation is the driving force behind CNT removal.

3) The "wavelength-selective, chirality-dependent removal in a bichiral CNT film" is not very good. Barely any selectivity.

Thank you for pointing this out. I concur that the experimental findings regarding chirality-selective ablation using ultrashort-pulses are currently preliminary. This limitation primarily arises from a mismatch between the laser wavelength and the chirality resonance peak. Figure R2-2 displays the absorption spectra of (6,5) & (7,5) bi-chirality CNT dispersions, alongside the laser wavelength used in the experiment (1064 nm). The laser wavelength lies at the periphery of the (7,5) absorption spectrum, resulting in a relatively weak absorption. Consequently, our processing has only achieved a modest chirality selectivity ratio thus far. We anticipate that this issue could be addressed by precisely aligning the laser wavelength with the chirality resonance peak. However, tuning the wavelength of the ultrashort-pulsed laser requires costly equipment, such as an optical parametric amplifier. In addition, current limitations related to research funding and experimental time preclude the immediate implementation of practical chirality-selective ablation. Therefore, we have withdrawn our results and inferences regarding chirality control from the manuscript. However, we consider "chirality-selective ablation using ultrashort-pulses" to be a highly promising area, and the findings described herein may provide a valuable basis for future progress. We intend to make further practical advances in chirality-selective ablation over the next few years. We also plan to submit a subsequent manuscript to the following journal when warranted.

Based on this comment, the section addressing chirality-selective ablation has been removed.

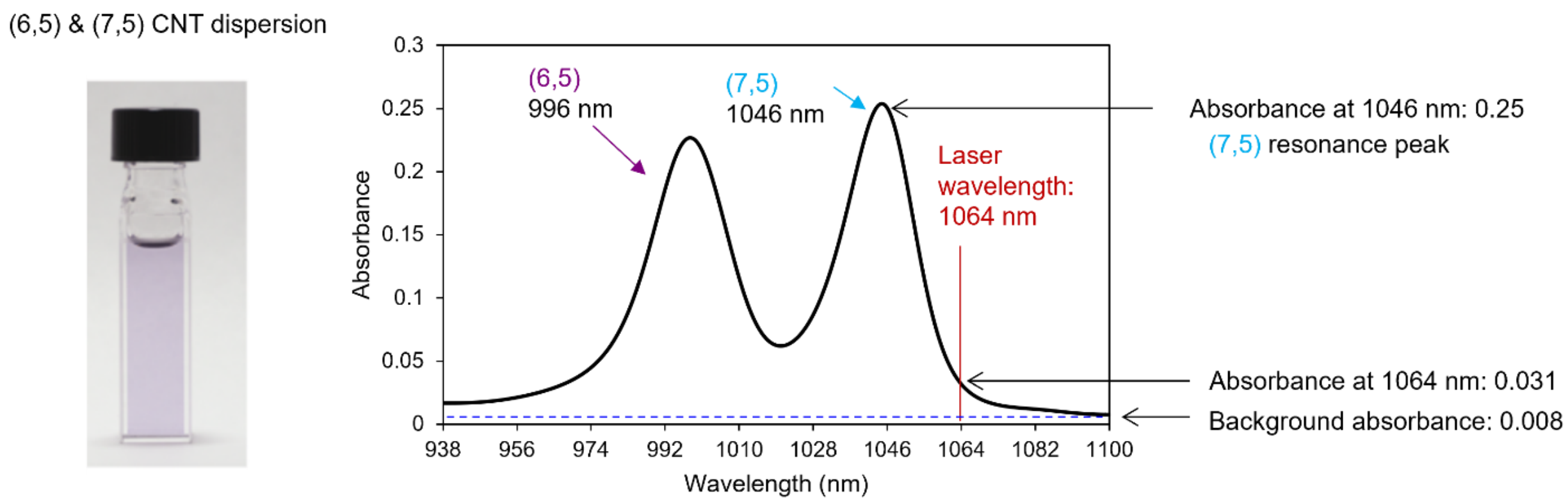

Fig. R2-2| Absorbance spectra of the bi-chirality CNT dispersions. The ultrashort pulsed laser used in the experiment operates at 1064 nm wavelength, deviating considerably from the absorption peak of (7,6) CNTs at 1046 nm.

4) What are the diameter, length, surfactant or polymer wrapper of the CNTs used in Fig. 1-4. What are their absorption spectra?

Regarding the laser alignment experiment, a metallic–semiconducting mixed carbon nanotube dispersion (eDIPS EC-2.0P) was obtained from Meijo Nano Carbon Co., Ltd. The CNTs featured diameters of 2–3 nm and lengths extending several micrometres. The dispersant was an aqueous surfactant. The corresponding absorption spectra are presented in Figure R2-3. We have added this description to the Method section.

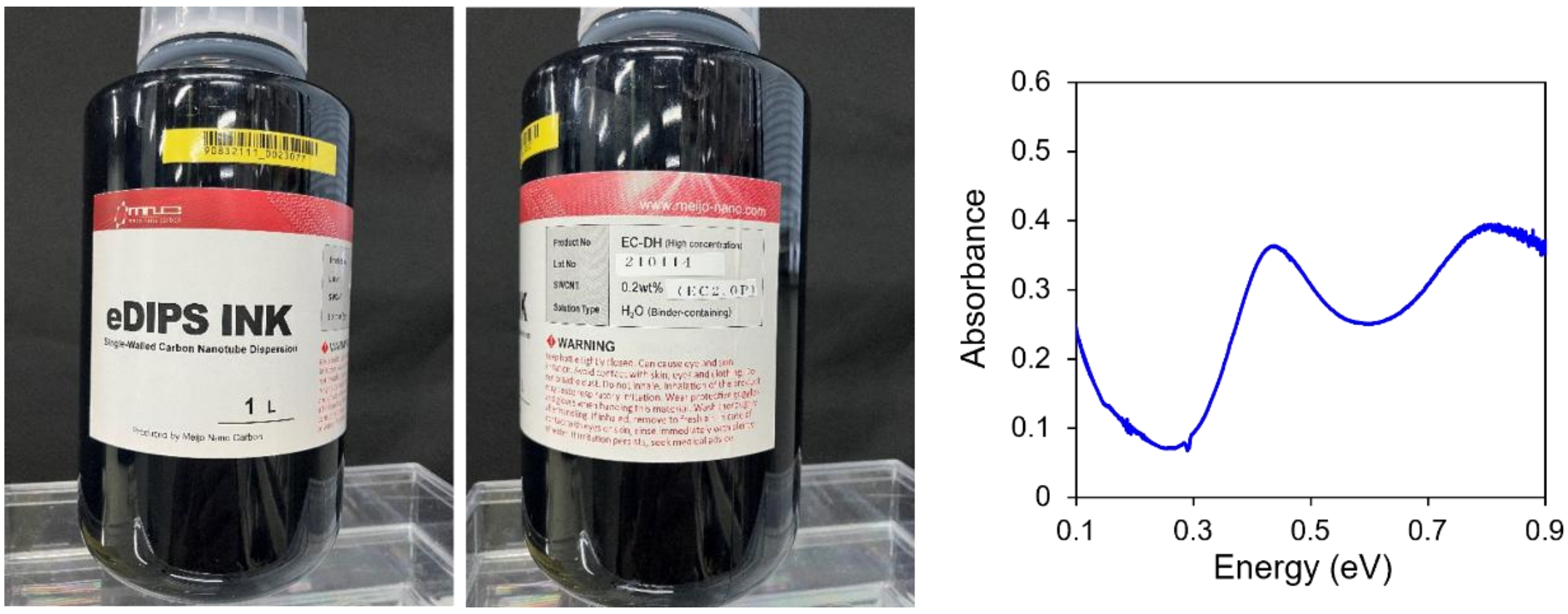

Fig. R2-3| Photographic images and absorption spectra of eDIPS EC-2.0P.

5) The mechanism is attributed to be "ablation" and "non-thermal" at the same time. Isn't ablation due to thermal heating of the carbon from the laser? Why is this called non-thermal?

Thank you for this question. Laser ablation refers to material removal via laser irradiation. Generally, laser ablation mechanisms are categorized into two types: thermal and non-thermal processes. Which process becomes dominant is determined by the interplay between the laser pulse width and the electron-phonon relaxation time of the target material. The underlying mechanism remains consistent with that described in response to comment 1. The principle of laser ablation has been explained in the main text to ensure clarity.

6) What is the removal mechanism? Heat induced oxidation? Amorphitization/vaporization? These should be differentiated by repeating experiments in vacuum or an inert environment.

We appreciate your valuable feedback and constructive comments. The removal of CNTs with ultrashort-pulses is primarily based on the mechanisms of evaporation and plasma generation induced by elevated electron temperatures. Additional experiments were performed under varying processing atmospheres—air, argon, and vacuum—to evaluate the influence of oxygen through thermal oxidation (Figure R2-4). All the experiments were performed under consistent laser irradiation parameters (pulse width: 200 fs; wavelength: 1030 nm; fluence: 125 mJ/cm^2). Figures R2-4(a–c) display magnified images of laser-aligned CNTs, while Figures R2-4(d–f) depict the interface within the alignment region. The processing quality was comparable between air and argon; however, samples processed under vacuum exhibited exceptionally clean and refined alignment structures. Notably, only CNTs aligned under vacuum demonstrated seamless interfaces devoid of burrs. These findings indicate that (i) oxygen (thermal oxidation) does not influence alignment quality because the process is non-thermal, and (ii) the presence of gas molecules tends to compromise quality.

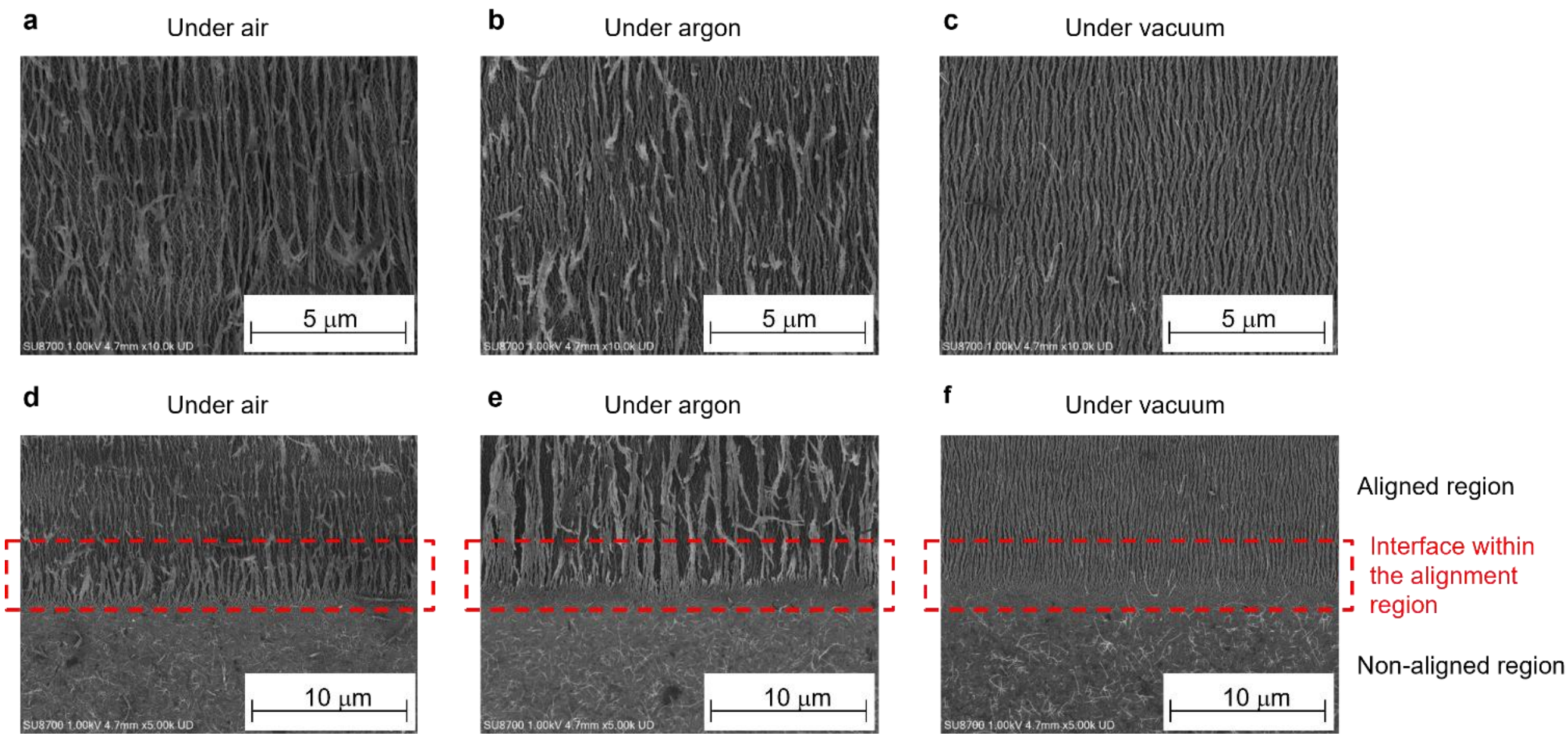

Fig. R2-4| Comparative results under different atmospheres. a–f, (a–c) Magnified images and (d–f) interface images of the laser-aligned CNTs performed under (a, d) air, (b, e) argon, and (c, f) vacuum.

In response to this comment, the manuscript has been amended to:

i) Incorporate details of the influence of the processing atmosphere on sample characteristics in Figure 1(j, k) and Supplementary Figure 3.

ii) Revise the main text to include explanations for these modifications.

7) The ability to selectively heat up one chirality of CNT or one orientation of CNT and thereby remove it, hinges on poor thermal conductance of CNT-CNT and CNT-substrate interfaces. The selectivity seems to be much better in Figs. 1-4 than Fig. 5. Why? What is the nature of CNT-CNT interfaces of the films in Figs. 1-4, is there surfactant or polymer in between the CNTs to help decouple CNTs oriented in one direction from CNTs oriented in the orthogonal direction?

Thank you for your valuable questions. Regarding the difference in selectivity between Figure 1-4 and Figure 5 in the initial manuscript, Figure 1-4 shows polarisation-dependent alignment processing, while Figure 5 shows wavelength-dependent chirality-selective processing. Since different selection processes are performed using different principles, it is natural that the selectivity will differ. The lower chirality selectivity is ascribed to the laser wavelength deviating from the chirality resonance peak (as mentioned in response to comment 3). Research on chirality-selective ablation is still at an early stage; therefore, this section has been removed from the manuscript.

In addition, we systematically varied the dispersant and the substrate to examine the thermal conduction effects between CNT-CNT and CNT-substrate interfaces. First, I will present the findings related to the impact of the dispersant. We prepared three kinds of CNT films depositing different dispersant: carboxymethyl cellulose (thermal conductivity *k* of 0.05 W/mK), poly(acrylic acid) (*k* of 0.19 W/mK), and a surfactant (*k* of 0.25 W/mK). Figure R2-5 presents the corresponding results. The data demonstrate that alignment quality and threshold remained consistent across all samples.

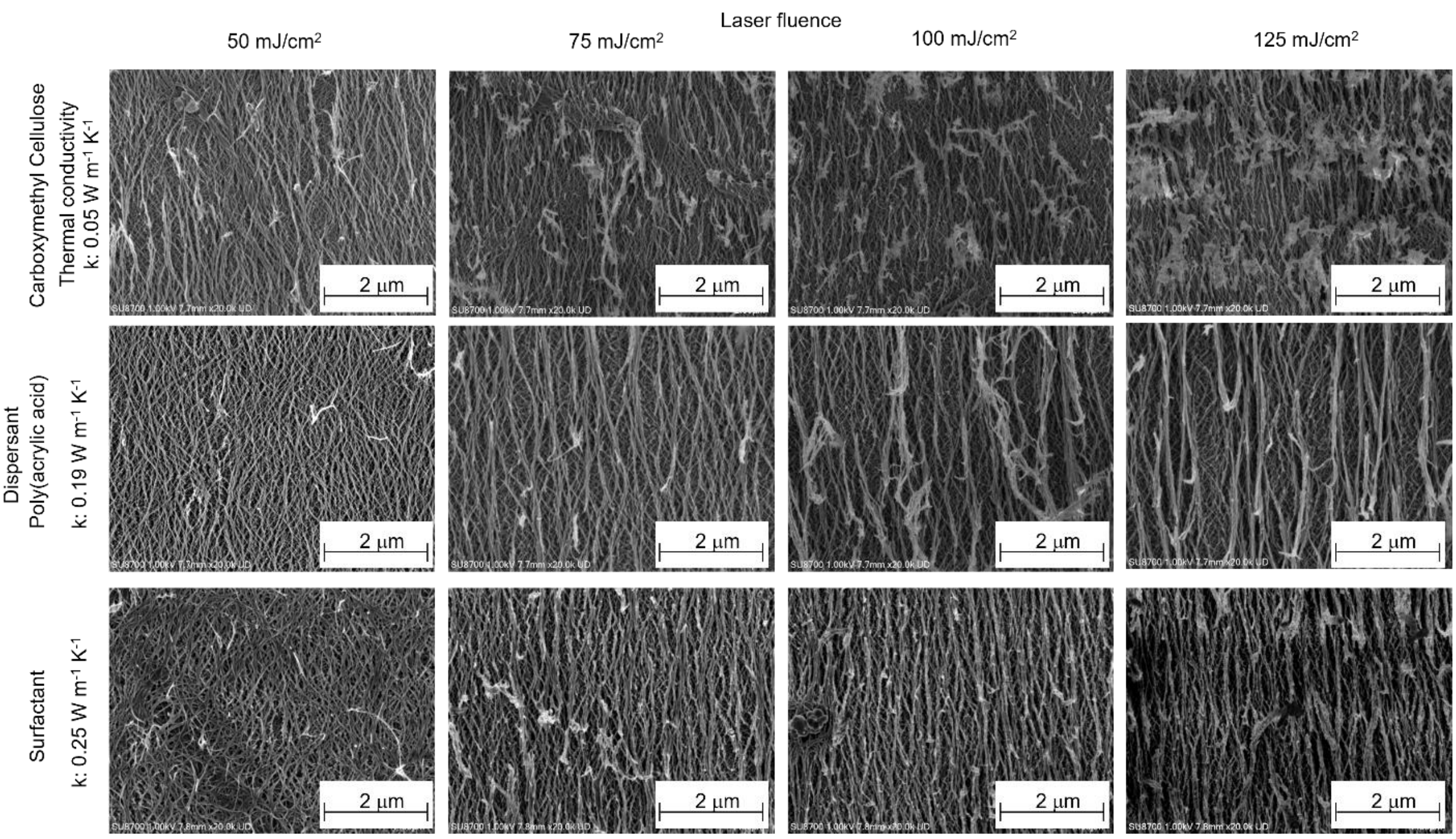

Fig. R2-5| Comparative results under different CNT dispersants.

We next investigated the impact of the substrate material. CNTs were transferred to four substrates with varying thermal conductivity (aluminium, silicon, sapphire, and polyimide), and the laser alignment process was performed (Figure R2-6). As observed with dispersant variations, alignment quality and threshold were uniform across all substrates. This suggests that the underlying mechanism of CNT alignment is non-thermal, with no heat conduction from the CNTs to the substrate (i.e., thermal conductivity of the substrate does not influence the process). This outcome was consistent even as CNT thickness decreased. However, laser-induced burning and carbonization of certain substrates were observed. Figure R2-7 summarizes the effects of substrate carbonization. Specifically, for polyimide substrates, laser transmission through the CNTs resulted in carbonization, forming laser-induced graphite (Figure R2-7(a, b)). The isotropic absorption properties of the laser-induced graphite led to a decline in the optical and electrical anisotropy of the laser-aligned CNTs (Figure R2-7(c)). Substrate carbonization was eliminated by substituting the substrate with laser-resistant sapphire—even for thin CNT films—and the optical and electrical anisotropy improved markedly, achieving 100% transmittance for perpendicularly polarised light (Figure R2-7(d–f)). In conclusion, the thermal conductivity of the substrate does not influence processing quality due to the non-thermal nature of the method; however, non-laser-resistant substrates undergo carbonization, subsequently degrading processing quality.

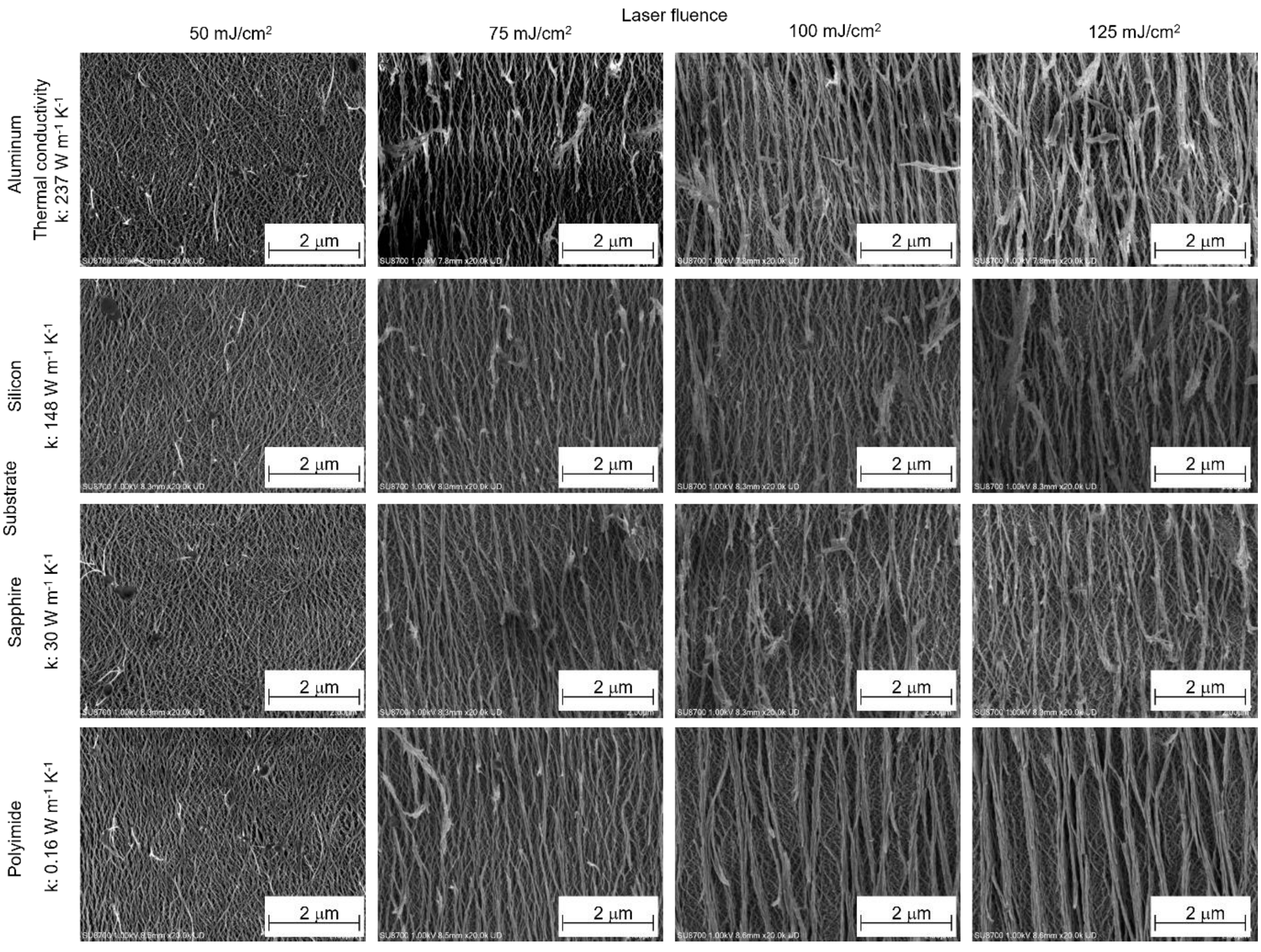

Fig. R2-6| Comparative results under various substrates.

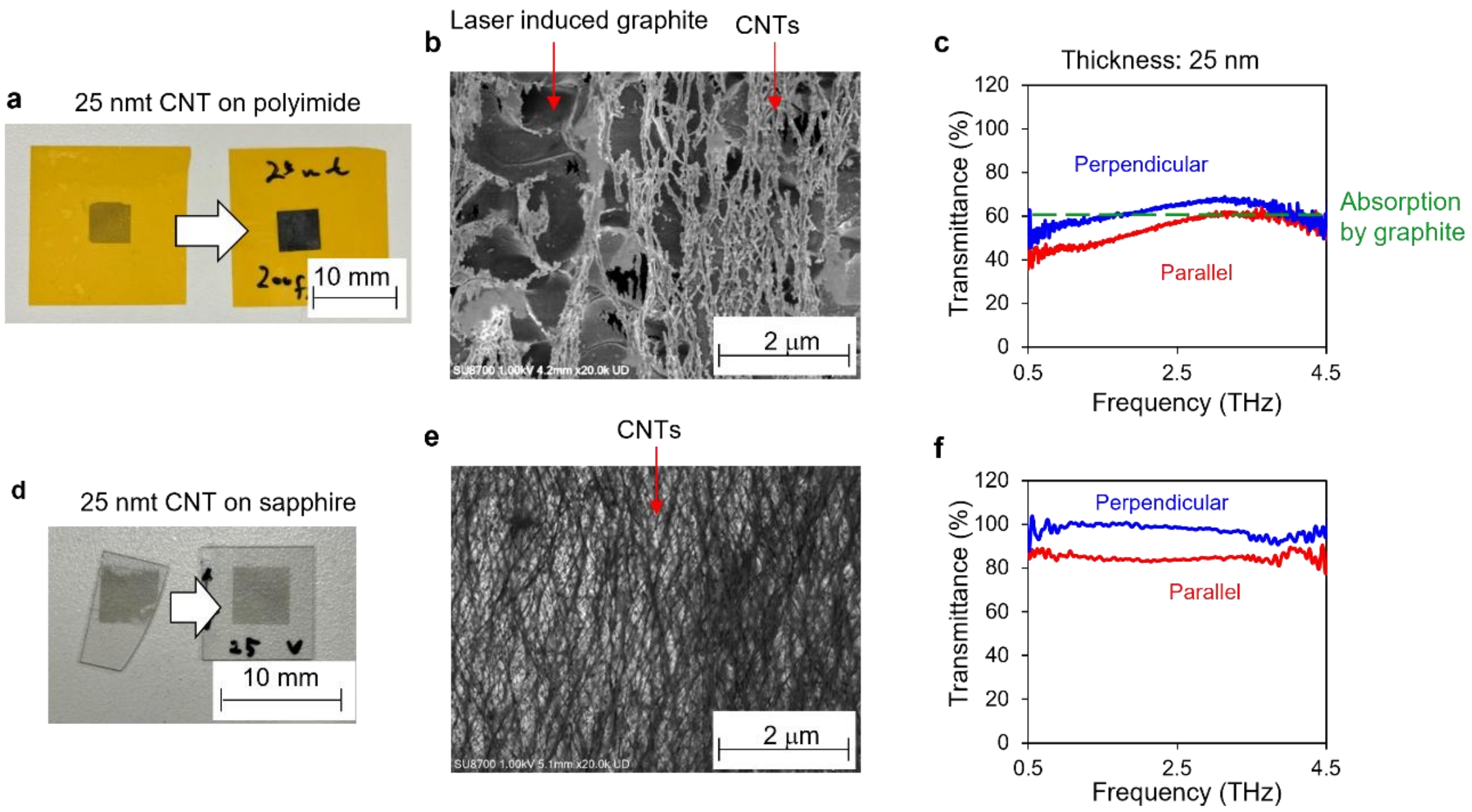

Fig. R2-7| Effects of substrate carbonization. a–c, Laser-aligned CNTs on the polyimide substrate. (a) photographic image before and after laser irradiation. (b) SEM image of the CNTs and laser-induced graphite. (c) THz transmission spectra. **d–f**, Laser-aligned CNTs on the sapphire substrate. (d) photographic image before and after laser irradiation. (e) SEM image of the CNTs. (f) THz transmission spectra.

These observations indicate that the thermal conductance of CNT-CNT and CNT-substrate interfaces does not contribute to alignment quality, confirming the compatibility of the developed alignment technology with diverse CNT types.

With respect to your inquiry regarding whether the dispersant or polymer facilitates decoupling between CNTs and thereby promotes alignment, we are unable to provide a definitive answer as the decoupling effect has not been quantitatively assessed. Most of the dispersant is removed during deposition, but some remains on the surface of these bundles. Consequently, CNT bundles with varying orientations may be in direct contact, or, as noted by the reviewer, traces of dispersant could remain between bundles. However, as illustrated in Fig. R2-5, experimental outcomes—such as threshold and alignment quality—remained consistent despite employing dispersants with differing particle sizes and thermal conductivities (0.05, 0.19, 0.25 W/mK). These findings indicate that the mechanism underlying laser ablation-based CNT alignment does not depend on the presence of the dispersant or polymer.

In response to this comment, the manuscript has been amended to:

i) Include information regarding the impact of the dispersant on the sample data (Supplementary Figure 5).

ii) Provide insights into the effect of the substrate on the sample properties (Supplementary Figure 6).

iii) Document findings related to substrate carbonization's impact on samples (Supplementary Figure 7).

iv) Revise the main text to discuss these modifications.

8) 10 ps is plenty of time for excited electron-hole pairs in CNTs to relax and give off phonons. In unsorted nanotube films, this can occur in 100's of fs, much faster than the 10 ps pulse width.

Thank you for your comment. The electron-phonon relaxation time in CNTs is influenced by several parameters, including chirality, length, bundle diameter, dispersant, and surface morphology. Previous studies have documented relaxation times spanning from a few ps to ~10 ps [Dyatlova, O. A. et al., Nano Lett. **12**, 2249 (2012); Köhler, C. *et al*., J. Phys.: Condens. Matter **25**, 105301 (2013)]. Additionally, our subsequent experiments, which varied pulse widths between 200 fs and 2 ns (Figure R2-1), revealed that optimal alignment processing of CNTs occurred at a pulse width of 2 ps, whereas pulse widths of 20 ps led to carbonization due to thermal melting. The precise determination of relaxation times necessitates ultrafast techniques such as pump-probe spectroscopy; nonetheless, our results indicate that the threshold for effective alignment processing is approximately 10 ps.

9) What the authors term "alignment degree" is more often called the "nematic order parameter" or just "order parameter"?

Thank you for your question. We apologize for any inconvenience caused by our writing style. We have calculated the nematic order parameter S and corrected the reference formatting.

10) Why are the BNNTs used in the 6,5/7,5 films?

Thank you for your question. The impact of BNNT on laser processing has yet to be comprehensively studied; therefore, it is not appropriate to present findings related to BNNT at this stage. The section addressing chirality-selective ablation has been omitted from the main manuscript. Consequently, all experimental data involving BNNT have also been excluded from the text.

To Reviewer 3

1) I do not agree with the statement: "The spatial controllability of conventional control techniques is limited to the millimetre scale, which hampers their micrometre integration." While this may apply to certain shear-based methods (e.g., LS and LB), dielectrophoresis, and controlled vacuum filtration, it does not apply to techniques such as CVD alignment [1], lithographically defined microscopic water features [2], the soft-lock method [3], or DNA nanotrenches [4]. I advise the authors to reformulate this statement while considering the relevant literature. I also recommend citing recent reviews that summarize state-of-the-art alignment techniques:

Thank you for your constructive feedback. We have referenced the suggested paper and revised the description of the research background accordingly.

2) I find the title somewhat confusing. The term chirality control may be interpreted as transformation of one chirality into another, whereas the work presented here corresponds more to selective enrichment or isolation. I advise a slight modification of the wording to avoid confusion with true chirality engineering, as discussed in this review:

Thank you for this comment. You have correctly pointed out that our methodology targets the removal of specific chiralities from a mixture containing multiple chiralities; therefore, it is more appropriate to refer to this process as "purification" or "separation." Furthermore, other reviewer pointed out that the experimental findings regarding chirality-selective ablation using ultrashort-pulses are currently preliminary. This limitation primarily arises from a mismatch between the laser wavelength and the chirality resonance peak. Fig. R3-1 displays the absorption spectra of (6,5) & (7,5) bi-chirality CNT dispersions, alongside the laser wavelength used in the experiment (1064 nm). It is apparent that the laser wavelength lies at the periphery of the (7,5) absorption spectrum, resulting in a

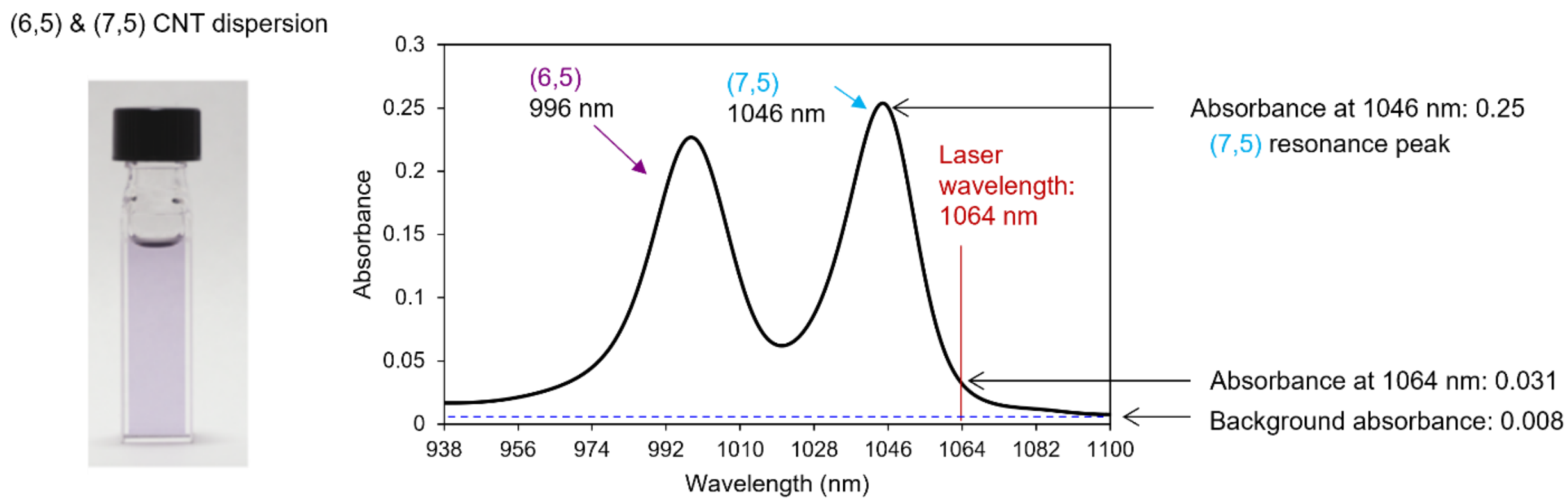

Fig. R3-1| Absorbance spectra of the bi-chirality CNT dispersions. The ultrashort pulsed laser used in the experiment operates at 1064 nm wavelength, deviating considerably from the absorption peak of (7,6) CNTs at 1046 nm.

relatively weak absorption. Consequently, our processing has only achieved a modest chirality selectivity ratio thus far. We anticipate that this issue could be addressed by precisely aligning the laser wavelength with the chirality resonance peak. However, tuning the wavelength of the ultrashort-pulsed laser requires costly equipment, such as an optical parametric amplifier. In addition, current limitations related to research funding and experimental time preclude the immediate implementation of practical chirality-selective ablation. Therefore, we have withdrawn our results and inferences regarding chirality-selective ablation from the manuscript. Correspondingly, the title has been revised to 'Ultrashort-pulse optical post-processing for local alignment control in carbon nanotube films." However, we consider "chirality-selective ablation using ultrashort-pulses" to be a highly promising area, and the findings described herein may provide a valuable basis for future progress. We intend to make further practical advances in chirality-selective ablation over the next few years. We also plan to submit a subsequent manuscript to the following journal when warranted.

3) While the method shows a clear advantage in enabling in situ local alignment, it does not demonstrate superiority over other techniques in terms of morphology uniformity or packing density. When discussing the advantages, I invite the authors to benchmark their material against the state of the art. For example, DLSA yields ~200 tubes/μm [1], and controlled vacuum filtration ~1000 tubes/μm [2]. Where does the present technique stand in terms of packing density?

Thank you for your suggestion. The laser alignment technique presented in the paper involves removing undesired CNTs from a randomly assembled matrix, resulting in morphological uniformity and packing density that are less optimal compared to existing alignment methods. However, it is important to clarify that the morphological uniformity and packing density achieved through laser alignment are contingent upon the initial CNT structure. Figure R3-2 presents the key considerations and recommendations for enhancing performance. In the experiment discussed, an aligned film was produced by eliminating unwanted CNTs from a completely un-aligned film, resulting in a packing density of approximately 30 bundles per micron (Figure R3-2(a), where each bundle comprises approximately 10 to 100 CNTs). We propose that this limitation can be addressed using pre-aligned films based on established techniques. For instance, the Arnold group in the United States has demonstrated aligned CNTs using the dipping method (Figure R3-2(b)). Applying our laser alignment technology to their pre-aligned CNTs may further enhance morphological uniformity and packing density, leveraging the principle of purification via laser irradiation. Likewise, the Kono and Yanagi group has achieved highly aligned CNTs using a suction filtration approach. We can obtain locally aligned CNTs with increased packing density by stacking their alignment films with varied orientations and implementing our laser alignment technology (Figure R3-2(c)). Thus, the packing density and uniformity achieved with our laser alignment technique are primarily governed by the material properties rather than the process itself. Integrating this technology with conventional alignment

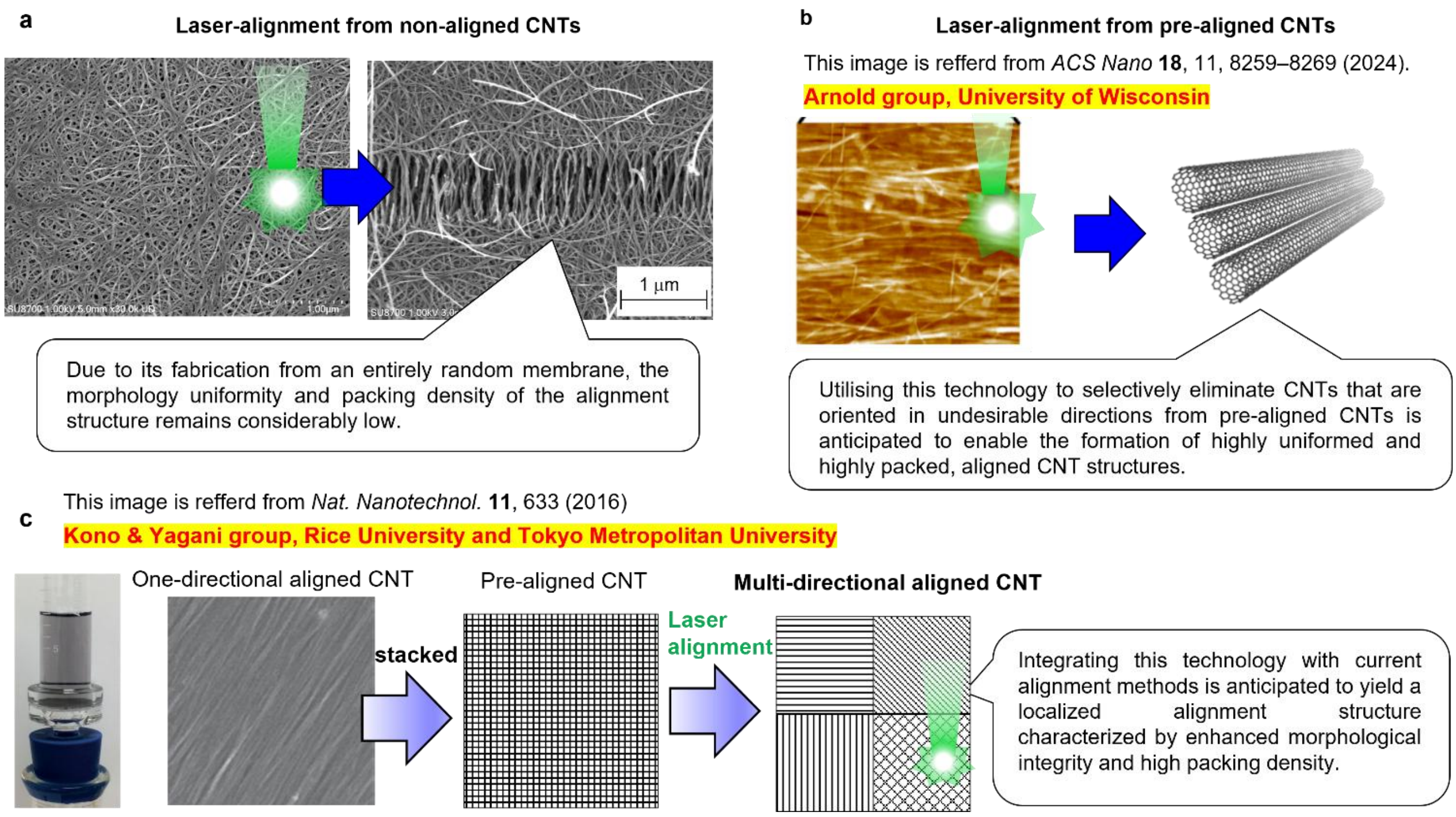

Fig. R3-2| Key considerations and future plans for enhancing performance. a, Laser-alignment from non-aligned CNTs (current stage). **b**, Concept of the laser-alignment from pre-aligned CNTs. **c**, Concept of the multi-directional aligned CNTs by integrating the laser alignment technology and conventional alignment methods.

methods can considerably improve performance, benefiting our approach and alternative technologies.

As noted by the reviewers, laser alignment technology demonstrates significant strength and uniqueness through its capability to precisely control local alignment structures. We performed additional experiments resulting in the formation of intricate structures as depicted in Figure R3-3 to further highlight this advantage. This technology enables the creation of orientations with CNTs at 0°, 45°, 90°, and 135°, as well as configurations arranged in a circular pattern. These novel formations are anticipated to serve as foundational technologies for DoFP-type THz polarisation cameras and radial polarisers. The innovative aspect of this approach is its capacity to generate complex structures unattainable by traditional alignment methods, supporting its suitability for publication in *Nature Communications*.

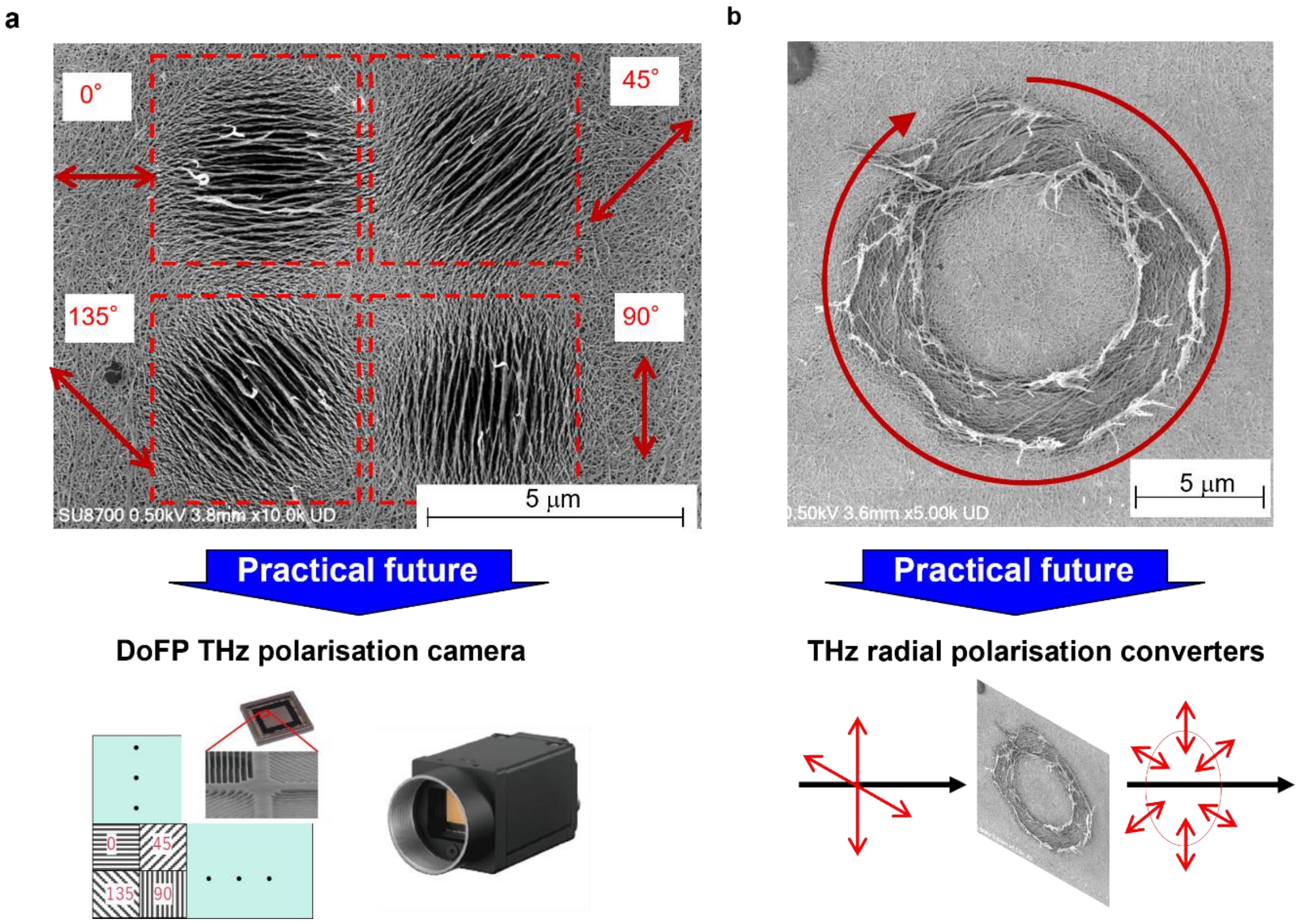

Fig. R3-3| Complex CNT alignment structure formed using laser alignment technology. **a**, Integrated CNT films with four distinct orientations (0°, 45°, 90°, and 135°). **b**, Aligned CNTs arranged in a circular pattern.

4) Meijo eDIPS CNTs have diameters of ~1.5–3 nm and cannot be resolved individually by SEM. I therefore advise the authors to describe the aligned structures as bundles or fibers rather than individual CNTs. It is also possible that multiple CNT orientations exist within an aligned bundle. One way to address this would be to isolate a single bundle and characterize it by TEM. I also recommend adding more details on the CNT material used.

Thank you for your suggestion. We used eDISP EC-2.0P (Meijo Nanocarbon), yielding an average diameter of 2–3 nm. As indicated by the reviewer, individual CNTs are not detectable via SEM; therefore, the alignment structures observed in SEM images represent CNT bundles with diameters of 20–50 nm. We have added to the main text that the alignment structures observed by SEM are not individual CNTs but rather form bundles. TEM observations were conducted on laser-aligned CNTs for further investigation (Figure R3-4). We deposited a CNT dispersion onto a TEM grid and selectively irradiated it using an ultrashort pulse laser. The results demonstrated that alignment processing is feasible even in thin films composed of several CNTs. At this stage, the bundle diameter measured approximately 20–50 nm.

TEM observation provided us with many insights into the fracture behaviour of CNTs due to laser irradiation. Our response on CNT quality is provided in comment 7.

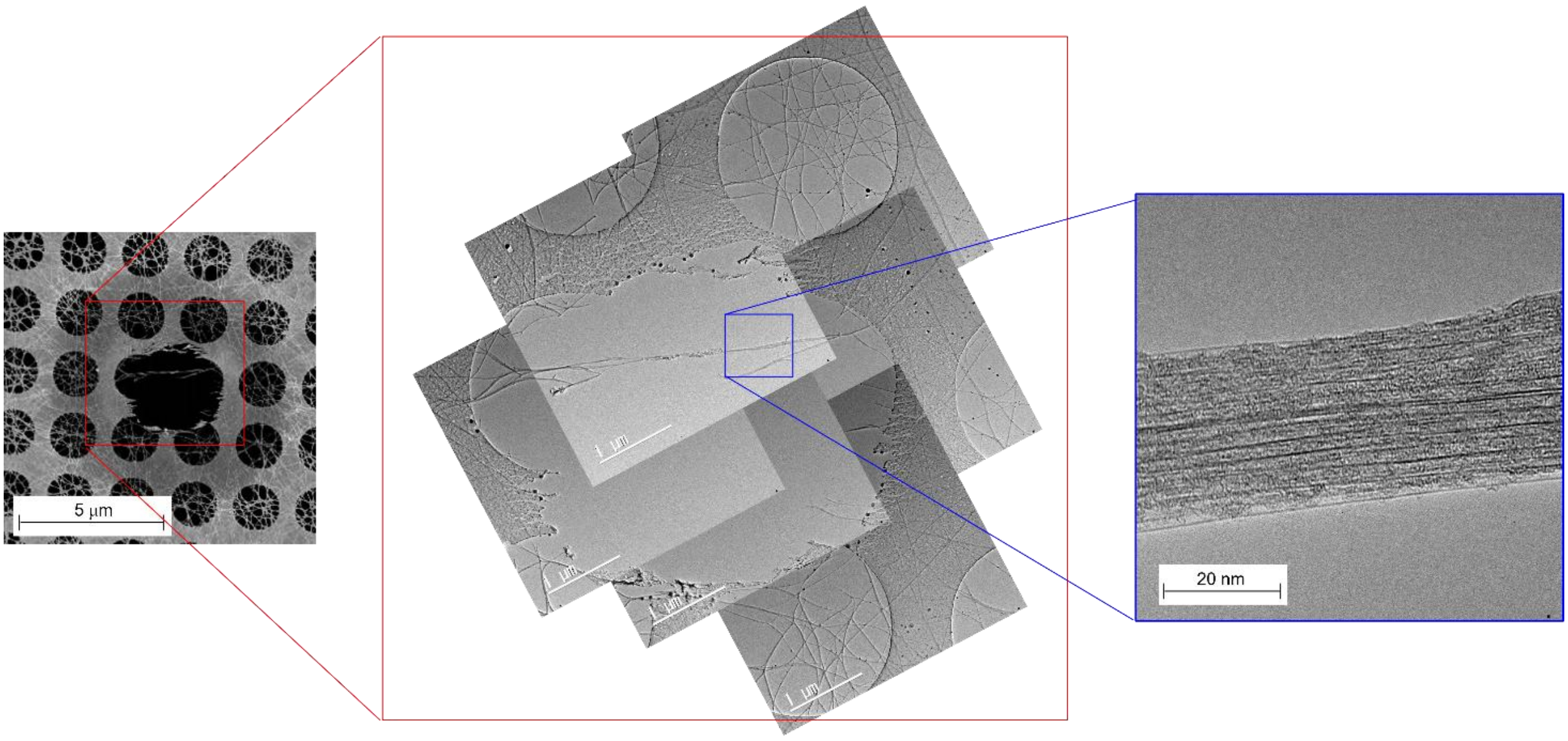

Fig. R3-4| SEM and TEM images of the laser-aligned CNTs.

The manuscript has been amended as follows:

i) We have added to the main text that the alignment structures observed by SEM are not individual CNTs but rather form bundles.

ii) We have added the TEM images of laser-aligned CNTs to Figure 4 and Supplementary Figure 2.

iii) We have added details about the CNT being used to the Method section.

5) Two techniques are used to quantify alignment. THz spectroscopy is very forgiving, as a 2 THz wavelength (~150 μm) is much larger than the CNT dimensions. Raman is highly local and probes only the top surface. I strongly advise the authors to characterize alignment using UV–Vis–NIR polarised absorption, ideally probing S11 and M11 transitions. To enable comparison with the literature, the degree of alignment should be reported as the nematic order parameter S, which is the standard metric for nematic systems.

Thank you for your insightful feedback. In accordance with your suggestions and to diversify the methods of alignment evaluation, we conducted polarised visible spectroscopy and image analysis in addition to polarised Raman and polarised THz spectroscopy. We also evaluated the nematic order parameter *S* based on the recommended paper (Nano Lett. 2008, **8**, 12, 4103–4107).

Figure R3-5 shows the polarised absorbance characteristics in visible frequency region, indicating that the laser-aligned CNTs work as a polariser in visible frequency region as same as in THz frequency region.

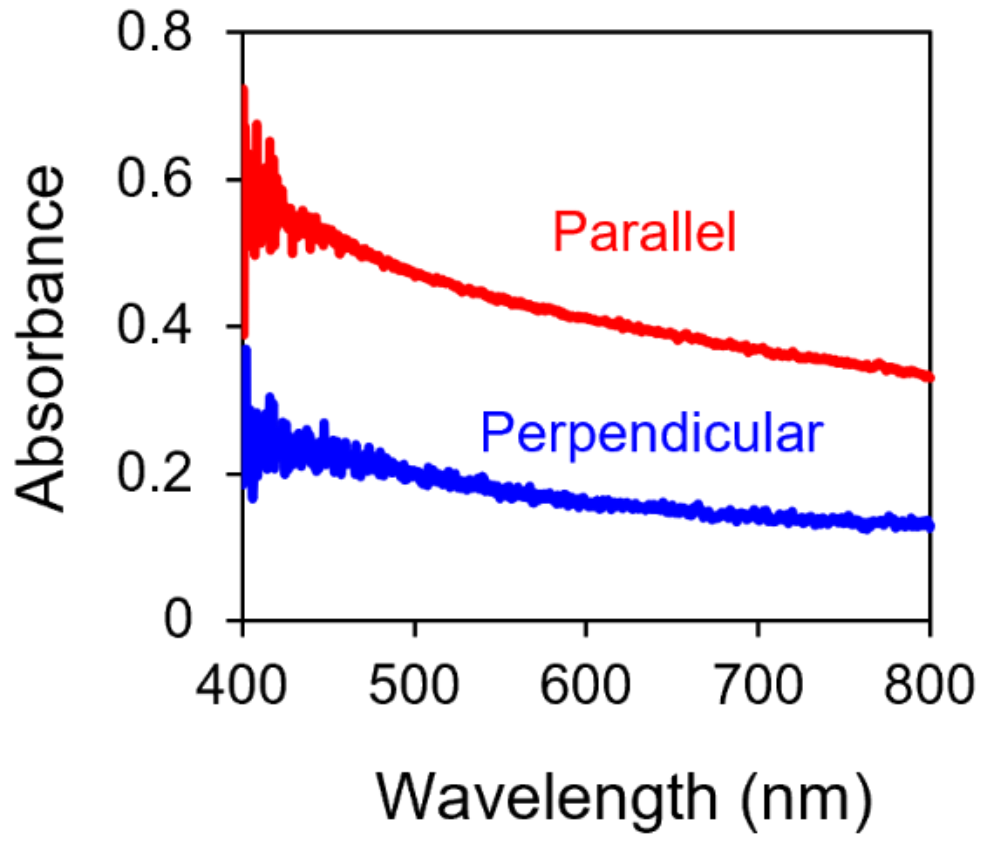

Fig. R3-5| Polarised absorbance characteristics in visible frequency region.

In addition, the results of the image analysis performed to calculate the nematic order parameter *S* are shown in Figure R3-6. Connected pixel components were extracted based on angular orientation using SEM images of laser-aligned CNTs. The alignment colormaps and distributions of the laser-aligned CNTs demonstrate a pronounced vertical alignment (Figure R3-6(b, c)). Conversely, the pristine CNTs without laser treatment exhibited disordered results (Figure R3-6(d–f)). The 2D order parameter *S* was determined using the angular data and calculated as S = 0.93 via the formula:

$$S = \langle 2\cos^2\theta - 1 \rangle. \quad \text{(R1)}$$

These results demonstrate a high level of alignment compared to existing alignment techniques as

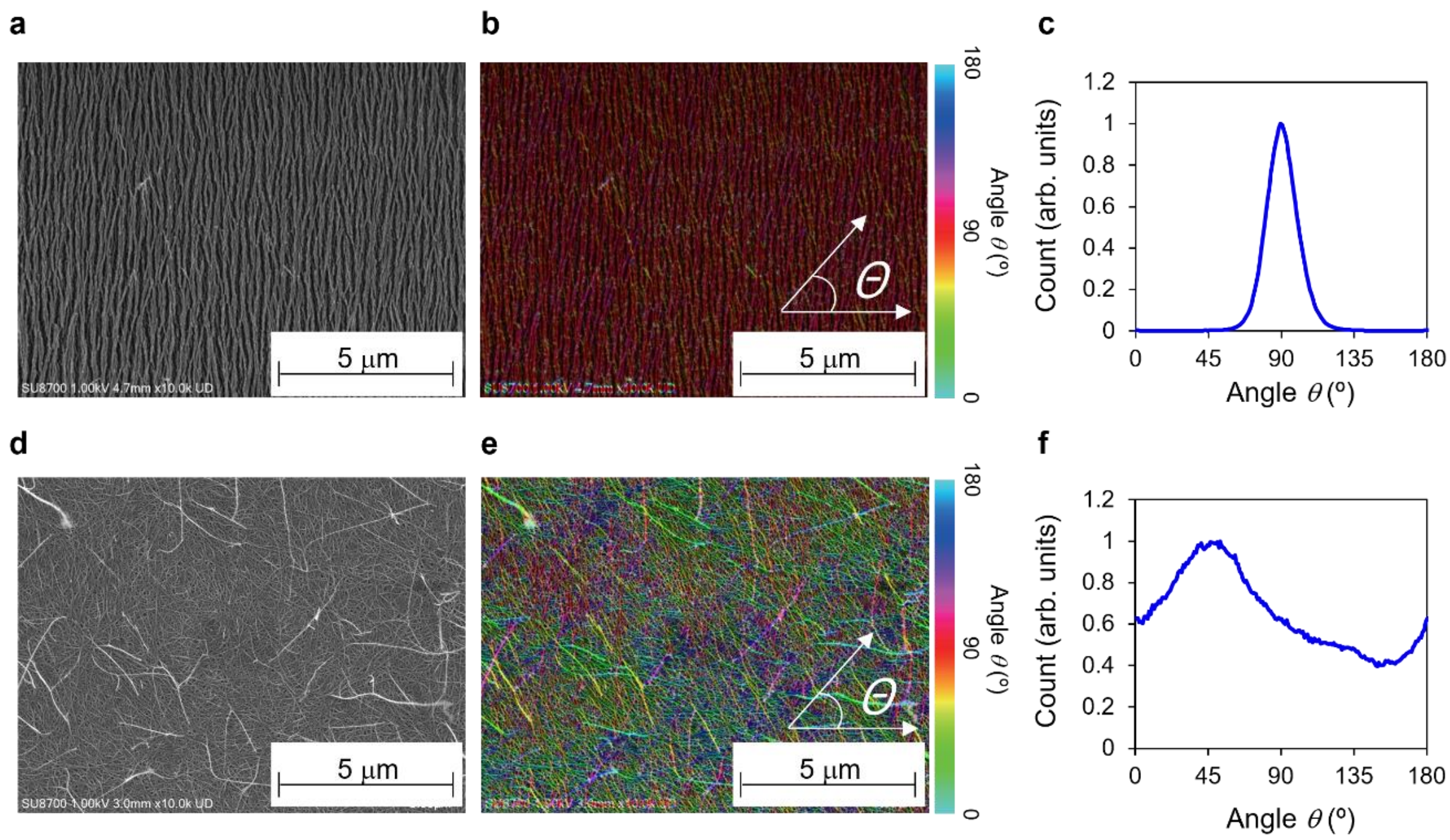

Fig. R3-6| Evaluation of alignment degree through image analysis. a–c, (a) SEM image, (b) colormap for orientation, (c) and distribution for orientation of the laser-aligned CNTs. **d–f**, (d) SEM image, (e) colormap for orientation, and (f) distribution for orientation of the pristine CNTs.

discussed in this review:

Chen, Y. et al. Controlled Assembly of Single-Walled Carbon Nanotubes into Thin Films and Their Applications. *Adv. Funct. Mater.* **35**, 2426084 (2025).

The manuscript has been amended as follows:

i) We have added polarised spectroscopy in the visible frequency region and the results of the image analysis in Figure 5.

ii) We have calculated the nematic order parameter S and described it in the main text.

6) In the Raman measurements, circularly polarised excitation is used. I advise using linearly polarised excitation and reporting I_{VV}, I_{HH} and I_{VH} along with the dichroic ratio δ, and extracting a local nematic order parameter S. The incident polarisation can be controlled by rotating the sample relative to the incident linear polarisation while probing the same spot.

Thank you for your comment. As additional experiments in the response to comment 5, we have assessed the nematic order parameter S using polarised Raman spectroscopy. Figure R3-7 presents the experimental findings. For Raman spectroscopy, both the excitation and detection light polarisations were controlled via a linear polariser, enabling measurements of peak intensities for the G band I_{VV}, I_{VH}, and I_{HH} (Figure R3-7(b)). The excitation wavelength used for Raman analysis was 532 nm. Observed peak intensities for the G band were I_{VV} = 15.4, I_{VH} = 2.3, and I_{HH} = 1.6, indicating pronounced polarisation characteristics. And dichroic ratio Δ (absorbance ratio relative to polarisation) at a 532 nm wavelength was 2.34 calculated from Figure R3-5. According to the polarised Raman intensities (I_{VV}, I_{VH}, and I_{HH}) and the dichroic ratio Δ, 2D order parameter S (Equation R1) can be calculated as follows:

$$S = \frac{\Delta I_{\mathrm{VV}} - I_{\mathrm{HH}}}{\Delta I_{\mathrm{VV}} + I_{\mathrm{HH}} + (1+\Delta) I_{\mathrm{VH}}}. \qquad \text{(R2)}$$

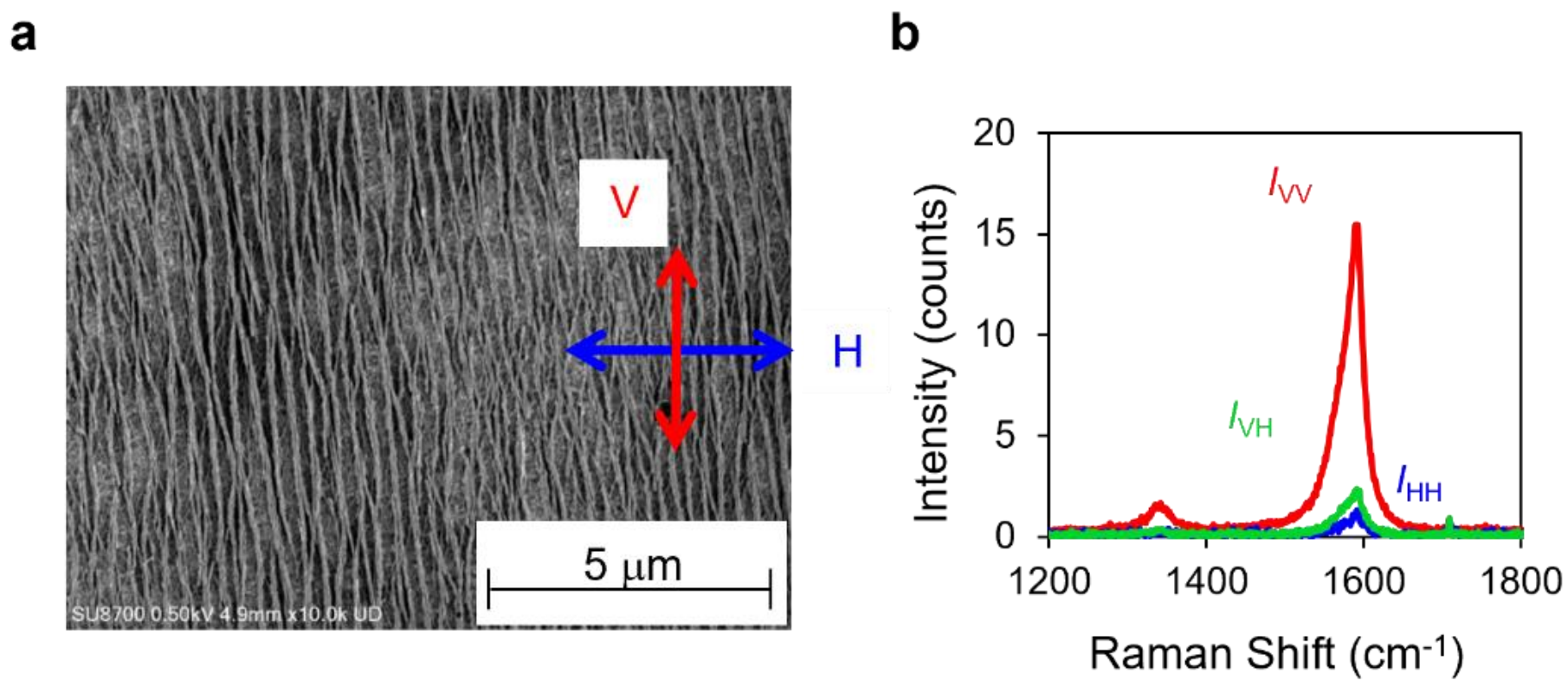

Fig. R3-7| Evaluation of alignment degree through polarised Raman spectroscopy. a, b, (a) SEM image, (b) Raman spectra measured in VV, VH, and HH polarisations (polariser and analyser are parallel or perpendicular to the alignment axis).

Applying the experimental data to Equation R2 yielded a 2D order parameter *S* of 0.76. This value is marginally lower than the surface alignment value (*S* = 0.93) determined by image analysis (Figure R3-6), suggesting that alignment degree of CNTs decreases with increasing film depth, possibly due to the strong surface alignment induced by laser alignment.

The manuscript has been amended as follows:
i) We have added polarised Raman spectroscopy in Figure 5.
ii) We have calculated the nematic order parameter *S* and described it in the main text.

7) I am not fully convinced by the G/D ratio interpretation. When nanotubes are aligned, the G band and RBM intensities increase due to polarisation selection rules, whereas the D band does not change significantly. The observed increase in G/D (e.g., at 125 mJ/cm²) may therefore arise from anisotropic enhancement of the G band rather than a reduction in defect density. A fair measurement would be to record both horizontal and vertical configurations, remove the analyzer and half-wave plate, average the spectra, and then calculate D/G to separate polarisation effects from defect density.

Thank you for this suggestion. We examined the underlying factors contributing to the enhanced G/D ratio observed after laser irradiation by conducting two sets of experiments: (i) Raman spectroscopy on laser-unaligned CNTs subjected to circularly polarised laser irradiation, and (ii) nanoscale analysis using TEM. Figure R3-8 presents the SEM image and Raman spectra for CNTs exposed to a circularly polarised laser, using the same wavelength, pulse width, and fluence as the linearly polarised laser detailed in the main text. The key distinction lies in the use of circular polarisation, which enables CNT absorption of the laser from all orientations, thereby preventing alignment and resulting in ablation of all CNTs.

The progression of Raman signals with increasing laser fluence is summarised in Figure R3-8 (h–k). According to the SEM images, low fluence irradiation produces a non-ablated, somewhat slightly clearer CNT structure (<100 mJ/cm², Phase 1). As the fluence increases (125–150 mJ/cm², Phase 2), ablation is initiated, and ultimately, the material transitions to carbonized debris at higher fluences (>200 mJ/cm², Phase 3). Raman analysis across these phases reveals that both the G-band and RBM peaks intensify during Phase 1, signifying an increase in the G/D ratio. However, as ablation progresses through Phases 2 and 3, the G/D ratio decreases, eventually falling below that of pristine CNTs in Phase 3. Notably, this trend mirrors the behaviour of laser-aligned CNT samples. These findings indicate that the enhanced G/D ratio following laser irradiation is not attributable to the alignment of the CNT structure.

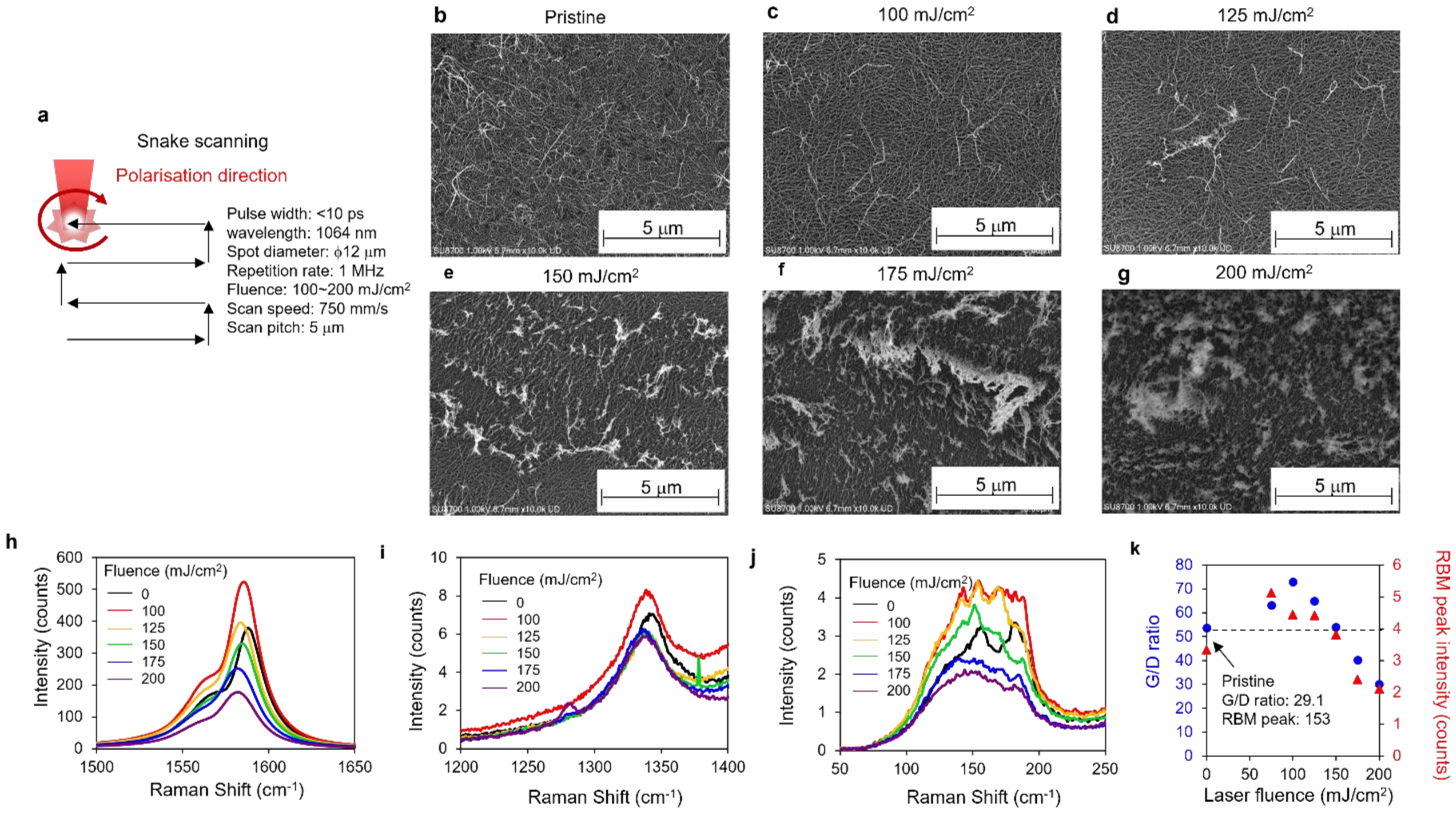

Fig. R3-8| Raman spectroscopy of laser-unaligned CNTs. **a**, Schematics of the laser irradiation condition. **b–g**, SEM images of CNTs irradiated with laser fluence of (b) 0, (c) 100 mJ/cm^2, (d) 125 mJ/cm^2, (e) 150 mJ/cm^2, (f) 175 mJ/cm^2, and (g) 200 mJ/cm^2. **h–j**, Enlarged Raman spectra of the (h) G band, (i) D band, and (j) RBM peak. **k**, Change in G/D ratio and RBM peak after laser irradiation for each fluence.

We conducted TEM observations to examine the effects of laser irradiation on CNTs to investigate the mechanism behind the improved G/D ratio. We used the same TEM grid throughout, with results from low-fluence laser irradiation presented in Figure R3-9(a-g). The fluence was below the ablation threshold; therefore, the CNTs remained intact after exposure. Notably, the dispersant associated with the CNTs was removed post-irradiation. Comparative analysis of magnified images before (Figure R3-9(e-g)) and after (Figure R3-9(b-d)) irradiation confirmed the removal of the dispersant.

The SEM image further indicated that low-fluence irradiation yielded a slightly clearer CNT structure (Figure R3-8(c)), attributed to the dispersant's removal by the laser. Fractures observed at increased fluence are detailed in Figure R3-9(h-k), with blue circles indicating CNT fracture points. The CNTs exhibited clean, localised fractures characteristic of their lattice structure, without significant defects outside the fracture regions. With a 1 μm laser spot irradiating the entire CNT, fracturing occurred precisely rather than as an entire disintegration, which is particularly noteworthy.

We propose the following explanation: Laser irradiation excites electrons within the CNTs, which tend to localise around pre-existing defects. This results in locally elevated electron temperatures and subsequent increases in thermal kinetic energy. This process initiates ablation primarily at defect sites. Analogous electron localization has been reported in materials like graphene (Xu, M. et al. *Phys. Rev. Lett.* **131**, 186202 (2023); Ishikawa, R. et al. *Nat. Commun.* **9**, 3878 (2018)), supporting the notion that fracture progresses preferentially from defective CNTs. Based on these TEM observations, we conclude that: i) laser irradiation removes the CNT dispersant, yielding isolated CNTs, and ii) fracture

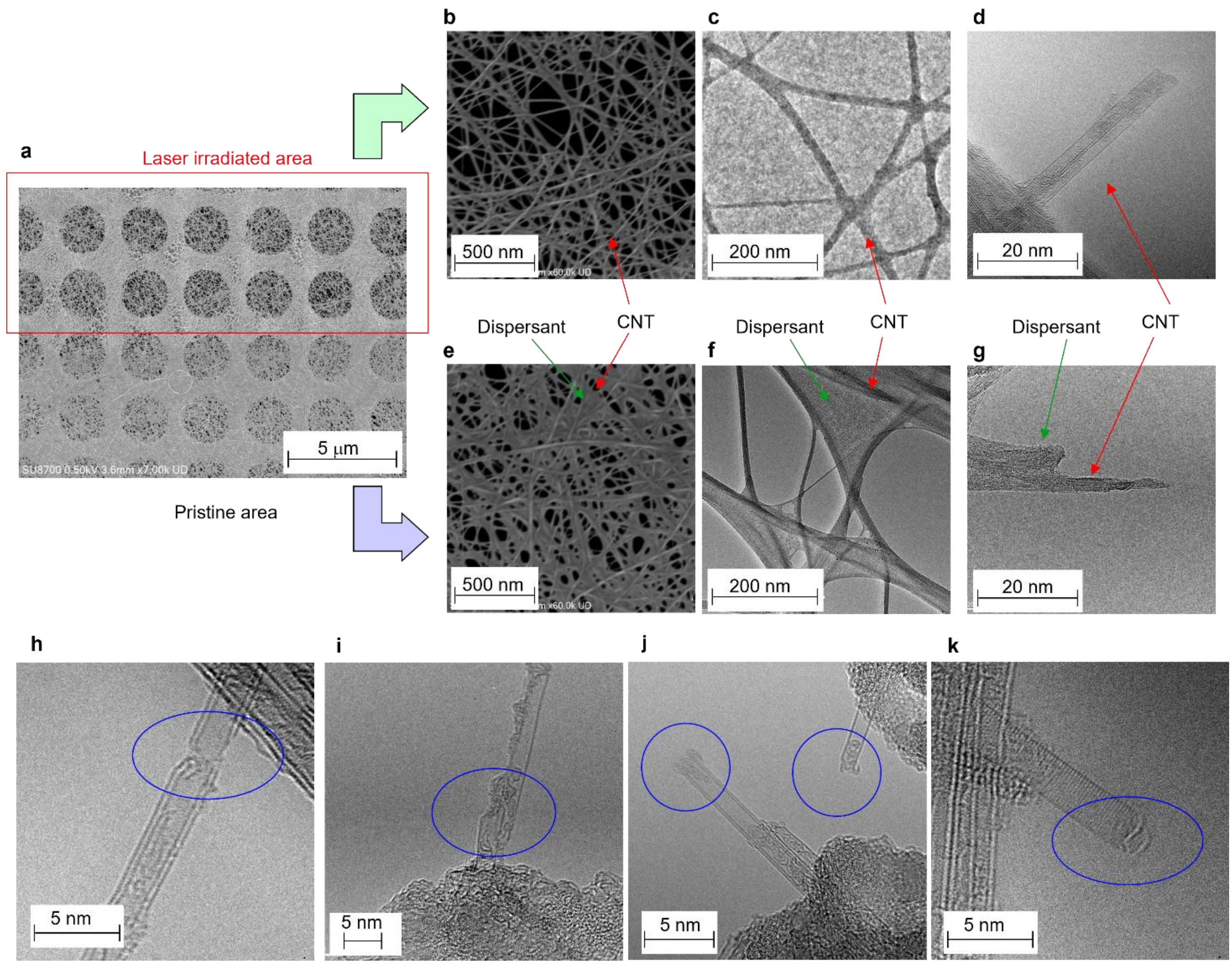

Fig. R3-9| SEM and TEM images of laser-irradiated CNTs. a–g, (a) Overview of the entire region, and (b–g) magnified views of CNTs subjected to low fluence laser irradiation. **h–k**, CNTs subjected to high fluence laser irradiation. The blue circles indicate the laser-induced fracture points.

initiates at defect sites, thereby enriching the proportion of highly crystalline CNTs. We hypothesise that the enhanced G/D ratio arises from dispersant removal and selective elimination of defective CNTs. This is a major and compelling finding, suggesting that laser irradiation may control CNT alignment and serve as an effective purification method, increasing CNT purity by removing dispersants and structural defects. For instance, this technique could potentially improve subthreshold swing and hysteresis in CNT transistors via non-thermal dispersant removal. Further experiments regarding CNT purification through laser irradiation are planned, and we intend to present our results in a subsequent publication.

The manuscript has been amended as follows:

i) We have added the results of Raman spectroscopy of laser-unaligned CNTs in Supplementary Figure 8.

ii) We have added the TEM images in Figure 4.

iii) We have added the discussion above to the main text.

8) Did the authors attempt to defocus the laser beam or adjust the focal plane (e.g., by changing the objective distance) to process deeper into the film? This could potentially enable alignment of thicker films.

Figure R3-10 illustrates the electrical and optical anisotropy of laser-aligned CNTs processed using lenses with varying depths of field. Figure R3-10(a) presents results for an objective lens with a numerical aperture (NA) of 0.28 and a depth of field of 3.5 μm, consistent with that used in the main text. As electrical and optical anisotropy is imparted to CNT films with a thickness of ≤250 nm, the effective processing depth is determined to be 250 nm. Figure R3-10(b) presents data obtained using an f-Θ lens with a 100 μm depth of field. Despite a thirtyfold difference in depth of field, the threshold film thickness at which anisotropy emerges remains constant at 250 nm. These findings suggest that the processing depth for laser alignment is independent of the lens characteristics or focal positioning.

We have revised the manuscript by including the optical and electrical anisotropy of the laser-aligned CNT film irradiated with different lenses with varying depths of field in Supplementary Figure 9.

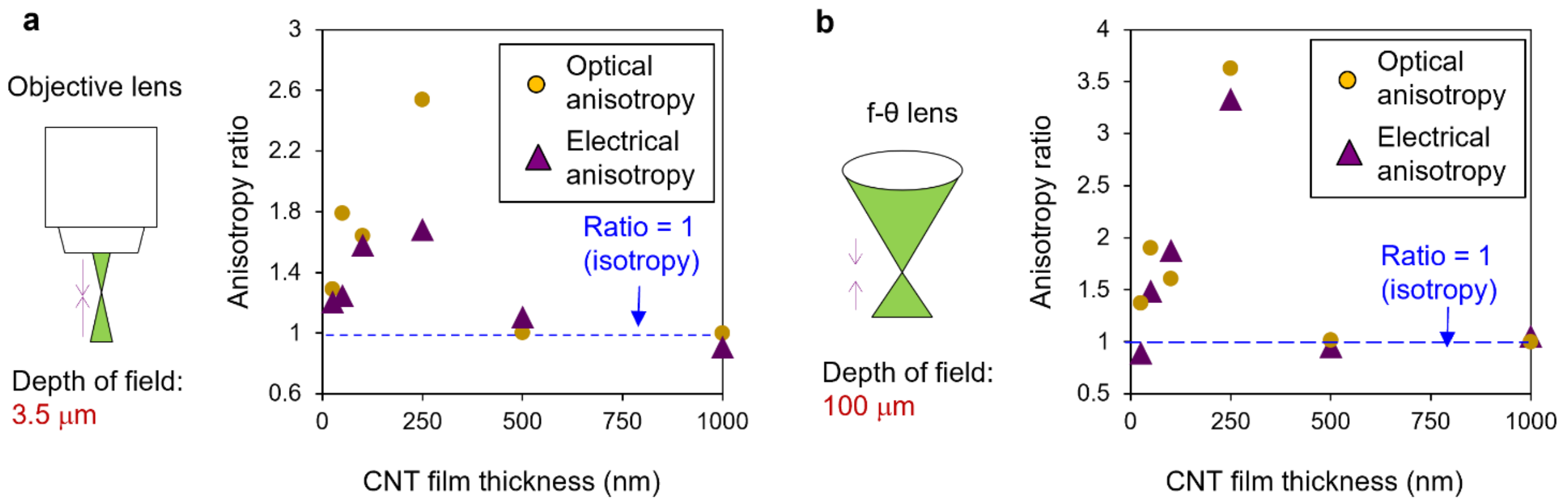

Fig. R3-10| Optical and electrical anisotropy of the laser-aligned CNT film irradiated with different lenses with varying depths of field. a, Optical and electrical anisotropy of laser-aligned CNTs via objective lens. **b**, Optical and electrical anisotropy of laser-aligned CNTs via f-Θ lens.

9) The statement that "conventional alignment techniques typically produce excessively thin aligned CNT films of ~10 nm" is not accurate. CVD [1] can produce aligned films at the micrometre-thick scale, Langmuir–Blodgett methods [2] at hundreds of nanometres, and controlled vacuum filtration [3] at tens to hundreds of nanometres. This statement should be revised.

Thank you for sharing this exceptional paper. We have referenced it and included the relevant description to reflect its content.

10) Although ultrafast irradiation is expected to suppress thermal effects, the observed deformation and waviness of the bundles suggest possible mechanical stress or residual heating. A brief discussion of this point would help clarify the processing mechanism.

Thank you for this suggestion. We identified two primary patterns in the formation of alignment structures: (i) when carbon nanotubes (CNTs) that do not absorb the laser remain intact and form the structure, and (ii) when residual CNTs intertwine as bundles to constitute the alignment structure. Our observations indicate that these mechanisms are influenced by material factors (CNT length and density) and optical parameters (laser fluence and spot diameter). Figure R3-11 illustrates the resulting alignment structures under varying conditions. Specifically, Figures R3-11(a, b) demonstrate that a spot diameter shorter than the CNT length or low CNT density leads to the persistence of individual CNTs or bundles without further intertwining. Conversely, Figures R3-11(c, d) reveal that a large spot diameter or high CNT density promotes the twisting of remaining CNTs due to laser exposure, resulting in an alignment structure. Therefore, the formation mechanism is variable and contingent upon material and optical conditions. The underlying causes—such as mechanical stress, residual heating, or shock waves during ablation—remain unclear and require further investigation.

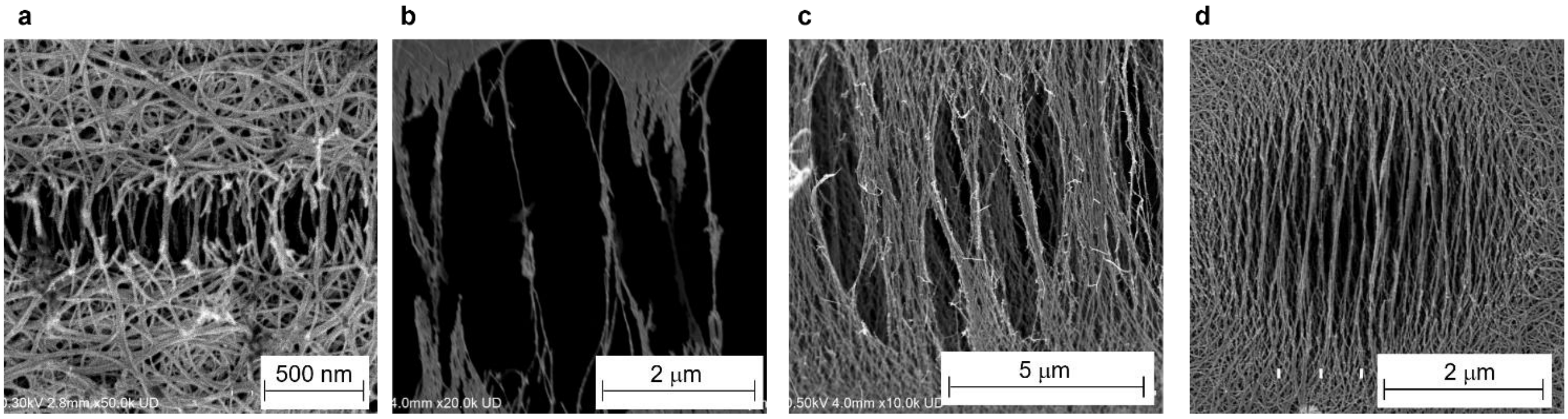

Fig. R3-11| Alignment structure when the density and spot diameter of CNTs were changed. a, Condition where the spot diameter is smaller than the CNT length. **b**, Conditions with low CNT density. **c**, Condition where the spot diameter is larger than the CNT length. **d**, Conditions with high CNT density.

We evaluated the influence of oxygen through thermal oxidation by conducting additional experiments under varying processing atmospheres—specifically air, argon, and vacuum conditions (Figure R3-12). All the experiments were performed under consistent laser irradiation parameters (pulse width: 200 fs; wavelength: 1030 nm; fluence: 125 mJ/cm²). Figures R3-12(a–c) present magnified images of laser-aligned CNTs, while Figures R3-12(d–f) depict the interface within the alignment region. The processing quality was comparable between air and argon; however, samples processed under vacuum exhibited exceptionally clean and refined alignment structures. Notably, only CNTs aligned under vacuum demonstrated seamless interfaces devoid of burrs. These findings indicate that (i) because it is a non-thermal process, the gas species (e.g., oxygen) does not affect the alignment quality, and (ii) the presence of gas molecules tends to compromise quality.

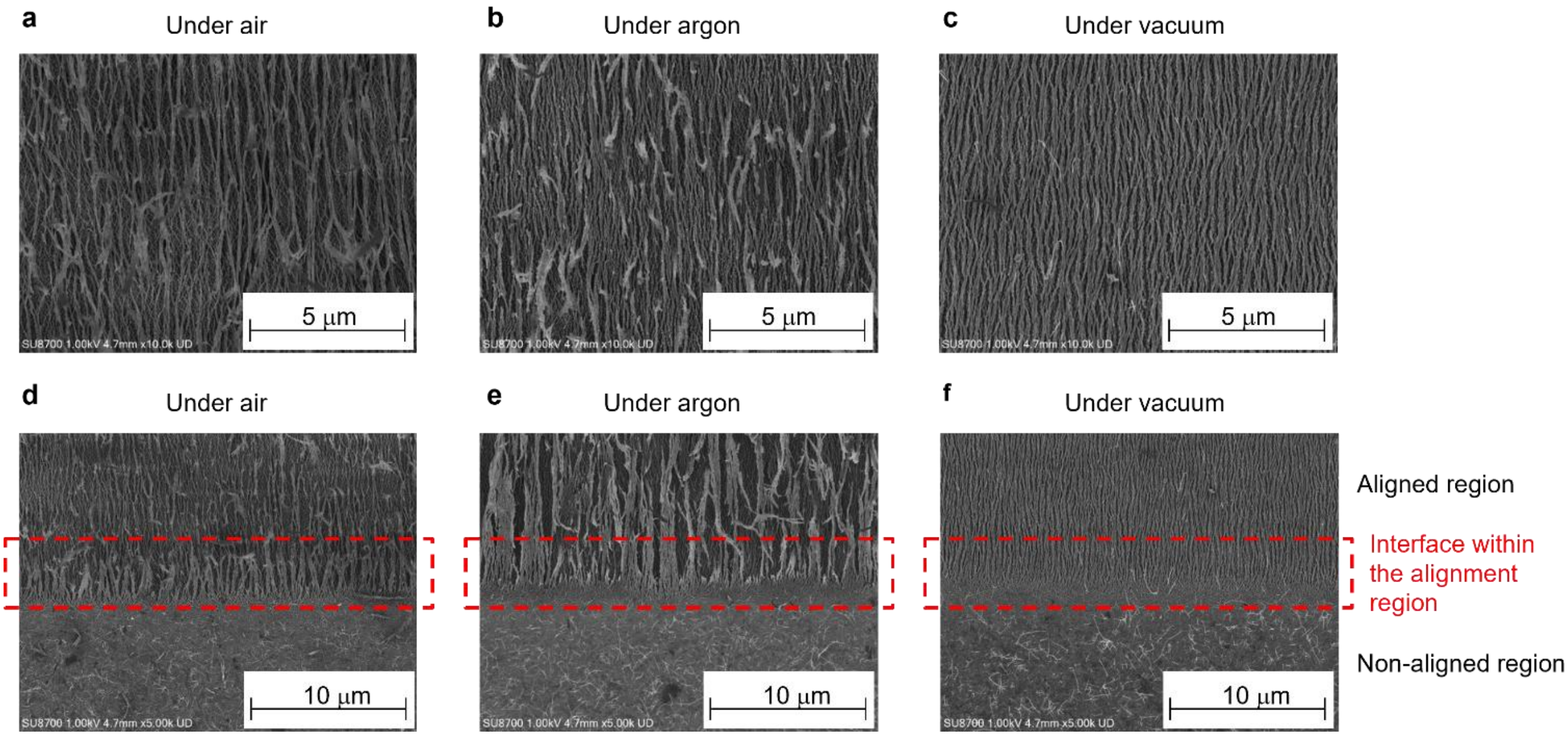

Fig. R3-12| Comparative results under different atmospheres. a–f, (a–c) Magnified images and (d–f) interface images of the laser-aligned CNTs performed under (a, d) air, (b, e) argon, and (c, f) vacuum.

The manuscript has been amended as follows:

i) Variations in alignment structure resulting from changes in spot diameter and CNT density are detailed in Supplementary Figure 11.

ii) Details on the influence of the processing atmosphere on sample characteristics are provided in Figure 1(j, k) and Supplementary Figure 3.

11) While electrical conductivity anisotropy is reported, it would be useful to compare the values with those in the literature.

Thank you for your suggestion. In the Conclusions and Future Prospects section, we presented the optical anisotropy (2.5@610nm, 2eV) and electrical anisotropy (3.4) observed in laser-aligned CNTs and conducted a benchmark comparison against prior studies (X. He, et al. *Nat. Nanotechnol.* **11**, 633 (2016)). Additionally, as addressed in response to comment 3, we clarified that the packing density achieved via laser alignment technology is primarily influenced by material properties rather than the process parameters. We also discussed the potential for producing high-quality, locally aligned CNTs by integrating laser alignment with conventional alignment methods.

12) Why are BNNTs used? If they serve as reinforcement in the composite, this should be clarified.

Thank you for pointing this out. The impact of BNNT on laser processing has yet to be comprehensively studied; therefore, presenting findings related to BNNT at this stage would not be appropriate. The section addressing chirality-selective ablation has been omitted from the main manuscript. Consequently, all experimental data involving BNNT have also been excluded from the text.

13) While the evidence for chirality enrichment based on absorption is valid, it may be affected and shadowed by inhomogeneous broadening and substrate effects. I advise the authors to perform Raman RBM measurements to provide clearer confirmation of chirality selectivity. For example, the presence or depletion of (6,5) at ~307 cm^{-1} and (7,5) at ~282 cm^{-1} would offer more direct and compelling evidence. Ideally, these measurements should be performed on the same location before and after laser processing to demonstrate selective removal or enrichment.

We appreciate your insightful feedback on chirality-selective ablation. The section on chirality-selective ablation has been omitted from this manuscript; however, we will carefully consider your comments in guiding our future research.

14) I recommend that the authors present AFM measurements of the aligned films to characterize morphology and surface roughness. AFM would provide quantitative information on bundle width, height distribution, film uniformity, and potential laser-induced topographical changes, which are not accessible from SEM alone.

Thank you for your suggestion. For the subsequent experiment, AFM measurements were conducted. Figures R3-13(a, b) present the SEM and AFM images of the CNT prior to laser irradiation, while Figures R3-13(c, d) display the SEM and AFM images after laser exposure. The alignment structure induced by the laser is observable in the SEM and AFM images. Pre-irradiation, the height profile was 0–120 nm, whereas post-irradiation, it increased to 0–350 nm. Due to the superior resolution of SEM compared to AFM, the SEM image is featured in the main text, with the AFM image provided in Supplementary Figure 1.

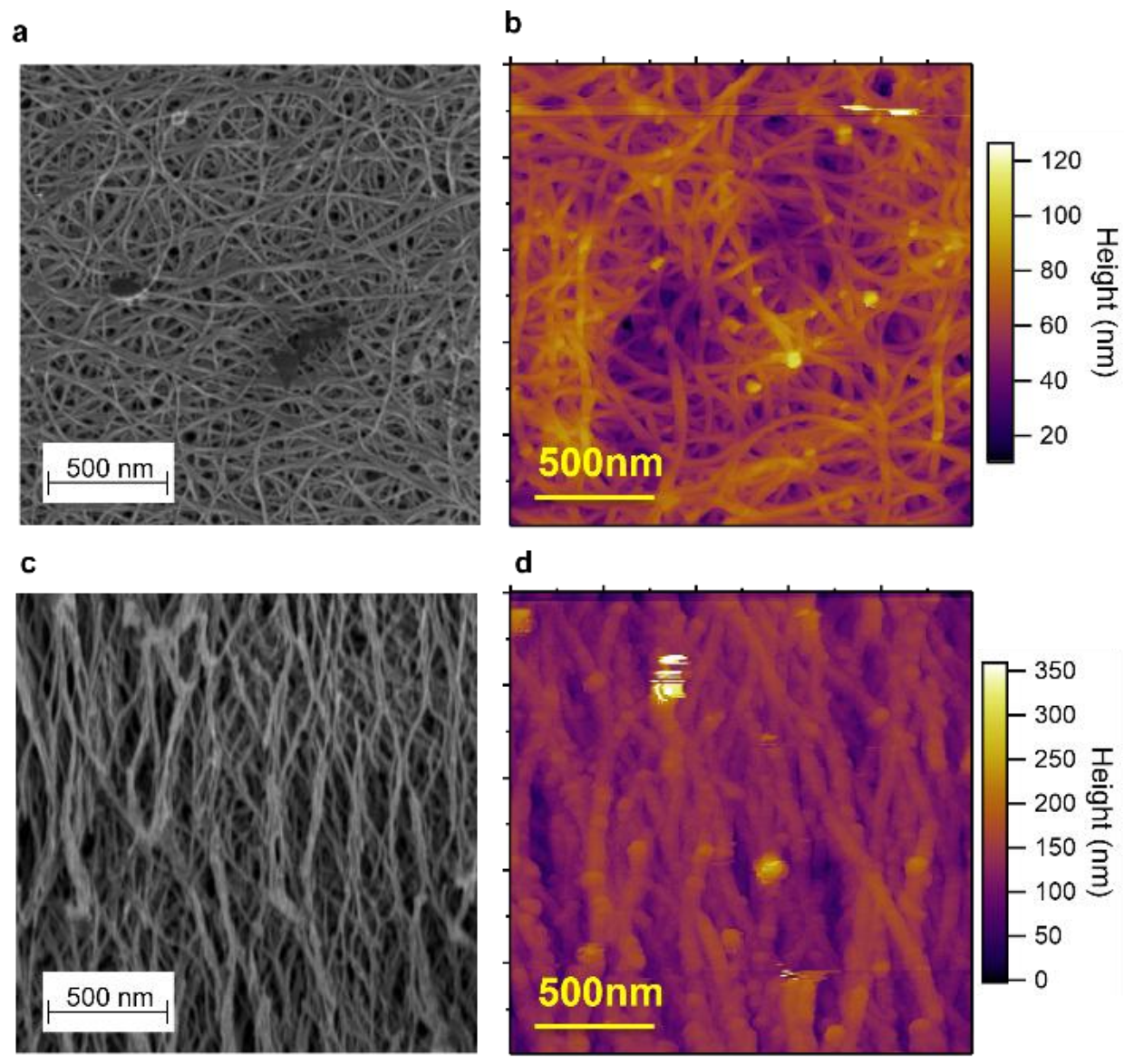

Fig. R3-13| AFM images of the laser-aligned CNTs. a, b, (a) SEM image and (b) AFM image of pristine CNTs. **c, d,** (c) SEM image and (d) AFM image of the laser-aligned CNTs.

Responses to Reviewer's Comments

Thank you for giving me the opportunity to submit a revised draft of my manuscript titled **Ultrashort-pulse optical post-processing for local alignment control in carbon nanotube films** to *Nature Communications*. We appreciate the time and effort that you and the reviewers have dedicated to providing your valuable feedback on this manuscript. We are grateful to the reviewers for their insightful comments on our paper. We have been able to incorporate changes to reflect most of the suggestions provided by the reviewers. We have highlighted the changes within the manuscript.

Here is a point-by-point response to the reviewers' comments and concerns.

The subsequent page contains detailed responses to the reviewer's comments.

To Reviewer 1

The revised manuscript is much improved according to the reviewers' comments. The authors answered my questions and added corresponding discussions with supplemented data. The revised manuscript can be recommended for publication in Nature Communications.

We sincerely appreciate the reviewer's recommendation for publication in *Nature Communications*. We are particularly grateful for the reviewer's insightful comment that thermal diffusion following laser absorption is a key factor governing successful alignment. This comment addressed an essential aspect of our work and greatly helped us improve the quality and clarity of the manuscript beyond its initial submission. We feel fortunate to have received such constructive and thoughtful feedback from Reviewer 1, and we thank the reviewer for their careful evaluation of our manuscript.

To Reviewer 2

1) The authors need to more carefully examine and discuss in the paper the literature characterizing the ultrafast emission of phonons in carbon nanotubes after optical excitation.

We sincerely thank the reviewer for giving us the opportunity to reconsider the mechanism of CNT removal. As the reviewer correctly pointed out, Refs. 37 and 38 discuss ultrafast electron–phonon scattering on a sub-100-fs timescale, whereas this process was not sufficiently addressed in our original manuscript. We have carefully considered this important comment and have re-examined the mechanism underlying laser-induced alignment processing of CNT films, as described below.

The first key point is the distinction between optical-phonon scattering and acoustic-phonon scattering. The scattering processes that occur after optical absorption, as well as the subsequent pathway toward thermal diffusion, have been discussed in the following studies.

[R1] I. Al Keyyam, et al., "Direct Observation of Suppressed Optical–Acoustic Phonon Energy Coupling in Supported SWCNT at Cryogenic Temperatures." Adv. Sci. ***12****, e09005 (2025).*

[R2] R. Wang, et al., "Distinguishing Optical and Acoustic Phonon Temperatures and Their Energy Coupling Factor under Photon Excitation in nm 2D Materials." Adv. Sci. 7, 2000097 (2020).

Based on these reports, after a material absorbs optical energy, electrons are first excited through photon–electron coupling. The excited electrons then transfer energy to optical phonons through electron–phonon scattering. This process has been reported to occur on an ultrafast sub-100-fs timescale [37, 38]. It should be noted that optical phonons play an important role in interactions with electromagnetic radiation (light), but they contribute only minimally to thermal diffusion. The energy stored in optical phonons is subsequently transferred to acoustic phonons through phonon–phonon scattering. This process has been reported to occur on a timescale of several picoseconds [37, 38]. Unlike optical phonons, acoustic phonons contribute directly to thermal diffusion in the material. Therefore, material removal on a timescale faster than acoustic phonon relaxation, i.e., within several picoseconds, is generally required for non-thermal processing [Ref. 33].

Based on these physical phenomena, aligned structures are expected to form when the laser pulse duration is 200 fs or 2 ps via non-thermal processing. In contrast, 20-ps or 2-ns pulses should not form aligned structures because the process is governed by thermal mechanisms. However, our pulse-duration-dependent experimental results (Figs. 1f–i) show that aligned CNT structures were still formed even with a pulse duration of 20 ps, which is longer than the acoustic phonon relaxation time in CNTs. This apparently unusual observation led us to conclude that the critical timescale governing successful alignment is not simply the electron-phonon relaxation time, but rather the thermal diffusion time between adjacent CNTs. This is the second key point. The acoustic phonon scattering process described above determines the timescale of the lattice temperature rise in laser-absorbing CNTs. In contrast, thermal diffusion between CNTs proceeds through dispersants, voids, and bundle contact

points, and therefore occurs on a much longer timescale than acoustic phonon scattering. Thus, successful laser-induced alignment is governed by whether the laser-absorbing CNTs can be selectively removed before heat diffuses to surrounding CNTs. The reason why aligned processing can be achieved with a pulse duration of 20 ps (Fig. 1h), which is longer than the acoustic phonon scattering time, is that although the lattice temperature of the laser-absorbing CNTs increases (thermal process), CNT removal occurs within a time scale shorter than the thermal diffusion into surrounding CNTs. However, because processing at 20 ps is governed by thermal mechanisms, the resulting processing quality is inferior to that obtained under non-thermal processing using pulse durations of 200 fs and 2 ps. Although the CNT–CNT thermal diffusion time is difficult to determine precisely because it strongly depends on the film morphology, including density, bundle diameter, and dispersant content, our experimental results in Figs. 1f–i indicate that when the relevant timescale is 10 ps or shorter, only the laser-absorbing CNTs can be removed before substantial heat diffusion to the surrounding environment occurs, thereby enabling the formation of aligned CNT structures. This consideration provides a more detailed explanation of the laser-induced alignment process and shows that our experimental results are not inconsistent with the ultrafast sub-100-fs electron–phonon scattering times reported in previous studies.

The reviewer's thoughtful comment therefore enabled us to reach a clearer understanding of the CNT removal mechanism. As also noted by Reviewer 1, the most important factor in laser-induced alignment processing is the selective removal of CNTs that absorb the incident light before heat diffuses to surrounding CNTs. We have added a detailed discussion of the removal mechanism described above to the revised manuscript.

2) Reconsider the mechanism (since the 2 ps pulses still show alignment but are much longer in duration than the sub-100 fs emission of phonons expected from previous experiments in literature.

As discussed in our response to Comment 1, we have carefully reconsidered the removal mechanism and have added a more detailed discussion to the revised manuscript.

3) The nanotubes parallel to the polarization are effectively removed in many of the cases in the paper leaving nanotubes perpendicular to the polarization. However, are nanotubes perpendicular also removed to some extend? Please quantify. For example, if a prealigned nanotube film were used as the starting point and the authors technique was used to remove a few misaligned nanotubes then would the aligned nanotubes decrease in packing density as a result (which would be undesirable).

We sincerely thank the reviewer for this meaningful and insightful comment. The question of whether CNTs oriented perpendicular to the laser polarisation are also damaged is essential for

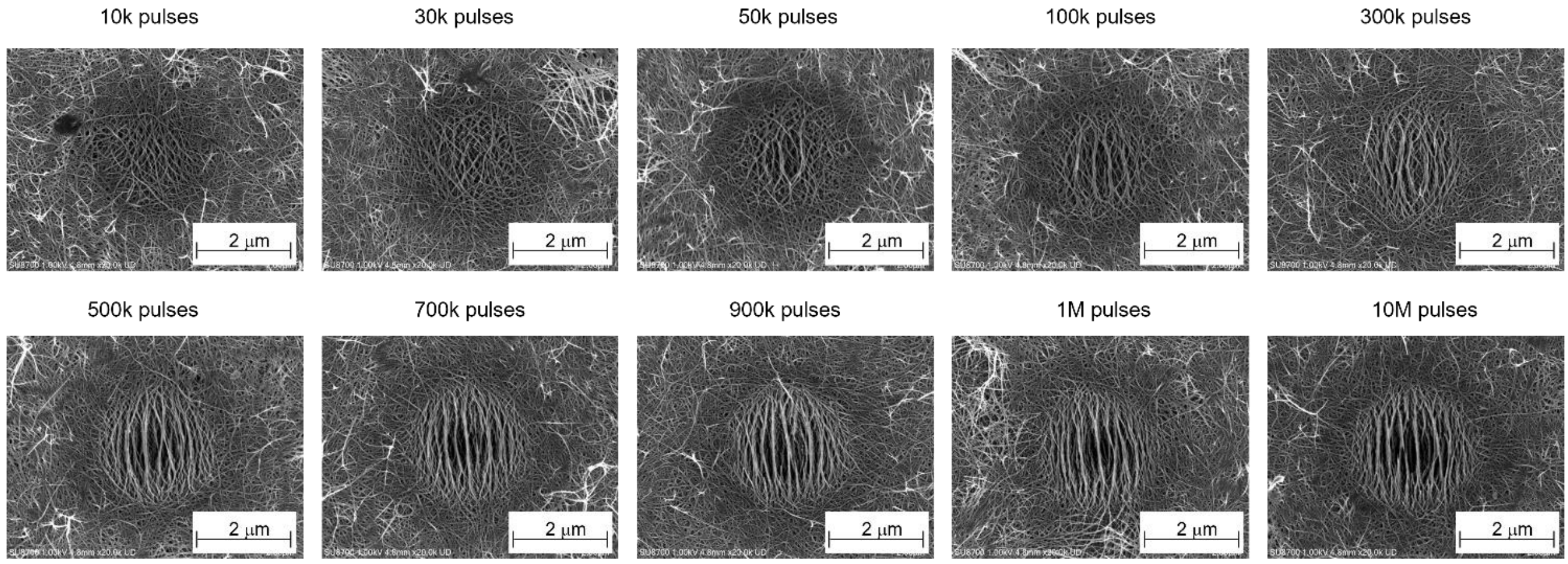

Fig. R1| Dependence on the number of irradiation pulses.

evaluating the practical usefulness of our technique. To assess possible damage to perpendicularly oriented CNTs, we performed additional experiments in which the number of irradiation pulses was systematically varied. The pulse-number dependence is shown in Fig. R1. The laser conditions were as follows: wavelength, 1030 nm; pulse width, 200 fs; fluence, 40 mJ/cm^2; and polarisation direction, horizontal. As the number of irradiation pulses increased, CNTs oriented parallel to the laser polarisation direction were progressively removed, resulting in the gradual formation of an aligned structure. At approximately 500k pulses, horizontally oriented CNTs at the surface were removed, leading to the formation of vertically aligned CNTs. Even when the number of pulses was further increased to 10M, the surface alignment structure was maintained, although material removal proceeded in the depth direction. These results indicate that CNTs oriented perpendicular to the laser polarization direction undergo negligible damage under the present irradiation conditions.

In response to the reviewer's comment, we have added these experimental results as Supplementary Fig. 14 in the revised manuscript.

The reviewer's suggestion enabled us to obtain an important result demonstrating that CNTs oriented perpendicular to the laser polarisation are not significantly damaged. This finding indicates that our technique can selectively remove undesired misaligned CNTs without reducing the density of CNTs aligned in the intended direction. As a next step, we plan to investigate the combination of conventionally fabricated aligned CNT films with our laser alignment technique to further improve alignment quality.

To Reviewer 3

I thank the authors for their thorough and constructive response to my comments. The revised manuscript has been substantially improved and strengthened.

In particular, I appreciate that the authors carefully addressed my concerns regarding the framing of the work relative to existing alignment techniques, the terminology associated with chirality control, benchmarking against the state of the art, the interpretation of SEM images as CNT bundle structures rather than individual nanotubes, and the characterization of alignment using additional quantitative methods.

I also appreciate the additional characterization that was performed in response to my comments, including polarized visible spectroscopy, image-analysis-based determination of the nematic order parameter, TEM characterization, polarized Raman measurements using the VV, VH, and HH configurations, AFM characterization, and the expanded discussion of packing density, alignment quality, and comparison with existing CNT alignment approaches.

Several of the newly added results were particularly interesting. The observation that processing under vacuum produces noticeably cleaner alignment structures than processing in air or argon provides an intriguing insight into the role of the environment during laser alignment. I also found the additional D/G ratio analysis and TEM observations compelling, particularly the evidence suggesting removal of residual dispersant and preferential elimination of defective nanotubes. These experiments substantially strengthened the discussion of the processing mechanism and provided valuable insight beyond the original submission.

The removal of the preliminary chirality-selective ablation claims and the corresponding revision of the title significantly improved the focus and clarity of the manuscript.

I also found the demonstrations of more complex alignment geometries, including radial and multi-orientation structures, particularly interesting from the perspective of future THz photonic devices and polarization-sensitive architectures. Such structures may also provide an attractive platform for future studies of anisotropic optical and electronic phenomena.

Overall, I believe the authors have satisfactorily addressed my concerns and substantially improved the manuscript. I support publication of the manuscript in its present form and recommend acceptance.

We sincerely appreciate the reviewer's recommendation for publication in *Nature Communications*. We are especially grateful for the reviewer's highly valuable comments regarding the evaluation of alignment quality. The reviewer's suggestion to assess the nematic order parameter enabled us to evaluate the degree of alignment in the laser-processed CNT films in a more universal and quantitative manner. In addition, comparison with the review articles recommended by the reviewer allowed us to clarify that the CNT-aligned films fabricated using our technique achieve a degree of alignment comparable to that of previously reported CNT-aligned films. These suggestions were extremely important for clearly conveying the significance and usefulness of our technique to a broad readership.

We are very pleased that our manuscript was reviewed by Reviewer 3, who has extensive expertise in CNT-aligned films. We sincerely thank the reviewer for the valuable comments that helped us improve the quality of our manuscript.

I would like to congratulate D. Suzuki et al. on this innovative work on the alignment of carbon nanotubes and on a well-written manuscript with clearly presented data. The authors demonstrate a picosecond laser post-processing technique that enables local alignment of CNT films by exploiting the polarization-dependent absorption of photons. In this mechanism, co-polarized nanotubes experience stronger absorption and are preferentially ablated, while cross-polarized nanotubes remain, leading to directional ordering. The authors further show that this method can be applied over large areas and identify the processing conditions (pulse peak intensity, pulse width, and related parameters) required to obtain aligned regions. Film quality is discussed, and alignment is supported by THz-TDS spectroscopy and four-terminal electrical conductivity anisotropy. In addition, the manuscript presents an interesting concept of wavelength-selective processing to enrich specific chiralities by ablating nanotubes resonant with the pump wavelength while retaining others.

Overall, I recommend this manuscript for publication in Nature Communications after revisions. I would, however, like to see several clarifications, additional characterizations, and improved positioning with respect to the state of the art. Some claims should be reformulated, the physical mechanisms discussed in more depth, and the morphology and alignment metrics strengthened.

Comment 1

I do not agree with the statement: *"The spatial controllability of conventional control techniques is limited to the millimetre scale, which hampers their micrometre integration."* While this may apply to certain shear-based methods (e.g., LS and LB), dielectrophoresis, and controlled vacuum filtration, it does not apply to techniques such as CVD alignment [1], lithographically defined microscopic water features [2], the soft-lock method [3], or DNA nanotrenches [4]. I advise the authors to reformulate this statement while considering the relevant literature.

I also recommend citing recent reviews that summarize state-of-the-art alignment techniques:

Macroscopically Self-Aligned and Chiralized Carbon Nanotubes: From Filtration to Innovation, ACS Nano 2025

Y. Chen et al., *Controlled Assembly of Single-Walled Carbon Nanotubes into Thin Films and Their Applications*, Adv. Funct. Mater. 2025

Citations (in order):

[1] Pint, C.; Xu, Y.; Pasquali, M.; Hauge, R. *ACS Nano* 2008, 2, 1871–1878.

[2] Foradori, S. M.; Prussack, B.; Berson, A.; Arnold, M. S. *ACS Nano* 2024, 18, 8259–8269.

[3] Guo, Y.; et al. *Nat. Nanotechnol.* 2022, 17, 278–284.

[4] Sun, W.; Shen, J.; Zhao, Z.; Arellano, N.; Rettner, C.; Tang, J.; Cao, T.; Zhou, Z.; Ta, T.; Streit, J. K.; Fagan, J. A.; Schaus, T.; Zheng, M.; Shih, W. M.; Maune, H. T.; Yin, P. *Science* 2020, 368, 874–877.

Comment 2

I find the title somewhat confusing. The term *chirality control* may be interpreted as transformation of one chirality into another, whereas the work presented here corresponds more to selective enrichment or isolation. I advise a slight modification of the wording to avoid confusion with true chirality engineering, as discussed in this review:

Tang, D. M.; Cretu, O.; Ishihara, S.; et al. *Chirality engineering for carbon nanotube electronics*, *Nat. Rev. Electr. Eng.* 2024.

Comment 3

While the method shows a clear advantage in enabling in situ local alignment, it does not demonstrate superiority over other techniques in terms of morphology uniformity or packing density. When discussing the advantages, I invite the authors to benchmark their material against the state of the art. For example, DLSA yields ~200 tubes/µm [1], and controlled vacuum filtration ~1000 tubes/µm [2]. Where does the present technique stand in terms of packing density?

Citations:

[1] Liu, L.; Han, J.; Xu, L.; Zhou, J.; Zhao, C.; Ding, S.; Shi, H.; Xiao, M.; Ding, L.; Ma, Z.; Jin, C.; Zhang, Z.; Peng, L.-M. *Science* 2020, 368, 850–856.

[2] He, X.; Gao, W.; Xie, L.; Li, B.; Zhang, Q.; Lei, S.; Robinson, J. M.; Hároz, E. H.; Doorn, S. K.; Wang, W.; Vajtai, R.; Ajayan, P. M.; Adams, W. W.; Hauge, R. H.; Kono, J. *Nat. Nanotechnol.* 2016, 11, 633–638.

Comment 4

Meijo eDIPS CNTs have diameters of ~1.5–3 nm and cannot be resolved individually by SEM. I therefore advise the authors to describe the aligned structures as bundles or fibers rather than individual CNTs. It is also possible that multiple CNT orientations exist within an aligned bundle. One way to address this would be to isolate a single bundle and characterize it by TEM. I also recommend adding more details on the CNT material used.

Comment 5

Two techniques are used to quantify alignment. THz spectroscopy is very forgiving, as a 2 THz wavelength (~150 µm) is much larger than the CNT dimensions. Raman is highly local and probes only the top surface. I strongly advise the authors to characterize alignment using UV–Vis–NIR polarized absorption, ideally probing S11 and M11 transitions. To enable comparison

with the literature, the degree of alignment should be reported as the **nematic order parameter S**, which is the standard metric for nematic systems, as shown in:

Katsutani, F.; Gao, W.; Li, X.; Ichinose, Y.; Yomogida, Y.; Yanagi, K.; Kono, J. *Phys. Rev. B* 2019, 99, 035426.

Comment 6

In the Raman measurements, circularly polarized excitation is used. I advise using linearly polarized excitation and reporting I_{VV}, I_{HH} and I_{VH} along with the dichroic ratio δ, and extracting a local nematic order parameter S. The incident polarization can be controlled by rotating the sample relative to the incident linear polarization while probing the same spot. For details, see:

Zamora-Ledezma, C.; Blanc, C.; Maugey, M.; Zakri, C.; Poulin, P.; Anglaret, E. *Nano Lett.* 2008, 8, 4103–4107.

Comment 7

I am not fully convinced by the G/D ratio interpretation. When nanotubes are aligned, the G band and RBM intensities increase due to polarization selection rules, whereas the D band does not change significantly. The observed increase in G/D (e.g., at 125 mJ/cm²) may therefore arise from anisotropic enhancement of the G band rather than a reduction in defect density. A fair measurement would be to record both horizontal and vertical configurations, remove the analyzer and half-wave plate, average the spectra, and then calculate D/G to separate polarization effects from defect density.

Comment 8

Did the authors attempt to defocus the laser beam or adjust the focal plane (e.g., by changing the objective distance) to process deeper into the film? This could potentially enable alignment of thicker films.

Comment 9

The statement that *“conventional alignment techniques typically produce excessively thin aligned CNT films of ~10 nm”* is not accurate. CVD [1] can produce aligned films at the micrometre-thick scale, Langmuir–Blodgett methods [2] at hundreds of nanometres, and controlled vacuum filtration [3] at tens to hundreds of nanometres. This statement should be revised.

Citations:

[1] Li, X.; Zhang, L.; Wang, X.; Shimoyama, I.; Sun, X.; Seo, W.-S.; Dai, H. *J. Am. Chem. Soc.* 2007, 129, 4890–4891.

[2] Pint, C. L.; Xu, Y.-Q.; Pasquali, M.; Hauge, R. H. *ACS Nano* 2008, 2, 1871–1878.

[3] He, X.; Gao, W.; Xie, L.; Li, B.; Zhang, Q.; Lei, S.; Robinson, J. M.; Hároz, E. H.; Doorn, S. K.; Wang, W.; Vajtai, R.; Ajayan, P. M.; Adams, W. W.; Hauge, R. H.; Kono, J. *Nat. Nanotechnol.* 2016, 11, 633–638.

Comment 10

Although ultrafast irradiation is expected to suppress thermal effects, the observed deformation and waviness of the bundles suggest possible mechanical stress or residual heating. A brief discussion of this point would help clarify the processing mechanism.

Comment 11

While electrical conductivity anisotropy is reported, it would be useful to compare the values with those in the literature.

Comment 12

Why are BNNTs used? If they serve as reinforcement in the composite, this should be clarified.

Comment 13

While the evidence for chirality enrichment based on absorption is valid, it may be affected and shadowed by inhomogeneous broadening and substrate effects. I advise the authors to perform Raman RBM measurements to provide clearer confirmation of chirality selectivity. For example, the presence or depletion of (6,5) at ~307 cm^{-1} and (7,5) at ~282 cm^{-1} would offer more direct and compelling evidence. Ideally, these measurements should be performed on the same location before and after laser processing to demonstrate selective removal or enrichment.

Comment 14

I recommend that the authors present AFM measurements of the aligned films to characterize morphology and surface roughness. AFM would provide quantitative information on bundle width, height distribution, film uniformity, and potential laser-induced topographical changes, which are not accessible from SEM alone.
